# Supplementary material for: Lived experiences of informal caregivers of adolescents with bipolar disorder at Masaka regional referral hospital
Source: PLOS Ment Health. 2025 Jul 1;2(7):e0000351. doi: 10.1371/journal.pmen.0000351 (PMC12798243; doi:10.1371/journal.pmen.0000351)
Supplement: S1 File — It includes eleven transcripts labelled from C1 to C11. (PDF) [file pmen.0000351.s001.pdf]

A QUALITATIVE INTERVIEW ON THE LIVED EXPERIENCE OF CAREGIVER 1 (C1) OF AN ADOLESCENT WITH BIPOLAR DISORDER

Interviewer – I

Respondent – R

I: My name is xx, I am a mental health worker and also a student from Mbarara University of Science and Technology and I am studying Mental Health but at Masters level, before we go further, I would like you to tell me your name, your marital status, your age, your gender, let's start form there, l will be letting you know what I want you to talk about.

R: My name is C1, I am 38 years old, I am Female, I am a Muslim, I am not married, we separated, their father left in 2010, he went to Sudan, up to now he has never returned, he left when that child was in the top class.

I: Do you hear from him?

R: No, we have never heard from him again ever since the war started in South Sudan; from that time, we have never heard from him.

I: Sorry, what is your highest level of education?

R: I stopped in senior four.

I: In senior four, and you sit for final exams?

R: Yes, sir.

I: Ok, for how long have you lived with this adolescent?

R: Her entire life.

I: Her entire life.

R: Yes, sir.

I: Since she was young?

R: Yes, sir.

I: You have told me you are her mother.

R: Yes, Sir.

I: You said the father left in 2010. How old is the adolescent now?

R: 18 years.

I: Meaning he went even before the adolescent was 10 years.

R: He went when the adolescent was in the top class, the child was about 3 and a half to 4 years.

I: Ok, you as a caregiver of this adolescent with mental illness, and the illness she has sometimes manifests in two different ways, sometimes it may appear in form of depression, or having elevated mood or being irritable, I would like to hear from you by telling us what you have been through or what it means to take care of an adolescent who has mental illness like your daughter, on that note, I would like you to tell us what has been your experience ever since you started taking care of that adolescent**?**

R: Ever since she started being sick, she has been suspicious of me, her mother, and she was worried. She would tell me that something is telling her that her mother is a bad person, like your mother is bad, your mother is bewitching you. She told me that she could hear those voices in her ears, I asked her, my daughter, what is it that has happened you?, at first I thought she was just speaking as you know children because one time before she left school, she wrote me a letter and she gave it to a certain parent, I had not gone for visitation at her school by then. She had written that there were children who checked them and prophesied to her about her father. She asked me, "Do you know what took my father?" I asked her, 'Did you go to school to study or prophesy?' I further told her, "Do you know that you will be expelled from school?". Later, she came back home, as a mother in talking to your children as a way of disciplining them. I would observe that even if I told her to do something, she would not do it; she would think that whatever I was telling her to do, I wanted to harm her. I tried to talk to her not to think like that, but I saw that she was suspicious and worried**.**

I: She thought that you could harm her**.**

R: Yes, because they told her, and it had already reached her mind**.**

I: Who did you think told her that?

R: I thought it was her fellow student at school when she told me that. Then I heard on the radio, they were saying that some students use tactics to confuse their fellow students who are intelligent to miss their goals, not to concentrate on their studies, and pretend that they are performing sorcery on them. They tell them that your mother does this and that so that they go astray, because she was intelligent. So, I said it means that at school, fellow students are doing something bad to her, when she came back for holidays, I tried talking to her slowly by slowly. Later, I realized that she had stopped being calm and had started to be irritable. I would tell her something - she would become irritable and

disobedient. She would look at me straight in the eyes confidently, which was not there initially; she was a calm person. I would punish her, thinking that it was because of adolescence. She continued telling me that she hears voices telling her that I, her mother, bewitched her, that I caused the disappearance of her father. As you know, people tell you to do a lot of things, I went to different places, I took her to traditional healers but there was no difference. I took her to churches, they prayed; there was no difference. I started seeing her leaving home and getting lost. I would ask her, "Where are you going?" She would reply, "I also do not know". So, the situation continued like that until someone advised me to bring her to the hospital. I came, brought her here, and they did not tell me about her condition, but they just started her on medication. During that period, she would leave home and disappear, she would be violent, and we would catch her. After she started medication, she is not doing badly off but maybe the challenge is that she wants money so much. I do not know whether it's the condition she is in that makes her love money a lot.

Even if I tell her I am poor, I do not have money, she asks, 'Do you not have money?' She starts getting sweat from the head, because I have ever been in a good financial condition and they used to get everything. As you know, the world money reduced. For her, she continued thinking that I have money, it's just that I do not want to give her - that's what she thinks. Where there is money, she is very happy, but where there is no money, she starts getting worried and thinking a lot.

I: Ok, in a few words, how do you understand your daughter's illness? If a person asks you about the illness of your daughter, how would you describe it to them?

R: I do not know; I do not know.

I: What did the health workers tell you?

R: Nothing, they did not tell me. I have remembered something, I forgot to come with the medical reports they first wrote for us when we came to the hospital, I remembered as I was on my way, I would have come with them.

I: It's ok, we just want to hear from you.

R: They sent us to the laboratory, they took a blood sample, we came back, she started medicine, they told me one thing that she was to start medication, they told me that she would be well, that's all.

I: Right now, you just know that your daughter has a mental illness**.**

R: Yes, but I am also asking myself deep inside me, whether she will heal. There was a time when she stopped taking medicine as she was staying with her sister. She lied to her sister that she was taking it, yet she wasn't; her sister observed that she was going back to the same condition she was in at first. When asked, she insisted that she was still taking her medication, but she relapsed again. Last term, she even attended school for only one day, and she relapsed. When she reached school, she was shivering, and I asked her why, and she said nothing, sweating, and I did not know whether she was getting worried about academics**.**

I: Apart from that, when you came to the hospital, you were given medicine. How was the daughter's condition when she started taking it? How did you find that medicine**?**

R: She started getting weak, always sleeping, and even up to now, if you do not wake her up in the morning, she does not wake up because she is sleeping. When you go somewhere after she has taken that medicine and you leave when you lock the door, and you do not have the keys, you may spend the night outside, because you can call her and she cannot hear, yet she sleeps near the window. You can bang the window, but she won't hear until she wakes up after some time**.**

I: On the same issue, as the mental health workers were starting her on drugs, did they explain to you what the drugs do, some of the side effects?

R: Yes, they told me that she will sleep too much, sometimes she may lose her appetite, and that I will buy her some tablets for appetite, and also eat too much, feeling hungry all the time**.**

I: Ok, when she received the drugs, is there any change you observed?

R: Yes, she changed, she changed because now she can be in a boarding school and stay there, and coming here, she came from school to get medicine.

I: She will get it**.** When she is at school, how does she take the medicine?

R: It's the matron who keeps it; she is the one who gives it to her

I: It means that the school leaders know about it**.**

R: Yes, I explained to them, because even the other term, she went to school for only one day, and relapsed, she came from school**.**

I: Ok, because I was getting worried about how she is allowed to take the medicine**.**

R: No, they know it. Another problem she has is that if she decides that I want this, that is what I have to do; if I disagree, she does not accept, she becomes annoyed, and then she becomes depressed.

I: As you have explained to me some things, I would like to know, some people sometimes get difficulties, especially when they have such an adolescent with mental illness. In most cases, we know that mental illness is not like a fever that it has come today and next week you will be fine. What challenge have you experienced as an individual?

R: I got the challenge at the initial stages, when we were trying to catch her, I could see that she does things intentionally. I thought that maybe she does it when she is in her right state of mind, and it could also disturb me. Another thing I got from her is that whenever I see her, I also become anxious, and I also start to sweat.

I: When did she behave that way?

R: Even right now, it is still there. I even thought I had hypertension, I would sweat, just seeing her, when she comes back from school, or she calls me on the phone. When she calls, I first ask her; Are you doing well? When I am anxious, in fear of what may be happening to her, I get into the situation of being anxious.

I: Would you like to think that it was due to what you saw, what she went through during that period?

R: I do not know; I get that fear.

I: How many times have you experienced that?

R: Even now, just thinking about it, I feel I am sweating.

I: Sorry, but relax, it can happen, especially when a person experiences a situation that was not easy, it can happen, and sometimes we do not want such things to happen to people we love, so sometimes when you see your person going through such a situation, you become anxious.

R: Because I had so much, I had so much hope.

I: That hope has to remain, do no say that I had hope because when you say I had hope, it's like you are saying that you no longer have hope, but we want that hope to remain because it's that hope which helps us to reach the things we have planned to do.

R: It's true.

I: I would like to know your experience with daily caregiving roles for that Adolescent?

R: The difficult experience?

I: It may be a difficult experience or not, but what do you experience as you try to take care of her, supporting her, since she has a mental illness**?**

R: Sometimes there are certain things she needs and I am not financially able to provide them to her, but when she needs them, sometimes I am forced to get into loans. I do not want her to become annoyed, and I do not want to tell her problems like the way I used to tell her while she was mentally well. I see that if I tell her I do not have, I see that I am going to make her annoyed, then I start going into loans, putting myself under pressure**.**

I: So, did you explain to the health workers because sometimes when such situations happen, it's good that they talk to you when you are together in a session. This helps you talk about some of the things and remove that fear which is always there, let's say between her and you. Some things, especially when you enter that clinical room, explain to the health workers about such issues, because there are certain things we see as hard. Since your daughter has a problem, the health workers can talk to her, so that she also understands that when my mother says this, it's true, I do not need to behave like this, and that**.**

Apart from saying that sometimes you get into loans, what other things have you experienced as you try to provide for her, and what other things have you experienced as you carry out your daily caregiving role to your daughter?

R: I support her like others

I: You support her

R: Yes

I: How is your relationship with her?

R: I think it's the illness that brought about change in the relationship; I cannot talk to her like I do with her siblings. When I tell her, let us first do this for your sibling, she gets depressed and worried, and she feels like I don't love her. I can tell her, should I give you food? And she refuses, yet she wants, and I tell her you will eat it, and she eats it, yet initially she had refused eating it, and I wonder why she refused at first, yet she wanted it**.**

I: Apart from that, how do you rate the relationship? Is it good in the middle?

R: The relationship between me and her is not bad as a parent and a daughter**.**

I: You relate well.

R: Yes.

I: Is there where you find that her illness makes it hard for your relationship with her?

R: As I have explained, she behaves like I have discriminated against her, like that.

I: What about the relationship between you and immediate family members?

R: Her family members?

I: Yes.

R: They do not care about me, their home is not far from here, I took her there, they just said sorry to me, and they told me to be strong as a woman. I did not receive any support from them.

I: Her father's family?

R: Yes.

I: What about their son not being there? What are they saying about it?

R: Nothing.

I: Do they think you know where he is?

R: No, they know I do not know where he is, I do not know whether they think I know where he is.

I: So, they do not care about the daughter.

R: They do not care, as a way of helping me, they would send me to church. That is where we used to go, they told me she would be prayed for and she would be fine, until someone told me to bring her to the hospital because she also had a relative with the same condition.

I: Was she also from church?

R: No, from another place.

I: Apart from that, there are people when they have a child with a mental health problem, the family rejects the child, they say, 'We do not give birth to children with mental problems', did you experience such?

R: No, they did not reject her, only that they did not provide support, but they did not reject her.

I: What about your relationship with community members?

R: It's not bad.

I: Don't they discriminate against you because of your daughter's mental health condition?

R: There was a neighbour, when my daughter had just gotten the illness, as you know, she would speak in parables, like saying that you are mentally ill, she would bring it that way because my neighbour has a mentally ill child.

I: Did she frequently say it, or did the sickness come when you already had some other disagreements?

R: No, there was no disagreement, but as you know, we were neighbours, the children were attending the same school, the neighbour's child never liked my child, despite being in the same class. My daughter was brighter than hers, so they didn't like her. It seems that the parent was feeling bad because my daughter attended school and was always number one, remember the school was near home. Other parents knew that their daughter was intelligent, because even in primary school she got a first grade. So, she used it as a song that my daughter is mentally ill, and whoever she could talk to, she could say that my daughter is mentally ill; she would ask others, 'Should we also take you to the hospital?'

I: She could tell it to you as a mother?

R: No, while talking to others, it does not make me feel good as a parent with a child with mental illness.

I: Apart from that, is there any other experience?

R: No.

I: Ok, apart from having a neighbour challenging you, sometimes talking negatively about the situation your daughter is in, is there anything else?

R: No.

I: Ok, how did you manage that?

R: To do what?

I: Managing that situation, whenever she could speak like that.

R: I used to just keep quiet.

I: Did you talk about it with some people?

R: There is my friend, my neighbour, I used to tell her about it.

I: How did she counsel you?

R: To keep quiet

I: What about your daughter, did she get to know about that situation that the neighbour was negatively talking about her?

R: At first, she did not know about it because she was mentally sick, sometimes she would act scared when she sees something about me that scares her, she would run back ward towards the home of that neighbour, she would run going to her home but when she stabilized, I told her and she understood it.

I: I would like to know your interface with mental health workers while offering treatment to your daughter? When you brought her, how was it? How did health workers treat you? What did they do?

R: I did not find any challenge because when we came, we first met a brown lady, a bit older in age, and she was the first we met, and she welcomed me well.

I: She welcomed you well only but what they did not do was explain to you about the condition your child is in, but they gave you the drugs and they told you about the side effects.

R: Yes, they told me that if she does not eat well, the drugs may negatively affect her

I: I would like you to tell me how long this illness has been there?

R: One and a half years

I: I would like to know before the illness came in, how she was, how was the situation and how your relationship with her was.

R: She was a quiet person, and I was proud that my daughter is a quiet person.

I: What about your relationship with her? How was it?

R: I did not have any problem because it is me who raised them when their father was not there.

I: When the illness came in, is there anything that first happened before the onset of the illness, did she first become physically sick, or was she beaten?

R: No, she was fine, she did not have any problem, but one day as I was seated, I asked them, everyone tell me what you want, they all told me, I want this, I want this.

I: Whatever they want you to do for them or to give them?

R: Whatever they felt they wanted, like for me, I want this. She was the last one to respond. I asked her, she kept quiet, I told her, Tell me, she kept quiet. When I insisted, she told me, I mostly want to see my father, I felt touched. I told her you have told me something

hard, she was in primary six by then. I asked her, ‘Is it what you want?’ She said yes, I told her he would come, but I felt touched as an individual because I was not expecting it, because she is a person of few words**.**

I: As you have said, the situation started slowly by slowly when she was saying someone told her this and that**.**

R: Yes, but I would tell her to do this, and she would do the opposite, do this, she does the opposite, when I tell her I will beat her, she would continue doing her thing, or sometimes beating her, and she would look at me consistently with anger. I would say I am beating something bad which is in you, so that it gets out of you, and she would look at me consistently, that's how she started, and I thought there is something wrong with this child**.**

I: So, when you came to the facility, you got the medicine, you went back home, and you started giving her the drugs. How was your experience because she was not taking daily drugs, but now you have to give her daily drugs**?**

R: Sometimes she would refuse to take it, she used to first refuse taking it, and I tried to talk to her, sometimes she would hide it. I explained to her and others explained to her why she needed to take medications, and I would lie to her by giving her false examples like now you see so and so, that one is also on medication, can you believe it, you see so and so, that one was also like that and she would accept to take it**.**

I: It’s good that today I have seen her, she came alone to get medicine, that is so good because there are some parents when they get such a child, want to do everything for their child. They forget that tomorrow, in case they are not there, can this child manage? So, thank you for empowering her so that she can do something**.**

Which challenges did you get after she had started taking medicine, she started getting treatment, which challenges did you get?

R: She became so weak and I feared she could become weak, started getting tremors, and giving her a cup, she could keep shaking hands, even when eating food, she could be trembling and weak**.**

I: What about her performance at school? How is it?

R: It changed; she no longer performs as she used to**.**

I: Is there a way it affects her because she no longer performs like she used to?

R: Yes.

I: How do you see it?

R: When she comes back like today, she tells me that she no longer grasps things like the way she used to. I ask her, 'What is the problem?' She says she also does not know, she wants, but she realized that she no longer understands like the way she used to.

I: When you compare the responsibility you used to have before she became sick and now that she became sick, is there any change, do you see anything that has been added, in that you have to work so much, time?

R: Yes, because every day, I have to send her to school with 1500 shs daily, not part of school fees, it is for eating because she wants to eat, and also other requirements. I used to give them 4 kgs of sugar each, but hers gets done too quickly. Remember, she wants to drink all the time.

I: Before she started taking drugs, were those things happening?

R: No, they were not there.

I: Which drugs does she take?

R: I do not know, I see pink, white, and blue tablets, she used to take yellow tablets, but they no longer give her those yellow tablets, I do not know whether it was for sleep for a short period of time, or pink, white, and blue.

I: You do not know the names of those tablets?

R: I do not know; she has the book where it's written.

I: You talked about that sometimes they told you to take her to church. I would like you to explain more. When you went to church, what happened? Your experience in church, when you went to church, what did people at church used to say?

R: No change, she continued with being with destructive behaviors.

I: What were they saying in church about the daughter's condition?

R: They just prayed, nothing they told us, they were chasing demons, they used to say demons had attacked her/spirits from her family. They would say they are chasing a way to the demons

I: Some pray while beating.

R: She was not beaten.

I: Some immerse in water so that it remains there.

R: She was not immersed.

I: But when they prayed for her, did you see any change?

R: I did not see any change, that's why I left after someone advised me to come to the hospital.

I: Sometimes when they tell you they are demons, family spirits, sometimes we are compelled to try out the other side.

R: I did all of them.

I: When you reached there, how was it?

R: There was no change.

I: What did they say?

R: Again, the same things, witchcraft, family spirits.

I: Are there any rituals they told you to do so that she heals?

R: They told me to do some rituals.

I: For example, like what?

R: That she needs to settle down, we needed to prepare "ebiibo" to appease her family spirits, so that she can improve mentally

I: Did you, do it?

R: I did not do them, it required a lot of money, I did not have the money they told me to give, even before that, in the past, my heart never liked such things, I felt like I cannot take all that money into that, she may not even heal, that would discourage me, I could tell myself, I get a loan to put in this, what if she does not heal.

I: How much money were they asking for?

R: The traditional healer told me to pay 1.5 million shillings, the one who asked for little money was the one of 700,000 shillings.

I: What recommendation can you give to other caregivers who have children with this condition like your daughter?

R: To come to the hospital, because she reached period of time when she was not even eating, she had lost weight.

I: Did you reach a point that sometimes you even feared leaving her at home?

R: Yes, I could not leave home.

I: What were you fearing at that time?

R: That sometimes I may go and she leaves home, disappears and fails to return home, because she had reached a point where she could get out of the house naked, she did not want to bathe, so it was like I was looking after a baby.

I: Were there moments when you feared for her siblings at home?

R: How?

I: When you feared that maybe she could harm her young siblings at home?

R: No, because she could not fight, she could not beat, but only wanted to leave the house and wander, but she was not fighting.

I: If you are to talk about what you have been through as a person who has a child with this illness, what can you talk about it? What challenges have you faced?

R: Talking about what I have experienced?

I: What challenges have you experienced as a parent with a child in such a condition?

R: People avoided me, the ones who used to be my friends and would visit me often were no longer visiting me because I had a child with mental illness - I had no one to run to for help.

I: Ok, another thing.

R: Maybe another thing, wanting some needs and I could not get them, I could spend most of the time on the child, sometimes I was not able to get food, I had nothing to do, all the money I had spent on her.

I: We had not talked about the issue of finances in detail. On that issue, I would like you to throw more light on that. What does it mean to take care of a child, especially when it comes to finances, when a child has an illness like your daughter's?

R: It takes a lot of money because remember what they want, they want it, they do not know anything else. If they tell you that I want this, they want it, sometimes I prepare food, and she refuses to eat it because she wants something else, and I do not want her to get annoyed.

I: What about medicine, is it always available? Do you face any challenges may be in buying it?

R: When I come here and they tell me it’s not in stock, I go look for money and I buy it.

I: Like today, she has come, if they tell her it's not in stock, she calls you and tells you, like now she came from school?

R: She first comes home.

I: I thought she came directly from school.

R: No, she first comes home, then I tell her to come to the hospital.

I: Ok, after does she also first come home before going back to school?

R: Yes, if money is not there, I first buy cheaper drugs, until I get money to buy expensive drugs, and then I send them to her.

I: What about fellow students, are there those who challenge her, laugh at her because of her situation?

R: No, she loves them and they love her, those were where we stay.

I: What about those at school?

R: They also love her; she does not have any problem with them.

I: She has not had any challenge with that.

R: Yes, they love her.

I: Now that she is a teenager, there is a question that I asked you, how she is treated in the community where you stay and you said she does not have any problem in that, she may reach a time let's say as you see she is a female, the age of adolescent, you may find her standing with a neighbor's son, has that ever happened? And how is she treated?

R: Since she is naturally a quiet person, she does not have such conversations. I have never heard it about her or seen her with anybody because even if her classmates come and pass by, she leaves and goes to the back of the house.

I: When she does not want to be seen where she stays, or just, she does not want to be seen?

R: I ask her why she does not want her classmates to see her. She says they are stubborn, she fears, and leaves, or she hides behind a counter. I have not seen anything beyond that.

I: Has she ever asked you when she will stop taking drugs?

R: Yes, she asked me, she told me that she saw fairly well was written, and she asked why am I still taking the drugs? I replied to her, 'You have to keep taking the medicine, they will be telling us.' She was asking when, I replied to her that I also do not know, but they will be telling us. She says ok.

I: She was too concerned; she saw that the medicine was becoming like a burden to her.

R: Yes.

I: Away from her, let me ask you as a parent, are there moments when it's hard for you, and it puts you under pressure wondering whether your daughter will heal?

R: Yes, because sometimes I am there thinking that maybe after my child grows up, she will go abroad for work, but with this illness, I do not think that she can go.

I: So, in brief, sometimes there are moments when you get worried about her future?

R: Yes.

I: What about things like marriage, are there times when they cross your mind?

R: I think about that too, and I say that after, will she heal, though she is fairly well, but there are moments when I see her frightened, or she sees someone and starts frequently asking me about that person. I ask myself whether this condition will completely clear, or sometimes, when I talk with someone, and she comes and stands there, stops whatever she is doing, and listens to the conversation. Sometimes she comes and knocks on the door, and she calls, but knowing that someone is inside conversing. Sometimes she does not call but leaves whatever she has been doing and comes to listen to the conversation when I am talking with someone, and I tell her, 'Haven't you been doing something?' then she goes back to what she was doing but wanting to stand there and listen to our conversation. Listening to what we are talking about. She first got worried, saying that she heard me talking about her, and she gets worried whether I am talking about her when I meet other people, that she has a mental illness, something like that.

I: What about this situation, like classes, the good thing you have said that you told the school leaders about it, how do they treat her at school?

R: They do not treat her badly because I have been having a problem, as you know the income of the woman, father is not there. I take care of everything, when time comes for paying school fees, she is sent back home, sometimes I would hide it from her that we do not have any debt at school, so that she calms down, because when they tell them to check themselves on a notice board list, if they have not cleared school fees, they should go back home, she starts speaking that they have sent her from school. When I do not have money, the pressure becomes high, I talked to the headmaster, and I told him not to show her that I have not cleared school fees because sometimes, let me say I have paid 100,000 shs, she pays 610,000 shs, I tell her that I have paid half of the school fees,

there she gets it that I have paid a lot of money, when the truth is that I have not paid all that money. So, I talked to the headmaster and I requested that to be changed, she should not be sent back home due to school fees, because I know I will fully pay that money, I requested him to call me instead of telling her to go back home, and that they should leave her**.**

I: As we conclude, I would like to know, the first time you came here to the hospital, when you reached and saw the environment here, you saw other sick people, how did you feel in your heart?

R: I became strong because I saw some had ropes, so many wounds, others shouting, others being returned to the ward by the askari, yet mine was not like that, and I became strong**.**

I: Some say my child will not be here**.**

R: Some people told me I did a bad thing to start her on medication, they did not see it as right, telling me that I would not have started her on those medicines, that the medicines are bad**.**

I: But when you do a flashback, what do you say about the medicine**?**

R: Nothing, maybe because she gets tremors and is weak**.**

I: For that you can talk to the health workers and see if they can adjust those doses, or they can reduce it or if it makes her weak during day, they can say that she takes it at night as she is going to sleep, all that can be possible, they can see a way of adjusting those doses**.**

R: OK**.**

I: Do you have any questions?

R: I want to ask, is it that the illness is causing my daughter not to perform well, because my daughter was intelligent**?**

I: I request to first stop the recorder from recording, then I answer the question. Thank you

A QUALITATIVE INTERVIEW ON THE LIVED EXPERIENCE OF CAREGIVER 2 (C2) OF AN ADOLESCENT WITH BIPOLAR DISORDER

Key

I: - **Interviewer**

R: - **Respondent**

I: I am Nurse xx; I will be interacting with you, and you will explain to me what you have gone through as a caretaker of this child. But before we proceed, I would like you to tell me your name, your age, your religion, and your marital status.

R: I am C2, I am a Muslim, 36 years old, and the father of the child is yy

I: Is the child your daughter?

R: Yes, she is my daughter, she is 14 years and 1 week old

I: Has she been living with you for all these past 14 years?

R: Yes, I live with her, and she has never left my home

I: Ok, how many children do you have?

R: I have six children, and she is the fourth born

I: Does any of the other children have a mental health problem?

R: None, no one has a mental health problem. To get this problem, it started when she was very young. she would sit in one place and would not play/be active like other children, I was forced to take her to the doctor for a checkup. The doctor informed me that she had a problem with bones, and that was the reason why she was behaving like that. She was started on treatment and even got better; however, when she made 1 year and 8 months, she developed seizures, and we rushed her to a medical facility in Lukaya. On healing from the seizures, her hand and that side of the body were paralyzed, but she had not realized it; we only realized it when she was having difficulties in movements. We took her to a rehabilitation centre and she got better. Three months ago, she developed malaria and her mental state deteriorated, and she even started undressing herself in public. Even yesterday, I noticed that she is reexperiencing the same symptoms that come when she is going to break down. When she is about to break down, she talks a lot, sings a lot, and spends the whole night talking. Whenever she presents with those symptoms, it drains me a lot; sometimes, she even wants to fight me. When she got this episode, the hand and the

leg had also become paralyzed, but after receiving treatment, she is greatly improving. It is really a bad experience for me. I gave birth to a normal child, but suddenly she became mentally ill, exhibiting abnormal behaviors, which puzzles me a lot. Indeed, it is very challenging; people give you varying advice, which ends up confusing.

I: Could you explain some of the advice that people give you?

R: Some people say that she was bewitched or that these are family spirits that need to be attended to. But ignored all that and decided to bring the child to the hospital for treatment; the drugs sometimes make her very weak. And also, when she misses eating food on time, she shivers a lot and drools saliva from her mouth.

I: Thank you so much for elaborating on those issues, which takes us to another question now. As a person who has been looking after this child, we would like you to tell us about your lived experience while caring for that child.

R: I have had a very bad experience with her. By the time she broke down mentally, her father was nursing fracture wounds following a road traffic accident involving a car that fractured his legs. Even right now, he is still on the clutches at home. So, during that time, I would worry about the child's mental state and at the same time the father's condition, which strained me a lot, but I managed somehow. I would like to ask a question: will my child ever get fine and stop medication?

I: We shall elaborate and answer that on that question as we proceed, because some of the things that I want to ask you relate to that.

R: Ok

I: So, before she broke down, how was she?

R: Before the mental illness, NK was a good person, but would always get angry very fast, and most of the time. When she would be disciplining her siblings, she would punish them heavily, something which we had not given much attention – we actually delayed realizing that she had a mental health problem. The time she totally broke down, it took me a month to realize that she had broken down. She used to perform well in class, but this time around she performed badly (she was in the last position in class). When she came home and I asked her, she insisted that she had won everyone in class. She insisted that she had led her class, that incident passed, and I didn't give it much attention – that month passed. One time I instructed her boil tea and put it in the flask like we normally

do, she responded by asking me whether we lacked hands to put water in the flask – she said ‘if you put the water in the flask by yourself, will you fall sick, will you develop a skin rash? That response perturbed me a lot, and I promised to beat her up; it took me time to realize that she had broken down mentally. On the very day that she broke down, I instructed her to dig around the compound, but she kept digging and putting the rubbish on the veranda of the house. On that day she actually did many unusual things. At around 7:00 pm, she annoyed me a lot, and I had to punish her by beating her up. It is at that time that I realized that she had broken down because, after beating her, she started saying that she was going to report to the police. Up to now, I sometimes ask myself and wonder if she has a mental health problem because of has had seizures earlier in life? Sometimes I also wonder if it is related to the problem of the bones that she suffered when young.

I: Earlier on, you had hinted at her birth

R: When I delivered her, she had 8.8 kilograms, and people kept saying that such children normally have diabetes. She spent one month and six weeks without opening her eyes, I had to bring her to the doctor for examination, who confirmed that she was healthy. However, the people who kept coming to visit me after birth insisted that she was not healthy. That is how I have lived with this child, and up to now I am not certain whether her mental health problem is a result of being overweight at birth or if it is related to having suffered a problem with the bones when young. I don’t know what caused her to become lame. Could it be because she didn’t receive treatment for the lameness?

I: Thank you so much for elaborating on those issues. So, I would like to hear from you, when she responded to you harshly like she did, how did you feel as a caretaker?

R: I felt very bad, and at that point, I didn’t realize that she had broken down; I was forced to beat her up/punish her. In fact, at that time, I thought to myself that she was being disrespectful and that I needed to correct her immediately by punishing her.

I: So, when you realized that she wasn’t feeling well (mentally unstable), what did you do next?

R: I brought her to the hospital for treatment because she had now started beating up people. Some people were advising me to take her traditional healers, but I didn’t listen to them. She was very aggressive, but luckily, she would listen to me whenever I talked to her. She was fighting, undressing, and walking naked in public. I don’t think there is a

traditional healer who could calm her down based on the state she was in at that time. I brought her tied up on ropes

I: Ok, so when you brought her to the hospital, what happened?

R: When I came, I found the doctor whom I talked to yesterday before coming here. He interviewed us and asked us many questions; she was later injected with some medication because she was aggressive. We spent four days here, after which we went home and then returned on appointment dates.

I: So, how did you find this hospital?

R: Maybe it could have been hard for me, but luckily, I have a sister who works in the pharmacy department; she is the one who directed me to the Doctor here for treatment.

I: So, when you came, did you find some patients who were severely ill compared to your child?

R: Yes, I found many patients who were more severe than mine

I: So, what came into your mind when you showed them?

R: You see, there were many, and some looked very responsible, a factor which made me believe that my child would get better. Based on how she was at the time of admission and how she is right now, I have a strong feeling that she will be fine. She can now spend a full week when she is feeling well and talking coordinated words.

I: What was your experience with the health workers?

R: The health workers treated me well, and I have no complaints about them

I: Ok, that is very good. So, still on health workers, after the long interview, what did the health worker say to you?

R: Truthfully, he did not tell me the outcome of the interview, nor did he inform me about the cause of my child’s mental health problem. Here I have explained to you how it all started, but he didn’t ask me those details

I: So, did he explain to you the disease that your child was suffering from?

R: He just told me that that disease exists, but up to now, I am not certain about the disease that my child is suffering from. In fact, I thought when I came to the hospital, they would check my daughter’s brain to see what the problem was, but up to now, I am not sure about the disease that my daughter is suffering from.

I: Now let us turn to drugs, what did the health worker say about the drugs?

R: He just wrote and instructed me on how to give her the medication; I kept coming back, and they would adjust the doses accordingly. I got a challenge when the doctor went on leave; those around just gave me medication, and when I took it, it made me feel bad. I had to come back, and when I came back, they again adjusted, but still, the medication treated her badly. So, when I came back and found him, he gave me medication, and the patient got better.

I: So, as of today, what medication is your daughter on?

R: Regarding that, you can even cane me, I don't know the type of medication that my daughter is on.

I: Did they talk about the side effects of the medication?

R: No, they gave the medication but did not explain the possible side effects. There is a time they gave her medication and she could drool a lot of saliva. I was forced to come back the following day, they gave me three tablets and instructed me to give her half a tablet of and she got better.

I: So, what can you say about the relationship between you and your child?

R: At first, she used to fear me a lot because at first, I wasn't aware that she was mentally sick, so whenever she made a mistake, I would punish her a lot. That is why I told you that even when she got sick, she would listen to me because she used to fear me. However, when I understood that she is mentally sick, I stopped punishing her and right now she is more free with her father. Actually, right now we are friends, and she doesn't fear me a lot.

I: I would like you to also comment on your relationship with the close family members, given the fact that you have a child with BAD?

R: Those on her father's side believe that it is the family spirits that are making my child have a mental illness. They allege that I normally don't go to their family meetings intended to appease those spirits, yet even my husband doesn't attend those meetings. They believe that because we don't attend their shrines, it is the reason why their daughter is suffering from mental illness. Even recently, when my husband was admitted here, one of the family members called and informed me that they had visited a traditional healer and that they had been informed that the family spirits related to my husband were the ones responsible for my child's mental illness. I asked them what way

forward the traditional healer had suggested, and she responded, saying that "the traditional healer said we should construct shrines at home". They actually insist that I am the trouble-causer since I don't listen to what they say. I reminded them that I cannot leave the hospital because by the time I brought the child for treatment, she was badly off, she was undressing, throwing stones, and speaking uncoordinated words. So, I failed to agree with their decision because they wanted us to just keep the girl home, and that she would get a fine. So, we have misunderstandings with them because of that.

I: How about your relationship with the community members?

R: That has been fine, I relate well with them. Most often, they were very supportive, they encouraged me not to listen to the family members and bring the child to a mental health facility.

I: Have you ever been discriminated against or stigmatized because of having a child with BAD?

R: I have not experienced that. The issue is that where we stay is a bit isolated, we meet other people less often. So, in most cases, we meet at functions such as burials, but another thing is that the whole village is occupied by relatives/family members from one ancestry. The people in the village are in one way or another related.

I: I would like to know your experience with your caregiving roles for that adolescent daily.

R: What I can say is that nowadays I stay at home because of this child. I used to work in the market, I used to spend a lot of time in the garden, but nowadays that is no longer possible. In fact, at first, I had a very bad experience because I remember I spent almost a full month crying and cursing about my child's mental illness. But with time, I got used to the situation, and now she eats and drinks a lot.

I: Sometimes, some people have challenges with finances. What is your take on that?

R: As I had said earlier, I have a challenge with the finances, sometimes I even fail to buy the prescribed medications. I no longer go to the market to vend things, my husband, who would help me in such times, got fractures and is now on crutches. So, the situation is not very easy, Sometimes, I have to call my brothers and sisters for financial support, even though one of my children is not at school right now because of money; he was in senior six class

I: You have talked about the time factor, how about transportation to the health facility, what is your take on that?

R On the transport issue, I understand it well that I have used 56,000 shillings, which is when I moved with her to the health facility. Sometimes I am forced to leave her at home because of a lack of enough transport money. Sometimes, as you are planning for the return date of the child, the father's return date is also due, so you find yourself in a fix. I left it to God to take charge.

I: In your view, what kind of support should be given to caregivers of adolescents with BAD?

R: To me, the main challenge is with transport and other daily items for use, so I think if we can be given enough medications for like two or three months, it can really help us a lot.

I: So, what duration do they normally give you here?

R: They normally give us one month, right now some of the medications they gave me are already finished, yet my return date is 6th August.

I: How about the caregivers also receiving counseling? What are your thoughts on that?

R: Now, who will counsel them

R: You see, this is the medication that got finished (referring to the empty packet of medicine)

I: Ok, this medication is for the side effects. We shall inquire at the dispensing window to see if they have this medication

R: Ok,

I: Now, sometimes when you look at the age mates of your child, how do you feel?

R: The truth is, whenever I see children in the age bracket of my daughter having mental illness, I feel very bad. Onetime I saw one here who had come from school but was very mentally unstable, I really felt very bad. Whenever I see a child with a mental illness at that age, I feel very bad because such a child has lost a lot. For example, my child was in primary seven, but right now I don't know if she will be able to sit for exams or not. Truthfully, it is a very bad experience. It makes me think about the various challenges that such a child is headed for in life.

I: In your own thinking, what do you think is the cause of your child's mental health problem?

R: I failed to understand that, however, sometimes I suspect that maybe it is due to the problems with the bones that she suffered earlier, or the seizures she had while still young. So, I fail to understand the exact cause

I: So has she ever had a second episode of the seizure since the first one?

R: She has never experienced a second episode. When she got the seizures, we brought her to a medical facility of Mutooro in Lukaya for treatment. When we realized that she had become lame, my father-in-law was a doctor, and he instructed us to take the child to Kampala for treatment. When we arrived in Kampala, the child was put in a room with many playing materials, the doctor their asked her many questions regarding the items inside that room and she responded correctly to all the questions. The doctor informed us that our child was fine, she didn't have any problems, but informed us that she only needed to do physiotherapy to regain functionality. In fact, by the time she broke down, she was using both hands equally, but right now she cannot use that hand; it is weak right now.

I: What do you say about the psychotropic medication that you are using right now?

R: The medicine is very strong; if you don't provide her with enough feed, she gets very weak. And also like I had explained, I got challenges with the medication when she started drooling saliva excessively to the extent that I had to come back to the health facility. Sometimes she becomes very weak after taking the medication.

I: Let us now look at the future. When you think about her future life, what comes to your mind?

R: My biggest worry is about her getting better; if she fails to overcome the mental health problems, she may find difficulties in life because she is already lame. If she doesn't recover, she will not go back to school. Before the occurrence of the mental illness, I was pretty sure that despite her being lame, she would study successfully. Right now, my biggest worry is that if she fails to recover, how will she survive? So, her future may be very challenging in case she fails to recover

I: Could you give me an example of a very challenging situation that you have had to endure while caring for your child?

R: The most challenging situation was my encounter with my husband's family members, who wanted to force me to seek treatment from the traditional healers, yet during that time, my husband was also down with nursing fractures.

I: What exactly did they want?

R: They wanted me to organize a function at home to appease the family spirits because they believed that that was the main cause of the mental illness. They had even set a condition that if I wasn't okay with doing that, I should pack my belongings and go and leave their child behind. I remember one time, I almost left, but somehow, I persisted and refused to leave.

I: So, what recommendations do you give to caregivers of children with Bipolar affective disorder?

R: What I can tell the caregivers is to always ensure that they seek treatment from the health facilities. I am not sure that a traditional healer can heal a child with mental illness who is undressing, abusing people, or even fighting. You see, even my child used to abuse, fight people, and even undress herself in public, but right now she doesn't do that because I brought her to the health facility. So, for me, the advice is hospital first.

Right now, my main request is about the availability of medications. If they can provide us with medications for longer periods, it will really help us a lot than giving us drugs for only two or three weeks, because it becomes expensive if you are coming from very far, like some of us. In fact, sometimes you have to contemplate where to go for medications because you are coming from very far

I: Apart from that, what else, how should they treat/handle such children?

R: They need to be very friendly to them because at first, I used to handle my daughter aggressively, a factor that would make her deteriorate further. So, I had to change my ways, which made her become my friend, and she has greatly improved

I: Do you sometimes worry a lot about leaving her alone at home?

R: Yes, that is very true. At first, I used to fear leaving her alone at home because I used to think that she would get lost/wander away

I: What makes you fear leaving her at home alone?

R: With these irresponsible men in the village, I fear to leaving her home alone, they can rape her, whom will you ask if such happens? But right because her mental state has improved, you can instruct her to remain in the house, and she will surely stay there.

I: Do you sometimes worry that she might harm her siblings?

R: That has actually ever happened to me, she locked her six siblings in the house and began beating them up badly, and even one of her siblings got a fracture as a result of being beaten. What helped was that they all gathered in one corner and shielded the young sibling, who was only two years old. She was accusing them of taking her photos without her permission, she thought that they were taking her photos to spread them throughout the country to show that she is mentally sick. From that time, I hid the smartphone from them until she got better mentally. Whenever she sees someone with a smartphone, she thinks that they are taking her photos and that they want to spread information about her that she is mentally ill. Another thing that helped is that I came back home early and rescued them, otherwise, she would have locked up the house and been busy punishing them without any hesitation. So, I had to always be around, I also had to sit her siblings down and explain to them about their sister's condition.

I: Okay, do you have any other things that you would wish to talk about regarding your experience while looking after that child?

R: I don't have much, however, the caregivers of these children should also endeavor to always honor the appointment dates given for their children to benefit from the treatment.

I: Sometimes, some people engage in prayers as a form of treatment. What is your take on that?

R: I failed to use that approach; I remember one time while here, one of the caretakers advised me to go to church and pray despite being a Muslim. However, I failed to do it until now

I: When I talk about prayers, I don't only refer to churches but also to sheiks praying for the child?

R: We tried that. I have an uncle who is a sheik and normally prays for people. Before coming here, we invited him to our home and he prayed for two days, but there was no change in the child's status.

I: Thank you so much, do you have any other questions

R: No, I don't have, thank you also for inviting us here

I: I beg we end the interview here

A QUALITATIVE INTERVIEW ON THE LIVED EXPERIENCE OF CAREGIVER 3 (C3) OF AN ADOLESCENT WITH BIPOLAR DISORDER

Interviewer - I

Respondent - R

I: My name is xx, I am a student at Mbarara University of Science and Technology and right now I am studying and I am doing a Masters in Mental Health Nursing, as I have explained to you, I am going to be asking you questions about your lived experience as a caretaker of an adolescent with bipolar disorder. This kind of mental illness manifests in an adolescent; sometimes it may manifest in the form of so much depression, or having an elevated mood, or being irritable more than what is considered to be normal. Before we go further, I would like you to introduce yourself, to tell me your name, your age, gender, your religion, and your marital status.

R: My name is C3, I am a Muslim, I am 57 years old, I am a female, I am not married, we separated, I stopped in primary seven - P.7

I: You stopped in P7, what is your relationship with this child?

R: My lastborn.

I: Your lastborn, and how long have you stayed with her?

R: I have stayed with her for about 5 years, because she grew up with her father.

I: You have stayed with her for about 5 years.

R: Yes

I: Ok, how many children do you have? She is the last one.

R: I gave birth to 8 children, I now have 6 children, 2 died

I: Sorry, thank you so much for introducing yourself and telling me some of your personal information. You as a caregiver of this adolescent with this kind of mental illness which manifests in two ways, as I have said, sometimes manifests in form of depression, or having elevated mood or being irritable, hearing from you may help us to understand

very well what it means to provide care to an adolescent with bipolar disorder. On that note, I would like you to tell me your experience as you take care of that adolescent.

R: The situation is not easy because whenever I look at her when she is depressed or she has refuses to eat, I find it difficult to handle. When she starts moving from one place to another, and I see that she is not in the right mood, again, I find it very difficult. There are times when she keeps quiet, it started even before I got to know that it was a mental illness. I started getting to know that it was a mental illness when she had the episode, she started wandering from one place to another, later I started seeing her picking things from the dustbin, my heart became depressed because my siblings had ever gotten that kind of illness. I became worried, and I said Let me take her to the hospital so that she gets treatment, That’s how I brought her here. They started giving her medicine, later she got pregnant, and I brought her here when she was pregnant. The illness did not come back while she was pregnant; she had the pregnancy until she gave birth. After giving birth, she refused to take medicine for her mental illness; she only took the medicine they gave to her after giving birth, and she stopped taking medicine for her mental illness. And she was well. When the child was 5 months old, I saw that the illness had come back, I started seeing the previous behaviours coming back, she started being talkative. She was doing what she was not doing before, not wanting to eat, walking from one place to another, talkative, I saw that even the eyes had started changing, the mouth started appearing like it had curved in a bit. I did not wait for more symptoms; I said Let me take her back to the hospital. When she has that illness, she usually does not want to talk to me, she is usually sad, and she hates me so much. I talked to my neighbour, I requested my neighbour to help me with my daughter, because she listens to what the neighbour says. The neighbour counselled her, talking to her, and she told her that she will be careless to her child and fail to breastfeed the child, and she asked her Do you love your child? She replied Yes, I love my child, and she told her Then listen to what your mother says, accept her to take you to the hospital, She accepted. I brought her to the hospital, and she was given the medicine. When she received the medicine, she went back home and took the medicine. The illness has not yet returned, and she has stabilized now. However, I am always on her case and telling her to come to the hospital every month they have told her to come to the hospital. I always remind her that you see now you are

doing well and you have stabilised, if you do not go to the hospital, then you will relapse and you will get a problem. Sometimes when she sees that she is doing well, she does not care to come back to the hospital, I insist on her, and I ask her if she took the medicine. She responds yes, I take the medicine, and I always emphasize that she has to take the medicine. I usually see it in her eyes when she is about to relapse, there is a way eyes change, I tell her I have observed that your eyes have changed, you have to go to the hospital to get the medicine or take your medicine if you have them, she says they wrote for me this medicine, I am going to buy it, I am not going to go back to the hospital. I tell her you have to go back to the hospital each month they tell you, until when they tell you to reduce on the number of times you come to the hospital or do not come back, she says ok, she has missed coming like past 2 months, after those 2 months, this month she came, she was given medicine. Sometimes she does not get medicine because it is out of stock. I tell her to buy it, but she had not bought it. She bought it yesterday because it is expensive, yet sometimes there is no money to buy it. I tell her any money you get, just buy tablets you can afford with the money you have at that time, and keep taking the medicine, and that is what she does. I no longer wait for her to relapse because I now know, I learnt how the episode begins, I see her and I tell her if she reduced the dose by herself, she should not reduce but take it as it was given by health workers. Do not be lazy to take medicine, I tell her that health workers will reach appoint and tell her to stop taking the medicine, if they do not tell her that then she should continue taking it because in case she doesn't, she will relapse. I usually escort her whenever she does not want to come back for a review. When she comes, she finds other patients. I tell her Do you see those patients? Haven't you heard that the health workers have said that they have stopped taking medicine? Even you in case you do not take that medicine, you will also be like them, she becomes concerned that she has to take her medicine. When she comes here alone, she comes back home and tells me those she found here, what they were doing, whether they beat their mother or father, whether they abused so and so, I also tell her that it will reach a time when she is like that if she does not take medicine, I tell her that she is doing well, not like them. I tell her that health workers will reach a point and tell her to stop taking it, or to reduce the dose, if she has been swallowing 2 tablets or 3 tablets, or even not taking it, but she should not stop taking the medicine on her own, I

am always telling it to her, she has now told me that she bought the drugs yesterday and took them. She told me it made her weak, I told her, even if it makes you weak, just take it, because she does not take too many tablets.

I: Ok, thank you so much for elaborating on those issues, you know, that's where you talked about pregnancy, and you said the mental illness came back after she gave birth.

R: After she gave birth, when the child was 5 months old, that is when the mental illness came back.

I: I would like to know, at what age did she conceive? Or during pregnancy, she was already married?

R: She became pregnant at 18 years old, and right now she is 19 years old. She has just given birth, the child is now 10 months old, she had not gotten married at that time because she went in marriage in January, the man who is the father of the child took her, isn’t this July? she has been there for 7 months, but for the medicine, it’s not that she takes them every day because sometimes she misses taking the medicine.

I: Why does she miss taking it?

R: I tell her but sometimes when she comes her, she does not get the medicine, sometimes it is not in stock, they tell her medicine is to be bought, go and buy it, when she comes, sometimes there is no money, she has to first get the money to buy it, remember when she relapses, she does not care. I tell her she has to buy the drugs and take them. Sometimes, when I have money, I give it to her and tell her to go and buy them. I just keep on telling her so that she buys drugs to take, because even when she has money, she cannot fail to buy herself something to eat and leaves out buying medicine because she sees herself that she is doing well. I get worried that she may relapse severely, yet it came slowly by slowly she had not reached a point of being too bad, only that it started before I got to know, she would keep quiet without talking, or not eating food like for a week, I did not know that it is this illness, and sometimes, I would see her moving from one place to another, that is when I said this may be mental illness.

I: Thank you so much for elaborating on that. I would like to know because you have said you have spent about 5 years with her. At first, she was with her father

R: Even that illness coming back, she had gone to her father’s home, I do not know what happened between them but her father beat her, so when she came, she was on taxi, the

taxi people called me and they told me that they suspected that she had become mentally sick, but they said they will not let her go out of the taxi before I reached there so that I receive her, that is what they did

I: Where does her father stay?

R: Her father stays in Kampala, that is what they did, they waited for me, I went, picked her up and brought her here, she was talkative, saying I am not mentally sick, they are saying I am mentally sick like that, that is when she relapsed

I: Relapsing the second time

R: Yes, that is when she relapsed, I told her to bring her to the hospital, she refused, she was just saying she is not mentally sick, and nothing showed that she was mentally sick, when she relapses, she usually speaks so much in English, I now observe it when she has relapsed, and when we reached the hospital, she was just speaking only in English, I first took her the other side, later they sent me here, but when she relapses you may not know it if you have not been there with her for some time, you cannot know that she is mentally sick

I: I would like you to tell me, when you were staying with her before the illness came in, how was your relationship with her, how were you treating each other, how was she treating herself?

R: She was behaving like any other child, how they behave at home, she had no problem, but when the illness came in, then she started behaving like that

I: When the illness started, what did you first think as a parent?

R: When it started, it did not start as you see these ones who are aggressive and violent, it started when she was keeping quiet, not speaking for a week, someone gave me some medicine, she took it, and she recovered, she started speaking again, that cleared

I: The medicine they gave you was this modern one?

R It was traditional, the person told me that maybe your child was given a spiritual curse, “ekitambo” at school. But there is my son who gave medicine to a child, she took it and recovered, since he is your friend, go and tell him to give you some medicine. So, he gave me some medicine and told me to give it to her to drink, she would speak, and when I brought it, she took it and she recovered, then it cleared. It takes some time without coming back, then the next thing was she started with not eating food, I said For, a week,

this person is not eating food, what is happening? Later, after a week, she resumed eating little by little, and when she recovers, she usually has a very high appetite, eating a lot of food. I saw that but left it the way it was, then she recovered. I started knowing that this is a mental illness when she started being restless, sitting from one place to another, but I had prayed to God because I had seen my siblings with the same illness, and I would pray to God to help me, not to give me children with this illness

I: Were they your siblings?

R: Yes, I have my brother and my sister, my sister has a different mother but the same father, my brother and I shared the mother; they had that illness, they used to get medicine from here, my brother died, but my sister is still receiving the medicine. I do not know whether she gets it from here or somewhere else because currently, I do not hear much about her. Then I said this child may get this illness, let me take her to the hospital, people told me to take her to the hospital, others to traditional healers. I remembered when my brother got that illness, they did everything, and it failed. My sister, the one I follow, told them, 'Do everything. when you get tired, you will give him to me and I will take him to the hospital.' They did whatever they did, it failed, then my sister brought him here. When he brought him here, health workers took him to Butabika hospital because the illness was severe. He started taking medicine, he came back, and he started getting medicine from here until he died. I also said I would not bother going to traditional healers, let me go to the hospital, then I brought her here, and she started taking medicine

I: When you brought her here and she started taking medicine, the first time you brought her, how did the health workers treat you? how did you see the situation?

R When I first brought her here, we started from the other side, where general patients are received, health workers called me, after seeing her, they talked to her, then they called me like the way you have called me and they took me in the clinical room and they told me that I should not do any other thing, they asked me if I have ever seen a person who kills him or herself, or I that I have ever heard that a person killed themselves, they told me it's this illness. They also asked me whether I have ever heard a person coming from nowhere and kills a person, they told me it is this mental illness, then they told me to take her to the mental clinic to start medicine, that is how I brought her here, I did not find any

challenge the other side, I just came here. When I reached here, we found a youth who was tied with ropes, he was talkative, she said, you have brought me here to be among murder criminals, I am not a murder criminal, let us even go back, she refused, that day we went back home without receiving treatment, I tried to talk to her but she was not listening, she was not listening to me

I: She refused

R: She refused to stay here after seeing the other one tied on ropes, and she asked, 'Do you see me as a sinner?' Why have you brought me here? You told me you were taking me to the hospital, but you have brought me this side. I do not like this place, and I will not be here. We went back with her, we went back as I was telling her that she would have received the medicine, she refused when we reached home, we were there, she recovered and stayed well. When she relapsed, she started being restless, sitting from one place to another, going to the neighbours' homes, refusing to eat food. When she relapses, she does not want to eat food, as I have already told you. I went to the neighbour who told her to allow me take her to the hospital and do whatever they tell her to do. That is when I brought her here at the hospital and she started taking medicine, like what I have told you, she started taking it because she was staying with me, I would tell her to take the medicine, every night, because there is one type they told her to take it only at night. She then started stabilizing, even during pregnancy, she would take it, she used to come here and the health workers used to give it to her and they changed the type of drugs she was taking after they got to know that she was pregnant - it was different from the ones she was taking before she was pregnant. She took her medicine until she gave birth, after giving birth, she started refusing to take it

I: There is where you said that you feared, you used to pray to God not to give you children who are mentally sick like your siblings

R: Yes

I: Then she got the illness, you confirmed that she has a mental illness. How was the situation after confirming that she had a mental illness?

R: I got worried and felt depressed so much in my heart because I was not expecting it, all my children had grown up, and no one had that illness, even this one had grown up. I took it that since her father beat her, she became disturbed, and I thought that she would

be fine, because it did not come from the air. When she refused to take medicine, she was doing well for some time. After the child's delivery, I thought it might go and not come back, but later I saw that she had relapsed, not eating well, and her eyes started turning. Then I told her, let us go back to the hospital and they will add you more medicine, but she was not caring, she kept saying I am not sick, why are you calling me sick? And when she is sick, she is usually irritable and hates me so much

I: In that situation, when she hates you yet she is your daughter, how can you explain that situation? How do you feel?

R: I find it hard and I feel bad in my heart, but later I say I have nothing to do, maybe this is due to the illness, because when she is stable, our relationship is good, but when she relapses, she hates me so much. This time, even when my neighbour talked to her, she refused to come and get the medicine. Later, she again talked to her and she agreed to take the medicine. The neighbour asked her; Don't you love your child? She said yes, I love my child, then she told her to listen to what I was saying. You go to the hospital so that you can get the medicine, but you will be careless with your child if you relapse, and your child will suffer. Then she comes to the hospital, whenever she gets the illness, there is usually someone she loves so much, but she usually hates me. When that person tells her something, she listens, so I am now used to it, and I know that is how the illness comes, and I bear with it, knowing that she does love me, but it is because of the illness.

I: I would like you to continue elaborating about what you experienced when you were taking care of her daily, since she was sick, she needed medication

R: The situation was not good. I am supposed to buy those drugs, because sometimes when we come here, they give us some drugs and they tell us that the other drugs are out of stock. So, we have to buy the drugs, reaching out to buy them, they say each tablet is 500 shs, yet sometimes I do not have money. I have to try to look for money so that we can buy those drugs so that she can take them to stabilize, because whenever I am there and I see her restless and moving from one place to another, I feel bad as a parent. If we do not buy it today, we try and buy a little so that she keeps swallowing that until we get more money to buy more, until her review date comes and she comes back to the hospital, if she gets some, she takes that one

I: Apart from money and drugs sometimes not being there, is there anything else? And what about side effects? How does medicine treat her, and when it treats her like that, how would you find it as a parent?

R: I feel pain in my heart, but I have to be strong in this situation because I have nothing to do

I: How was it treating her?

R: It makes her weak, she sleeps too much, and explained to the health workers and she told them that the drugs were not treating her well, then they changed the type of drugs she was taking to this one she is using currently. She no longer sleeps as much as she used to, but the one she bought now, she told me it makes her weak.

I: What about providing for her other necessities, like food or clothes? Is there any challenge you have experienced?

R: The challenge is there because now we are staying in town, and we have to look for food to eat, we buy some food and she eats, yes, because when she relapses and then recovers, she eats and drinks a lot, let alone wanting to eat good food only, so I find it a big challenge

I: Taking you back to what you said, about the issue of marriage, when she told you she is going to get married, how did you feel?

R: I feared that she might go to the husband's home and she relapses, but I said, since I have gotten the chance, that she will be staying near me, I can get to know her, I can see her. The advantage I got is that the husband who impregnated her took her, accepted to take her and he also used to take care of her during pregnancy, she gave birth and he accepted to take her, so I thank God for that because some mentally unwell people get impregnated by men and are left there. I kept praying to God that the illness doesn't return, yes, that is why I am always on her case, I keep telling her to take medicine because when she keeps on taking medicine, she may not relapse, and if she stops taking medicine, she may relapse. Even this time when she relapsed, I saw it quickly in the early stages before she fully relapsed, and I told her to go to the hospital, because I saw the mouth curved, the eyes changing, but ever since she went to stay with a man, the illness has never returned. The problem with her illness is that it is usually due to thinking too much. There are times when she thinks too much, and I see her thinking too much, and I ask her, 'What

are you thinking about?' Why are you quiet? She does not speak but overthinks, but when she is not overthinking, she is usually fine. Remember, on the other side, she is usually busy doing different kinds of work at her home, but at my home, she used to be too busy; everything finds her there. On the other side, she is usually busy; she does not have time to sleep or sit there. Maybe when she takes the medicine, it makes her weak and she sleeps, now she no longer thinks too much

I: Does the husband know that she has this kind of mental illness?

R: He does not know. We told him she has a persistent headache, and they told her to go to the hospital every month

I called him on phone and I told him that her illness is bad, when she gets that headache, it treats her badly, and I told him to make sure she goes to the hospital every month, whenever they tell her to go back, he knows that she has persistent headache and he also buys for her medicine whenever he gets money and gives it to her

I: You talked about your relationship with her, and you said that when she relapses, the relationship is not good

R: Yes, she hated me so much

I: What about when she stabilizes? How is your relationship with her?

R: The relationship is good, as the daughter is with her mother, but when she relapses, she hates me, there is nothing I can tell her, and she listens, she hates me so much

I: What about your relationship with relatives, because sometimes when some people see that you have a child with that kind of illness, they segregate you and all that? How is your relationship with your relatives?

R: The relationship is there, it's not bad because we have had such illnesses so many times at home, so they all feel bad for me, everyone is sympathetic. There was a time when we came here, there is a pastor, because she is born again, she has her pastor who pastors her, he is the one who helped me at first to bring her here. I had forgotten to talk about it, he helped me, I called him and I told him that Sarah is sick but help me, they came to see her, I told him that he does not love me and she does not listen to whatever I tell her, and he told me that what we should do now is to take her to the hospital. He counselled her and he said that when I fail and she has refused, call me on the phone, I will come and take her to the hospital. So, when she refused, I called him on phone, the pastor came on

a motor bike, because that day I even did not have money, as you know when you have a patient, sometimes you reach a point when you do not have money, pastor brought a motor bike, we put her on and we came here. She was here with the pastor; the pastor was the one following her, and she hated me. She kept complaining and inquiring why I had brought them to the hospital, she would tell the pastor that she was not sick but well, we came with the pastor, the pastor took her around, as you know, things of the hospital

I: Is the pastor a relative or just a pastor?

R: Pastor at their church

I: Ok

R: He is the one who persuaded her to come here and he brought her here, now when she relapsed the second time and she had to come back to the hospital, she refused, the other pastor had a problem, he had lost a neighbour and he told me that he will not be able to come and take us to the hospital but told us to go but if she refuses, he may get some time and he told me to call him. We came to the hospital, when we reached here, she said I am not going with you unless my sister Dorah's mother comes, she has lived with her. She said that if Dorah's mother is not there then she will not go with me, I will not go with you, she said she is fine, then I called my sister she came, she persuaded her on phone to stay at the hospital until when she comes and she told her that she was on her way to the hospital, but she kept saying that she is not going to go to the health workers because she is fine. My sister is also born again, they go to the same church, she came here, they went to the health workers, health workers gave her medicine, even on that day there was no medicine here, but they told us to go the other side and it was there, there is a health worker helped us to get the medicine, he gave it to us and we went back home

I: So, in brief, even other people do not segregate you or discriminate against you because you have a daughter with mental illness

R: Yes, that is what I wanted to show you, my sister came and we took her around the hospital and she even escorted us and even the pastor came and picked us, he said if you have finished call me, I will come and pick you, we called him, he came and took us back home

I: Have you ever experienced a situation that is challenging regarding the relationship between you and your relatives or the people in the community where you stay concerning your daughter?

R: It has not been there; there have been some disagreements with my neighbours, but concerning her, they have been loving her. We may not be relating to the neighbour, our personal issues, but they are not associated with her

I: What about health workers? When you brought her, what did you say about it?

R: There are times when they are rude, and I feel like, oh my God! the first time we came was the hardest because we came late, we first went to the general clinic as I have told you, and we reached here late, and the health workers asked us, what were you doing there? It's now 2 pm, why didn't you come early? We are closing, you are just coming, I just kept quiet, you know I do not quarrel with health workers. I wondered what if sickness had come, we came, reached the other side, remember we first sat on chairs in a line, we came this side, they did not ask why we came late. In my heart, I thought that this illness just come at any time, it does not have time, like you spend a night sick, then you go to the hospital, now we have reached here, instead of helping us, they are just quarrelling. I found it hard on that day, and the patient I brought was not cooperative. at that time, he also got the gap because health workers did not pay attention to us, and she said Why should we be here? I am not a criminal, so why should I be here? She had seen the one on ropes, yet if they attended to us immediately, she would not have seen the other one on ropes, like how we did the other side. we waited, the health worker explained to us very well, and they told me to come this way. When I arrived, they were already arguing. She also had a gap in her care, and even the health workers said we were too late. They suggested going home instead. She left, and I decided to go too, but I talked to her as we left. She was upset and told me not to speak to her. While she was quiet before, she changed completely here. We went home without any treatment and didn't return until she relapsed. I had initially brought her to see her pastor for counseling, but when we tried to return a second time, she refused to come back. Then I called the pastor to come and he brought us, that is when she started treatment, but if the health worker attended to us immediately, not shouting at us, not quarrelling that we have come late, they would have worked on her and we go, that is where I found some

difficulties but the rest of the following visits to the hospital it has been fine except the challenge of not getting medicine. I reached here and they told me no medicine, they gave me one type of drug and they told me to buy another type, yet I didn't have money, therefore, I only took what was available.

I'm still on the issue of health workers. did they teach you about the condition your daughter has? And they explained to you?

R: Here, I do not remember them teaching me, apart from asking me questions like you have asked me, and I tell them

I: Like may be when the health worker finishes asking you all the questions, and then he/she explains to you that now your daughter is suffering from this and that

R: No, they have never told me that, health workers the other side are the ones who called me into the clinical room, asked me questions, and I told you as I have told you, that illness started like this and that but here, they have never told me this and that, may be encouraging her to take medicine

I: But they have never explained to you that this is the kind of illness, do this, avoid this

R: No, they have never told me, maybe for her, she was told to stop thinking too much, even the other side, they told it to her, and here they told it to her too, to stop thinking too much. I also ask her what makes her think too much, I encourage her not to think too much; if she does not think too much, the illness does not come back. I also do not know what makes her think too much, and God helped her that she got a man though she has just gotten her, let me hope he will not change, if God helps us, but he does not put her on pressure to make her think too much, that can also help us

I: Thank you so much for explaining those issues now. Have you ever been segregated because of having this child who has this kind of mental illness?

R: No, I have not gotten that, because her illness is usually not strong enough, so that people do not get to know that she is mentally sick. She also got an opportunity that people love her, they sympathise with her, whoever she finds wants to talk to her, they talk to her well, and she is happy talking to them

I: In your mind, how do you think care takers of adolescents with the kind of mental illness like your daughter has should be helped?

R: Even now, if I find a caretaker of someone with this kind of illness, I tell them to quickly come to the hospital; they should not bother going the other side. That person, whether child or adult, I tell them to go to the hospital because I will show them the example of my child who has now stabilised, my brother and sister were taken to the hospital, and my sister has given birth to children. We are almost the same age, because our fathers gave birth to us in one year, but she has some children she gave birth to when she had a mental illness; the children are healthy, because for her it mostly used to come when she was pregnant. So, when I saw my siblings, I knew that even when this one comes and starts talking about medicine, she will be fine. I did not waste time going to traditional healers or church to pray, people used to tell me, because even at the church where she prays from, the pastor I have told you about told her, we have patients with such kind of illness, we pray for them but they are also on treatment, they go to the hospital and they get the medicine and they are doing well. I had also explained to him about my siblings, he said, even you, Sarah, you have to go to the hospital and get medicine. You will be fine, but do not bother refusing to go to the hospital. As I have told you, we brought her together with that pastor, and she started taking medicine. I talk to people and tell them that if the person gets a mental illness, do not bother spending money, just take the person to the hospital so that she starts taking medicine because it's free of charge. If you reach here, they write for you the medicine, if you have money you buy it, but when you go to traditional healers they just eat your money, because when we reached here, we found 3 siblings from Sembabule, they told us that in their family their father had a lot of cows, he sold all of them going to traditional healers, he had land and he sold it piece by piece to get money to take them to traditional healers but when he brought them to the hospital, they got some difference and they are there doing well and working. So, whenever you come here to the hospital, you get something to learn because when I arrived here, I found different kinds of patients and I go back asking myself what happened in the world. The time when my siblings became sick, they used to come to the hospital and they would get the medicine. There used not to be stockouts, it used to be there, and they were few, not many, and we came here, we had come to see her, she was admitted, and there were few patients. However, whenever I come here, I find so many patients, and I ask myself why: young children, nice-looking women, handsome and beautiful youth. There

was time I found a youth, he was a Seventh Day Adventist, he had a young wife, they had one child, they were coming from Kampala, their mother is born this side, he was brought here, the youth was looking good, he was very smart, the words he was speaking where hateful, he hated his mother, listening to only her wife - I felt sad, I sympathised with them. I left when he said he would not take the medicine; he said he is not a mad person, he is fine. I do not recall the work he was doing, but he was very talkative, I said it is not only in me, even others are here, but I said I do not know where the illness came from because sometimes back people with this illness used to be few, you find few, but now they are many. Even the son to my husband, the father of my older children, my daughter to that son, recently she also got mental illness recently, they called me to say that she has gotten mental illness - she is also called Sarah. I told him to take her to the modern hospital, not to spent money to go to traditional healers, the daughter was working somewhere in Chinese company. There was a man who requested her that he take her for treatment, but I thought they were taking her to modern hospitals, yet they took her to traditional healers who asked for one million shillings from him. She took a long time to stabilise, but she later stabilized. Remember that illness comes and then goes for some time, she recovers and then relapses. I kept quiet and I said they have spent all that money, but they will eventually end up going to a modern hospital, when she relapses

I: Thank you, now, how do you see the future of your daughter when you think about it sometime? the good thing she is now married, but when you see it, are there times when you get worried about what may come in the future?

R: I worry sometimes, I become worried because sometimes I say that what if I am not there, or that she may relapse when I am not there, how will she be able to survive? Right now, if she relapses when I am there, I can quickly bring her to the hospital, but if I am not there, I don’t know. I normally pray to God to heal her so that she can be well.

I: Do you sometimes get worried about things like work, how she will handle it?

R Yes, but now, the only thing is that when she takes medicine, she becomes weak, but she has been doing her work well, and how it is supposed to be done. One thing I pray to God is to help her heal. I have hope that she may heal, and the illness never comes back again. I get worried, the illness is bad it makes me worried so much, because when my siblings used to come to me when they relapsed, because they loved me, they would come, when

you are there, you just see them coming and when they come, they stay there for some days and they begin to relapse. I also have my other sibling who follows me, for her we took it that she got HIV/AIDS, she had 3 children, the first man she had died and when she was already infected, when she became infected, she reached a time when she became mentally sick, I was staying with her, I had even gotten for her a job, she had started working, she came from work when she had relapsed, she has already started taking medicine, because ART had just come at that time, she had just started. She also had a mental illness. She refused to eat, we took her in the car to the village at home, and she stayed there. Ever since she got the illness, she never ate anything or drank anything, only talkative, until she died

I: Sorry, can you give me an example of a situation that challenged you the most or is challenging you the most as you are taking care of your daughter?

R: The most challenging situation, is that when I see her there not eating, not drinking, the thing which depressed me the most and which compelled me to bring her to the hospital was seeing her restless, sitting from this place to the other, one time I saw her kicking a bottle in the road and I said ho! I think she has gotten mental illness and I got so much worried and I became weak, and I said God has punished me, I was not expecting this, and she never loved me, she would look at me in a hateful way, and I also get somehow annoyed, as a human being you also get annoyed. I felt annoyed seeing that she hates me, I feel like I have a big problem. That was the most hurtful thing. Seeing that my daughter had gotten mentally sick, I felt worried, and I said Let me pray to God

I: As we are about to conclude, can you explain to me the kind of illness your daughter is suffering from? Can you describe it? How do you understand it?

R: I can describe it that it is mental illness, and when it comes, it makes a person weak

I: Do you know the name of the mental illness she is suffering from? Apart from saying that it is a mental illness?

R: The name? I do not know, they say mental patients, so it is mental illness, I do not know its name

I: What about the drug she takes, do you know it?

R: I do not know it, and when they write it for me here, I go to the health worker, and I tell him, the challenge with that medicine is that most of them do not have it, its few clinics

which have them where I can buy it from, but I do not know the name of the drugs. I just take the medical form where they wrote it or the book, and I tell the health worker they have written for me this drug I want, if they have it, they sell it to me

I: It is good that you take it to the pharmacy rather than the clinic

R: I first went to the clinic, but they did not have it, and I went to the pharmacy; some pharmacies have it, others do not have it

I: It's true, ok, thank you so much

R: Now, what I am asking is if she stabilizes, can they tell her that she is going to stop taking that medicine, or will she not stop taking it

I: Now, it depends, sometimes she can stabilize, and the health workers say that lets reduce the dose, or she has been swallowing this number of tablets, now let us reduce to this, some of them tell them to first stop taking medicine as they observe. It depends on a person, if they have been consistently taking medicine well, health workers can reach a point and say, let us first try and see, let us reduce the dose and see

R The challenge we have is that the book she has been using for reviews got lost, she says the book got lost, we tried looking for it and could not find it, another one also first got lost but later I saw, they told us to bring it back if we see it, but when the book got lost, she came back with the old one, now it's the new book which got lost

I: The good thing is, even if she comes back with the old one, as long as it has the file number, they can look for the file because whatever they have written in that book, it is also written in the other file, so do not be worried about that

R: I thought that when the book gets lost, they may give her drugs, which are different from what she was taking

I: No, when she comes, they also need to review the file and see what is there, and they make it in the right order

R: I thought that they may stop her from taking it after she stabilises, because it takes some time when she has not relapsed, because now, ever since she gave birth, after 5 months, from that time, she has been fine and not relapsed

I: Ok thank you so much, I request we stop here, we can talk about the rest after stopping the recorder

A QUALITATIVE INTERVIEW ON THE LIVED EXPERIENCE OF CAREGIVER 4 (C4) OF AN ADOLESCENT WITH BIPOLAR DISORDER

Interviewer - I

Respondent - R

I: My name is xx, as I have explained to you before, and I am a student at Mbarara University of Science and Technology. I have come here to ask you some questions because you are a caregiver of an adolescent with bipolar disorder. I am going to be asking some questions, as I have already explained to you the information concerning this study. Before going further, I would like you to tell me your name, your age, your gender, and your religion. I will be telling you the rest. Let us begin from there

R: Let me first begin with gender, I am a female, my name is Joyce

I: J, who?

R: C4, I am 39 years old

I: What about religion?

R: Protestant

I: Ok, what is your marital status?

R: About marriage?

I: Yes

R: Elaborate for me, what do you mean?

I: Are you married, single, separated?

R: I am married

I: What is your highest level of education?

R: Senior 2

I: Senior 2

R: Yes

I: And you completed it?

R: Yes, as you know, some years back, it was not so much emphasized that the child goes to school, when the child starts growing, they say, aah**.....** If dating someone, let them go into marriage

I: What is your relationship with this child?

R: She is my daughter

I: Your biological daughter?

R: Yes

I: For how long have you stayed with her?

R: Ever since she was young

I: Ever since she was young

R: Yes, she goes to school and comes back home

I: How old is she?

R: She is fourteen years old

I: Ok, you as a caregiver of an adolescent with this kind of mental illness, hearing from you may help us to understand very well what it means to take care of an adolescent with this kind of mental illness called Bipolar Affective Disorder, on that note, I would like you tell me your experience as you take care of that adolescent.

R: That situation is not easy, it is a difficult one. It requires some patience because such a person most of the time does a lot of annoying things and does them by force and is irritable. So, if me as a care taker, I am not calm and not knowing that whatever this person is doing she is doing it out of her right state of mind, and I also apply the same energy she has, then I will fail to care for her. If I tell her this, she tells me that, and she sees that she is the right one, then I have to calm down and know that the person I am taking care of is in a situation that is not normal. So, after calming down, whether she quarrels with me or abuses me, I have to consider that she is not the one and just continue taking care of her. It will reach a point, and she stabilizes, and when she calms down, she will start asking me how she ended up in the hospital, and I will inform her that she was sick. If she continues to ask how and what made her sick, and also asks about the drugs, then realizes that she was out of her mind, and that maybe I reacted with a lot of anger. And, that is why I say that any caregiver or any parent of a child with such a condition has to calm down, despite going through a lot of challenges. For example, I do not know the number of my clothes she tore moreover in a short period, then I imagine those who spend a lot of time in an active episode, how much do they destroy? But real calming down is what you are supposed to do, so that you take care of a person so that she stabilizes.

I: Ok, thank you so much for explaining on those issues, which other situation have you experienced while taking care of her?

R: May be most of the time they usually refuse to eat food, I may bring something good to eat, but she may just touch on the food only once then she throws it on ground or throws it back to me, so it makes me feel disturbed too. She does not sleep, which makes me feel uncomfortable

I: Thank you so much, I would like to know, before the illness came in, I would like you to explain the relationship between you and your daughter, how was it? Before the illness came in?

R: Before the illness came in, she was well disciplined at home, at school and church. I do not even know how to explain it to you, because even at school, they used to give her gifts for performing well in academics. At their Born-again church, she holds the position of an usher. She was a calm person and when we saw that her words were getting many, we feared it so much because she is a calm person, she is a kind of a careful person and it disturbed us to know very early that she had gotten a problem, words were too many, which was not there before.

I: Ok, the relationship with her, how was it?

R: She did not have any problem, she did not have any problem, no

I: What I forgot to ask you, how many children do you have?

R: I have them, but leave them, I will not say the number

I: Ok, how did those symptoms begin, and in starting, how did it treat you?

R: The truth is I did not know so much about her symptoms reason being that she was at school, where her clothes got lost. She had her clothes, which got lost; she had hung her knickers and a vest, and the book she had left in the desk also got lost. The book was the first one to get lost; she told us that it got lost, and we sent her some money to buy another book so that she could get notes from other fellow students to replace it. Immediately before completing this, a knicker and a vest also got lost, we wondered why - it disturbed us. After a week, they brought them back, and they put them under her desk

I: They put the knickers under her desk?

R: They brought them in a polythene bag, the other real book which got lost, a knicker, and a vest, and they put them under her desk. When she came, the truth is that that is what she

saw first, and again in touching those things, she just requested in the office for permission to first go back home. She came home with the items and was complaining that they had taken them when they were clean, but now, they brought them back dirty.

I: Did she tell the school administration about it?

R: She just requested permission, she just requested that she wanted to go back home, when she reached home, we welcomed her, but we saw her and could not figure it out, the way she was, we asked her, Are you sick? She said no, after telling her that, we saw her bringing out those things, we asked her what happened, she told us my belongings were stolen for this period, I had washed them clean, but on opening, they were as dirty as dust. We told her Why have you brought them? You should not have brought them. As parents, we got angry, we told her, you would have thrown them because you are not going to put them on again, you cannot put such dirty things on your body. Later in the morning, she started singing only religious songs, in which ever work she would do, religious songs, we wondered, she was preparing to go back to school. Then at night, she woke up at around 5 am, and she brought water put in a mopping cloth going to mop, I told her it is still night, she said she wants to mop in the morning because she wanted to go back to school, I told her, sleep again, it is still early in the morning.

I: At 5 pm

R: Yes, later we saw her getting a comb, started combing her hair, we asked her; What is it that has happened to you? After that, I talked to an older person (older than me), and I explained to her the condition of the child, and she also wondered, and said, her belongings, which got lost, may cause something not good to her. I asked her what I should do. And she told me; Do you see that situation, I replied yes, then she told me to take her to the hospital so that her condition stabilizes quickly, so that she goes back to school. Even traditional treatment works, but it takes more time to take away that thing that has come on her head. I further inquired about where to take her, then she told me to bring her to Masaka because she had a friend’s relative who was getting some medicine from there and had recovered. I advise you to take that child to the hospital, I replied ok, now by the time it was 2 pm -4 pm. Her eyes became red, she was not fearing anyone, eeehhh**....** my God! She had a lot of energy; the truth is that we were shocked and planned and agreed to bring her in the morning to the hospital.

I: When all that happened, seeing your daughter changing, how did you feel?

R: The truth is I cried, until when I lost all the tears, because in the community I would see some mentally retarded people, I would imagine that now my daughter is also like this - the one I had so much hope in is like this. I see mentally retarded people who did not stabilize, then I could not even want to compare, how it can be for my daughter. I was just crying, not understanding it, and I reached here when I could not understand myself. The health worker who saw me can tell you I came here I did not understand anything. I felt so bad, my whole body became weak, and I felt body pain; that situation was bad. You know if a child gets a fever, you can bring them medicine and know that they will get better, but with this illness, you don’t know if the child will get better.

I: When you reached here, did you find others? When you saw other sick people, when you saw them, what came into your mind?

R: When I reached here, I found other people here, but I saw them in their right state of mind, and I asked, even those they were like my daughter is now? They replied that where you have reached is the right place, it treats mentally sick people, all those people you see have come back to get their medicine, they stabilized, even yours will recover. It’s the health worker who first talked to me. She told me that your daughter will recover, since you have been quick to bring her, she will also stabilize quickly, and I felt encouraged. And when she was given medicine, the following day, she came out and asked me Where are we? I explained to her and she asked how we reached the hospital, she asked what happened to her and I gave her medicine, she asked this medicine you are giving me, what am I suffering from? What happened to me? I told her I will explain to you after you recover, not now. Mental illness is a hard illness to understand, unless when someone has a mentally ill person, that person is the one who can understand this experience, but if a person has never had a mentally ill person, they cannot understand it

I: In that situation, I would like to know your daily experience in your work of taking care of her? challenges, experience, that's good, and the bad ones

R: There is nothing good. I told you the challenge, you can try your level best to get what you see is good for her, say she used to love boiled eggs and chicken, when you bring them to her, she just throws them away. If you have given her tea, she mixes it all and

then pours it down. Of course, I feel annoyed as a parent, but I calm myself down. There is nothing good, no one should lie to you, it's not there

I: Apart from sometimes bringing her things you expect she to like, and she does not want them, what other challenge?

R: Challenges are many, sometimes in the community, I do not want people to know that my daughter has this kind of illness, because I know that if anyone gets to know about it, many people say a lot of things - they bought evil spirits, they looked for wealth, they dedicated her to evil spirits, stupid things like that. It is hard to convince people that this situation just came on its own; no one will believe you. Everyone says whatever they want to say; the challenge is great, but the illness is not easy. When she starts talking or quarrelling or abusing, she does not know that this is my parent or this is so and so, she abuses all of you, and I say Oh my God! That situation is not good

I: There is where you said about people saying may be there are certain things you did, apart from that, what other things do you think people can talk about regarding her?

R: What can they say? Like what now? You may also know may be this, or that, like what now?

I: I am getting the story from you, these questions I am asking, I even ask other people and they tell me their stories also

R: Some may say that in their family, there is one who had such a kind of illness, sometimes you never saw that person, a situation like that

I: Sometimes, when some people are taking care of these children, they face a challenge when it comes to finances. How has it been on your side?

R: God has helped us, if we had first gone to traditional healers, those are the people who mostly take money. They tell you bring this, buy this, bring that, by the time you discover where you will get the right treatment, the truth is that by that time, you no longer have money

I: But on what you said earlier, there is a challenge

R: Big challenge, in that if a person has gotten the illness, the caregivers should be wise enough to first run to the hospital. Whether they have told them about traditional treatment, they can research it later, but when their person is back to their right state of mind

I: For some, when they reach here and they start the child on medication, this medicine comes with side effects. What do you say about the side effects of the drugs she has been given?

R: I have not experienced any problem with the medicine, maybe in the beginning, they gave her 4 types of medicine, 4 categories, 2 were to be taken during the day, 2 at night. She could sleep too much, time for lunch, you just have to wake her up to eat, and sometimes she fails to eat, then at night she could sleep, and it's me to wake her up to drink water or juice. I brought that complaint here, they told me she should reduce the dose, when they reduced the dose, it also changed, so I do not have any challenge in that, apart from the beginning, before discovering the right dose she can manage

I: For some, the challenge is in time. We have seen some people come here, they want to leave the patient here so that they can go and do some work. They do not have time. What do you say about time?

R: About time, when a person gets a sick person, and they still want to take care of the patient and at the same time run the work they left, the truth is it fails, either of the two. You have to first put on hold one of them, and work is not more important than a human being

I: So, for you, what did you put on hold?

R: Work can first come on hold

I: What kind of work do you do?

R: Work?

I: Yes

R: I have my job, it's there, I have it, it's there, I first put it on hold, she first stabilizes, then I open again

I: I may want to support you

R: You stay far, you said you are from Mbarara

I: You never know, I may end up coming this side,

R: No, you are far

I: So, how has the issue of time treated you, has it affected you in any way, maybe some have to die because you have to take care of her?

R: No, it will not get destroyed because if it was that I left some things in the fridge, maybe I can say they may go bad, but if they were not there, I can still find them

I: Ok, maybe to say that these days I have not been there, I have made some losses

R: When it comes to a child, maybe because, for me, I first struggled to conceive, there is nothing precious I know as a child

I: You know, I asked certain things and you never told me. I asked about the number of children, but you never told me

R: The reason why I do not want to tell you the number of children is that I first struggled with conceiving

I: Ok, I would like to know how your relationship with your daughter has been.

R: My relationship with her is good, my daughter does not have any problem, she is well-behaved, and well-mannered. She is the kind of person you give something and they tell you it will be enough yet for you, yet you see that it will not be enough for her, but she tells you that it will be enough. She is a kind of person who can use something and does not tell you that it's used up, but it’s you who thinks and says, does she still have the things I gave her? You give her other things, then she says I had already used what you gave me some time back, but I was fearing to tell you, she has no problem

I: From what you have explained, you show that your relationship with her has been very good, but what I would like to know is that when she was relapsing, how was the relationship, did it remain good, and how was it? That is what I want you to explain to me

R: It wasn’t good at all, she even stopped being disciplined, even when I told her my daughter used to be disciplined, she looks at me angrily, and I say, God! is this my daughter? She changes a lot, but patience is the best in that situation, when your person has gotten that illness, it's patience, that’s when you can take care of her

I: I would like you to talk about your relationship with your relatives, how has it been?

R: The relationship between relatives, on my side or my daughter’s side?

I: All of them because they have all become yours, all of them have become one

R: There are things that separate us

I: Ok, if there are those things that separate you, you will explain them

R: There are those things which separate, let me tell you, she can become sick, and I do not even bother telling them

I: Your side or the daughter’s side

R: Ask me why?

I: Why?

R: You can prosper and they remain poor, and the truth is when you tell them that issues, they say, can you ask yourself or wonder about that? They deserved it, they bought some evil spirits. Why is it that we are not prospering like them? That is why I keep quiet, God helps me, I walk in my direction, but there are people, as I have told you, elderly people. There are people I know, and the truth is that that person is on my side and is always there for me. I am not so old in age, but I can make friends with elderly people, older than me, why? Because they can advise me and tell me that you see that thing, you can do it like this, they are the ones who advised us, and they told us, you see, whether this illness is traditional, but first go for modern treatment. Modern treatment will bring her back quickly, you will see the rest after she stabilizes. They showed us that traditional treatment also treats, but it takes a long period for the child to stabilize or an adult to stabilize. For some, their brains may constantly remain in that state, but when you go for modern treatment, they come back quickly to their normal state of mind, and I saw that telling relatives, I just told my friend who is mature in the brain, and I got something good from it

I: So, what you are explaining shows that even before the illness came in, the relationship between relatives was not there

R: It’s not so much that we related very well, and usually if you are not on the same level financially, it separates relatives

I: And when the daughter became mentally sick, then you just said I do not need to tell them anything about it

R: And they do not know about it

I: Ok, what about their brother, didn’t he tell them?

R: I do not know about him, whether he tells them, it is up to him

I: But they would have even called you if he told them**.** What about your relationship with you and community members or your neighbors where you stay?

R: Those have not been bad, no

I: You know, sometimes when you get problems like this, they begin to segregate you, as you have said

R: No, they have not been bad, sometimes they call me, and ask where are you? we no longer see you, this and that, things like that

I: They also do not know that your daughter is sick?

R: I can tell them that I have a sick person but without telling them which person, which kind of sickness the person is suffering from, but I tell them I am at the hospital my person is sick, and they say ok

I: Haven't they told you to come and see you?

R: They can send me things, like we are sending you this

I: Or they tell you that they want to come, and you tell them that you are in Kampala, so that they do not come

R: They send me some things and tell me we are sending you this, and I also thank them, like that

I: So, from what you have said, it shows that your relationship with people in your community is not bad, it's good**.** Do you have any challenging situations you have experienced with your family or the people you stay with in the community because of the condition of your daughter being that who is sick?

R: Now, that situation did not come to me because when she became sick, we took her to the hospital. Most of the symptoms became hidden, but if we took her to traditional healers, the challenge would have been big because they tell you a lot of things. When they tell you a lot of things and then you see your child is becoming severe, the truth is that you start fighting. But if you come to the hospital, the truth is all of what happened, let me say confidentially, and you just take care of a person

I: So now, how is the plan? Won't you also go to the other side? And see what is going on?

R: Leave that to me

I: I leave it to you, you have decided to keep it to yourself, you have kept a lot to yourself, that is the third one

R: It's not too much, I have answered most of them

I: Ok and I thank you, thank you so much, and when I say something and you feel you are not free with it, you tell me to keep it to yourself like as you have said. Now, I would like

to know, and here I want you to tell me the truth, as I told you I am a health worker and I also told you whatever we say here is between me and you, I would like to know, when you brought her here, how did the health workers treat you? How have they been treating you? That is so important, here

R: Here they have a lot of love, that is what I have seen, because this is not the first time to treat in hospitals sometimes, as a caregiver to my siblings or friends like that. There are hospitals where health workers burst verbally. The truth is, you just look, you brought your patient and are sick, and someone bursts at you and you feel weakened, but they truly treated us well. We reached here, they treated us very well, and they sympathized with us, they also felt bad. They handled her well, they registered us, they took us to the bed where she was admitted, they told us to make the bed, though we had left things on the road side in the car, and they told us to put any sheet we had on the bed, we put it there quickly, we saw that they helped us, the health workers came to see that incase she disturbs a health worker to inject medicine into her, they can handle her, the truth is that it encouraged me, it made me happy

I: That is so good, now, on that day they told you to put anything you had, and you put it there, was she injected with medicine?

R: Yes

I: When they injected her, and you saw that the person who had been shouting kept quiet immediately, what did you think?

R: She was talking too much, but she started slowing down, and the truth is, I said the medicine has power

I: How did you feel as a parent? Did you get worried?

R: I did not get worried, I felt calm, the other shouting was scaring me so much, reason being they tell us that the more a person uses a lot of energy in this shouting, a lot is being used even this water can be used, but I saw her resting and I felt relieved and I said its ok let her rest a bit

I: Another thing I would like to know, I would like to know, is to tell me your experience concerning not being treated well among people or being segregated because of having a child with this kind of mental illness

R: Because this issue was not heard, it did not go anywhere

I: You have not gotten things like being segregated or doing this and that, or someone backbiting you

R: No, the person whom I approached for advice is the one I talked to about it and the one who told me to come quickly to the hospital. She told me that if I go to traditional healers, everyone will know about it because by the time they calm her down, everyone will know what is happening to me

I: Which kind of help should be given to caregivers of adolescents with the kind of mental illness your daughter has?

R: For example, like what?

I: May be from health workers, or people around them, what kind of help should they be given?

R: Medicine is the first, the second thing is clothes, the third thing is food, but the good thing here is that food is given. But if you see that yesterday she ate this kind of food, you say, let me go and buy her this kind of food, but food is always available, so clothes, sometimes, if they are not back to their right state of mind, they tear them. You dress it on them when it is still new, they tore it, and you need to get another cloth. The third thing is money, because there is nothing that can be done without money, that is what I see

I: That is how caretakers can be helped?

R: Yes

I: Ok, what about counseling, what do you say about it?

R: Counseling the patient?

I: Counseling the caretaker, can the health workers or people around the caretaker try to counsel her? Do you see that it helps?

R: It helps so much because it’s not that when a person reaches that situation of mental illness, they will never go back to their right state of mind; the person will be treated and will go back to their right state of mind. The caregivers should not get discouraged and become careless toward that person. Counseling the caregivers should be that she’s not the first person to experience it, no, and will not be the last person. If it has happened, the caretaker should be there and take care of the person like any other illness that affects people, it should be just encouraging the caretaker

I: Ok, and who should encourage them?

R: Anyone who can counsel a person, but there is when a person comes just laughing, saying that it is not an illness, those are family spirits, such things which discourage a person, that situation is bad. A person should encourage one another and calm them down, and they know that mental illness is treated like any other illness, and a person stabilizes

I: There is where you have talked about medicine, where information about the drugs their adolescent is taking should be given to the caretaker?

R: In most cases, they may write for you the drugs and they are expensive. The truth is you fail to buy all of it, and just buy some of it.

I: What about side effects? Or what may come along as a result of taking drugs

R: I do not know that, I do not know whether it's there, on that it's you to tell me, you may give me an example, like what I have ever seen happening with people who take such and such a type of drug, usually brings such and such effect

I: Ok, we shall talk about it, on the same issue about drugs, which type of drugs does your daughter take?

R: I did not study medical language, but it was written for me, I do not understand it

I: You do not understand it

R: I do not understand it

I: Did they explain it to you?

R: The truth is, I do not know it, because I have never seen it

I: What I am saying is that they explained to you that your daughter has been given this type of drug, like when you go to the clinic and they tell you we have given you Panadol and Coartem

R: That one no, but they showed me that this and that will be able to help her

I: Ok, that she should take 2 tablets here and this here she should take like this, they showed you that you?

R: They showed that to me

I: And when she takes them, it can bring this and that, did they tell you about that?

R: They told me about sleep, they told me she will sleep, but if I see too much sleep, I should report it

I: Ok, if I tell you to talk about the kind of illness your daughter has, which kind of mental illness does she have?

R: May be Weakness in brain, that is what I can say, that is what I think, it could have been weakness, its weakness

I: It’s what you understand

R: It’s what I understand

I: Is it what they told you? Did they explain to you the kind of illness your daughter has?

R: They said it, yes, they said it, but I think it would have been like weakness

I: Ok, we have been talking so much about treatment in the hospital, some go for prayers, what do you say about it?

R: Most advises, some people told me to take her to church, like her siblings, they told me to take her to church and I told them, you know, let me first take her to the hospital, after taking her to hospital and prayers will later come when the person going to church understands

I: Thank you for deciding like that, what about those who say it seems this is family spirits, remember yours, they stole her things, brought them back dirty, didn’t you reach there?

R: We had a big challenge, it also came in my heart, it came in my heart, but because I had sought advice from someone, what that person decided for me is what I did. In the morning, we woke up and came here

I: While coming, didn’t you think maybe I am going to the wrong place, I would have done this?

R: No, because the person I talked to told me that that illness, things are now easy, that illness has medicine, but you will take her to traditional healers they will give her some many cups of medicine, you do not know it’s quantity, you do not know the energy the person has, then sometimes she may become worse. So first take her to the hospital, the situation changed, medicine is now there, I also had to listen, because when you ask a person, you are looking for advice

I: Other caretakers of adolescents with this kind of mental illness, like your daughter has, what advice can you give them?

R: The truth is, if they want traditional treatment, they should first bring children to the hospital, and children, stabilizes then they see that later. But first taking a child to

traditional healers, the child takes long to recover and not only that, even the illness, some reach a point of being retarded like that

I: Apart from telling them that, first, going to the hospital, what else can you tell them?

R: Encouraging them, telling them that you are not the first person to get this situation and you will not be the last, so you have to be strong, and take the child for treatment. If it is money, you realize it or you give it there, because some may think that since the person has got a mental illness, they will never stabilize, do not give up on them, they come back to their right state of mind

I: Ok, if you do a flashback, that's where you said you expected a lot from your child

R: Yes, it’s usually too many. Whenever you give birth to your child, there is nothing bad you expect from them, it's only good things

I: It’s true, I would like to know, and now that she got this illness, when you picture in the future, what are you seeing? What is it that makes you somehow worried?

R: Now, what I get worried about or somehow fear is her sitting in class again, may be when they are studying, I fear that she may get the illness - that thing frequently comes to my mind. Let me also ask you, will she be able to perform well again in school? Will her brain function the way it used to before the illness?

I: The important thing is that if she takes medicine, she will be able to sit it class and perform well in school

R: Able to compete in class like the way she used to compete

I: Yes, let me ask, apart from worrying about class, what else?

R: I have nothing, because if she can manage that, then she will be able to do the rest in the future

I: Ok. This thing did not start from school; she just picked up her property, and she came home when she was doing well?

R: She just picked up her property from school, we were happy to see her, and she was hard, the way she would respond

I: You could see that there was something wrong

R: At first, we did not realize it, but we could see that she was hard, but the following morning, she put on gospel music for born again from one song to another song, eehh**....** we said this is much, there, we realized something was wrong

I: What I would like to know, she left school, did the school administration ask you about her? How is she?

R: We just told the school administration that when she left school, she became sick

I: As we are about to conclude, I would like you to give me one example of the most challenging situation you have faced while taking care of her

R: I told you that situation, and any other person can experience it, the most challenging situation and it hurts seeing my child abusing me using big words, words she could not even pass through her mouth while she was well. The second thing destroying things, the third one pouring food, I feel like the situation is getting out of order, and I strengthen myself. Whenever she does it, I say she is not the one. If I give a person food and they eat it well, I give them a drink and they drink it well, I feel good, but if I have given it to them and they have poured it down, I give them a cloth and they tear it down, I feel very disturbed.

I: What do you think caused your daughter's illness? In your thinking

R: I also do not know, that is what I can answer you, I do not know

I: You do not know, there is nothing you suspect

R: I do not know because when I say that it was those clothes, I do not know whether it was the one, or it was initially there before in her head and it manifested at that time, also that I do not know, it's God who has treated for me and the health workers have done some work and she stabilized

I: Ok, thank you so much for answering most of the questions, and you have also given me time, thank you so much. Any questions you would like to ask me

R: I do not have any questions because what you have answered is what I would have asked

I: If it's not there, I request to end here

R: Maybe one thing you have said is that, what things should I expect these drugs to bring?

I: Let's talk about it after stopping the recorder

A QUALITATIVE INTERVIEW ON THE LIVED EXPERIENCE OF CAREGIVER 5 (C5) OF AN ADOLESCENT WITH BIPOLAR DISORDER

Interviewer - I

Respondent - R

I: Just like I had earlier on informed you, I am a Nurse xx, a mental health nurse currently at Mbarara University of Science and Technology pursuing a Master's of nursing science in mental health. As part of my research project, I am currently focusing on the lived experiences of informal caregivers of adolescents with Bipolar affective disorder at Masaka regional referral hospital. Sometimes, as health workers think that they know such experiences, yet they don’t, they don’t know what it means when a child suddenly develops signs and symptoms of mental illness. But before we go further, I would like you to introduce yourself, your name, age, religion, and marital status

R: I am C5, I am 42 years old, I am a born-again, and married in church.

I: You have told me the child is your daughter, right?

R: Yes

I: How long have you been staying with her?

R: From the time of her birth, I have been living with her; she only leaves home when going to school and normally comes back in the evening

I: As of today, how many children do you have?

R: I have four children

I: And you said she is the firstborn?

R: Yes

I: How many boys versus girls do you have?

R: I have two boys and two girls, and that was my dream and prayer, and I have stopped with those.

I: How old is she?

R: She is 17 years old

I: As someone who is currently caring for an adolescent with BD, hearing from you would provide us with a rich understanding of what it means to be a caregiver of an adolescent

with BAD. We would like you to tell us about your lived experience as a caregiver of an adolescent with BAD.

R: It is a very bad experience, because first of all, you feel a lot of pain, it also needs a lot of money to look after the child, and in addition, she doesn't want to take the prescribed medication. Sometimes she beats/fights me, last time she fought and injured my mouth in the process, so sometimes you just feel tears rolling down your face. You see, because as a parent, you have a lot of expectations for your child, when they develop a mental illness, you feel like their future is completely ruined. Just know that I feel a lot of pain, and worst of all, I don't understand what caused mental health problems in my child.

I: Apart from those lived experiences, what else have you experienced in caring for this child?

R: You mean since she fell sick?

I: Yes, that is what I would like you to share with me. Let us talk about such experiences here

R: The experience is a bad one, we left home when she had destroyed (crying..)

I: Ok, let us go slowly, before the onset of the illness, how would you describe Rebecca?

R: What I can say is she used to be a stubborn girl. By the time I delivered, she was underweight, she was below 2 kilograms. She spent almost two weeks after birth without gaining two kilograms; she was born without eyebrows, and she had fingernails by then. But with time, she developed all those things

I: Does it mean that she was born prematurely?

R: No, she was born at term, in the hospital. They told us that she was at term and birth, and she was never put in an incubator

I: Did you have a cesarean section?

R: No, it was a normal delivery, and at nine months she gained a lot of weight – she had 18 kilograms at nine months. Whenever I would take her for immunization, the health workers would inform me that she was healthy. However, at ten months, she experienced seizures. Each time she would fall sick, she would experience seizures, and she also developed pneumonia, which made us spend a lot of time in hospital admission beds. It reached a time and I got tired of the hospital admissions; I was directed to always use tablets of valium, especially when I observed that she is not feeling well, to prevent

seizures. At times, she would be playing with other children, and I would be called by her friends, informing me that Rebecca had lost consciousness. On taking her to the hospital, they would always say that it was fever; she experienced those seizures until she was seven years old because by then she was in primary two class. From that time, she never got the seizures again. she was a very jolly person, but would be restless most of the time to the extent that sometimes you would serve her food and she would eat or drink it while walking. She was a very active child, and people around the market knew her. She used to greet everyone she met because each time she greeted them, they would give her money.

R: So, are you a market vendor?

I: No, I am a primary nursery teacher

R: For nursery class, I enrolled her in a good school, and she stayed with my sister. But whenever she would go to school, she would lose all her items, including the books, pens, and food containers. When she would be asked about, say, the food container, she would reply that she didn't know where it was. They used to release them at 12:30 pm, she would always visit various homes before coming back home, and as such, she would always reach home by 6:00 pm. When I would try to inquire, my sister would always tell me that the child had no problem to worry about. She was then promoted directly from baby class to top class because she was very bright. In primary one, I then transferred her from Butambala at Kaweesi memorial to a school in Gomba in Kanoni and it was their father who used to take them to school by riding a motorcycle. While in primary one, she would still lose the books and the food container; she used to lose everything. Whenever the father would delay picking them from school, she would walk, greeting and interacting with almost everyone without fear, and she would delay on the way. I tried to talk to her teachers, but they also found it challenging to handle her because she would talk a lot and would be restless most of the time. Onetime, one of the teachers advised me to just let Rebecca be the way she was. I was forced to think about taking her to a boarding school with a view that maybe while there, the teachers would help to monitor her closely. While in primary class one, whenever she would go to school, she would remove her books and all other things from the bag and then throw it in the rubbish pit, claiming that we had picked the bag from the rubbish pit before giving it to her. In the

evening, she would always pass by the rubbish pit and pick up the bag, fearing that we would ask her where the bag was. By primary four, the restlessness was increasing. For example, whenever she would arrive at the church before her friends came, she would go for them from their homes to church and then escort them back to their homes after the church service.

I: During that time, how was your relationship with her?

R: Our relationship was good, at first, I used to always punish her, but she would repeat the same thing the following morning, until I had to let her be. When she joined primary four, we took her to Living Hope School, a boarding school in Lusango, together with her sibling who was in primary two. While in Lusango, her restlessness increased; she would always complete assignments at the same time as the teachers and would always disturb her colleagues. Rebecca became a very big issue at school, she would move from one class to another, disturbing other pupils. Good enough, the owner of the school was very strict; she decided to stay with her over the holiday period to supervise, guide, and support her appropriately. She informed me that the owner of the school warned her that if she didn't stop talking a lot, the school owner would pierce her mouth with a needle. Also, onetime she complained to a visitor while there that it was impossible to shut her down because even when you keep silent, deep within the person, you continue talking to yourself. The owner of the school threatened to pierce her mouth if she kept talking a lot, which could have tortured her a lot. By the time she sat for primary seven, she would sit still and talk fewer words. It reached a point where they would give her assignments using textbooks to avoid her finishing before the other class members. She passed primary seven with 11 aggregates despite her target points being 4 aggregates – this caused her to be stressed a lot. I counselled her and informed her that, after all, it was still first grade and that she still had more chances of improvement. We then took her to Yesu Akwagala High School, a place where she had been until she broke down.

I: So what is it that caused you to bring her to the hospital? Did the illness come while she was at school or at home?

R: She got the illness while at school. Last term she had holidays for only one week, she came home and informed us that they had a prom party at school of which we gave her the money for the prom party. We always try to pay her school fees on time, because

when not paid in time, she gets worried a lot. We gave her what we thought was necessary because even during their party for primary seven, she disturbed the lady who had accompanied her to buy clothes. Even her teachers kept informing us about her worrying a lot about something that she feels she needs, and as parents, we always guide and counsel her to avoid worrying a lot. Right now, what I know is that when she wants something from us, she normally communicates with us; she doesn't hide it. During the preparation for the prom party, she told me that one dress would be enough for her, but after 3 weeks she called me and said, "Mum, the students have three different clothes for the prom party, will I just have one dress only for both the red carpet and lunch?" I informed her that at that time, we didn't have money, but I advised her to use what she had in the meantime as we looked for money. She agreed but complained that all her friends had not paid for the prom party, and as such, she wasn't going to enjoy it, and she was asking me what she was going to do about it. I told her that she needed to concentrate after all the prom party was a one-time event, passing the exams was the main priority, and she agreed with me. After one week, she called me again and said that "Mum, I have realized that the prom party is not very important and as such I will add the money for the prom party to the field work activities". I then asked her So, won't you attend the prom party; She replied Of course I will attend. That evening, I talked to her father, who sent her 35,000 shillings. At this time the prom party was now due, as I was home, I received a call from the headmaster who asked me "Mama R, does R have any illness of any sort that normally disturbs her?" I replied that the only illness that R has ever suffered was pneumonia, of which she improved on treatment. He then informed me that R was talking uncoordinated words, he gave her to me on the phone and I talked to her well, but suddenly she started crying and told me that we would talk later. Afterward, I think they requested another contact from her, and she gave them a number for my brother. My brother called me at around midday and asked me if I was aware of what was transpiring at R's school. I said no, then he said that they had called him, saying that Rebecca was talking incoherent words, and yet his father's contacts were off. So, when I went back home at around 5:00 pm, her father informed me that the school administration had called him, informing him that R was talking uncoordinated words and that we needed to pick him from school. But since it was late, he suggested that we could pick the

following day. On Saturday, as I was waiting for the Boda-boda guy, the school once again called me and said we needed to hurry and pick Rebecca because she was now fighting her classmates and destroying property. By the time I reached school, she was very aggressive, and her mental status wasn't good at all. I suspect that maybe it was related to the prom party, because among the words he was talking, often things related the prom party were the majority. I brought her to Nkozi hospital, thinking that maybe she had malaria, but on checking her, they confirmed that she didn't have malaria. So, they treated her and discharged her back home. She had improved and regained her senses. So, I asked her what she thought had happened to her. She told me that when the parents of her friends failed to pay for them for the prom party, she felt very bad. She further narrates that when this happened, she called her friends and suggested to them that they could request permission from the headteacher to hold their party at the church, as the other students hold the prom party at school. She started by telling her class teacher, who discouraged her but referred her to the director of studies (OS). She proceeded to the DOS and informed him about their plan despite the fact that she had paid for the prom. The DOS informed her that it wasn't possible, and he too referred her to the Headteacher. When she proceeded to the headteacher, I think the headteacher realized that she was not doing well mentally and thus called in counsellors to talk to her. So, her request was never successful, and from that time, she felt a lot of burning sensations in the head and requested her friends to pour water onto her head. They refused to pour water on her head, and she had to pour it on herself as the other students laughed at her.

One of the students reports that after those meetings, they proceeded to a discussion group, and it is during this discussion that she climbed and stood on top of the table which made them to wonder a lot. So that is what we have gone through, I have also had challenges with finances along this journey.

I How about loosing out time, what is your comment on that?

R I put all the other things on hold in order to look after her

I How about your teaching job, isn't it at stake?

R The good thing the director is aware, and luckily, I had been appointed as the headteacher so I have not been into active teaching. So, everything is moving on well. So, my job is

on hold yet I need money to look after the child and also her siblings are at school and need money – so it is very challenging.

I: Has the child's mental health condition affected your relationship with the immediate family members in any way?

R: It has not affected my relationship with the family members, they are supportive so far

I: How about your relationship with the neighbours?

R: There we have a challenge, remember we are born again Christians, so people are talking many words, some are jubilating because she is sick, some are telling us that despite being born again, we should take her to traditional healers for treatment. Some are happy because they think that she has been boasting about being very bright. So those words are not good; they cause a lot of pain. But as a believer, I know that Rebecca will get better. You see, another challenge is that we are alone, on both the father's side and my side, we grew up without parents because they died when we were very young. So, my hope has been so much in my children so when they begin to get mental illness, you really go through a lot of pain which is not easily explainable to another person. We were born four children, two are abroad working, and one is in Kampala, so sometimes you feel lonely and fail to get a close relative to share your problems with. Sometimes you are forced to keep your problems to yourself because you have no one to share them with, so right now I am the only one who can understand the pain that I am going through as a person. You try to get friends, but it is also not easy because some are not trustworthy.

I: So, do you know these people who speak those words?

R: I know them. When we were in Nkozi, there was a neighbor who visited us, and when she went back, she informed those people that Rebecca was throwing stones at me, something which wasn't true. So, when they talk like that, you really feel very bad.

I: How have you been coping with such situations?

R: Since my adolescence period, I have been a born-again, so when they talk such words, I believe that the Almighty God will heal me.

I: When you brought her to the hospital, what was your experience with the health workers?

R: I have not had any challenges with the health workers; they are doing their work as they should. And when it calls for strictness, they are strict, especially with taking medication,

so they have been very good to us. In fact, at one point I even thought that if I had known and come here immediately, maybe she would have improved by now

I: After the assessment of the child, did the health worker inform/explain to you the condition of the child?

R: Up to now, I have not had a chance to get a health worker to inform me about the exact condition that my child is suffering from. I am eager to hear from them because they are the experts in that field.

I: So, after the assessment, what did the health worker tell you?

R: After the assessment, the doctor sent me to buy the medication to use to treat her. But I have a lot of hope that she will get better since she is in the right hands. However, the health worker at Nkozi hospital informed me that children who have ever experienced seizures sometimes re-experience them between the ages of 17 and 23 years. He further said that some people end up not taking these children to the right treatment areas, which causes them to deteriorate. He assured me that my child will recover, though it will take some time, and he also informed me that they will be giving return dates for review of which I accepted.

I: Ok, I will elaborate more on some of those things when we are done with the interview, because I still have some questions for you that also contain some information on that. As regards medication, did the health worker explain to you more about, say, the side effects of the medications?

R: No, they did not, since I came here, they have been giving her medication, but they have not explained to me what the medication does, including its side effects

I: Do you know the name/type of medication that she is on?

R [Laughs] I am not sure of the name, it is a hard name. I can ably identify them by color and appearance. I also try hard to note down the type of drugs she is taking.

I: Have I ever been isolated or stigmatized because of having a child with mental illness?

R: No, I have not faced that yet, and good enough on the ward, we are all caretakers and no one stigmatizes the other.

I: Let us revisit the day you came here; did you encounter some patients who were badly off?

R: Based on how my child was by that time, some patients were badly off

I: Didn't you get worried a lot about your child?

R: No, I didn't get worried because even before bringing her here, we had proposed to take her to Gombe hospital because it is near home. But when I discussed with the health workers, they informed me that she would not benefit a lot from Gombe hospital, they said she needed a specialized hospital handling that. So, when I came here, I just felt that I was in the right place for my child's illness.

I: From your thinking, what do you think caused this child's mental health problems?

R: I think it is the devil who has attacked my child, you know we pass through very many challenges, and the devil doesn't wish us well, he came with three missions: to steal, to kill, and destroy. I think it is the devil that attacked us, thinking that we deny God, which won't happen.

I: Could you tell me the most challenging situation that you have gone through while caring for that child?

R: The most challenging situation for me is her refusing to take her medications as prescribed. When you tell her to take her medication, she refuses – that stresses me a lot. Yet when a person takes their medication well and eats well, they recover very quickly.

I: How about eating food?

R: Today she ate food, the challenge I have with her is that she wants to eat expensive foods such as meat and chicken. When you give such type of food, she eats it well

I: Meaning she is not interested in the food that is served here?

R: For posho and beans, she told me that she will eat them while at school, not here in the hospital.

I: In this situation, are there times that you have found yourself thinking about what the future holds for your child?

R: Yes, I thought about it. Whenever I have seen children in the age bracket of my daughter with mental illness, I would think that they should have done something wrong, maybe they bewitched someone. But surprisingly, despite harboring such thoughts towards them, they keep coming near me. For example, when at home, you cannot cook food and eat it alone without sharing it with a child with a disability. Even the people we employ at home are disabled, including the one who rides me to school. There is one who normally comes home very early, and I always look after him. So, when my child developed this

illness, it reminded me to remember all those cases. At school, I have a parent who has a mental health problem; she has two children at our school. Sometimes she comes to school just to purposely talk to me, and when you have some food or money, you give her. So, when my child got this problem, I got worried and thought for a second that let me hoped my child would not reach the level of these other children. So, when at one point I thought that her future might be ruined, but then I remembered what the doctor at Nkozi told me (that she will improve if she takes her medication well).

I: What kind of support do you think should be given to a caregiver of an adolescent with BAD?

R: First of all, my humble request is that let the medication be available in the health facilities because the caregiver spends a lot of money on the caring process. Caregivers normally spend a lot of money when still looking for help for their children, so by the time they get to the hospital, they have spent a lot of money. So, it becomes very challenging to buy the medication.

I: How about counselling, do you think that the caregivers should also receive counselling?

R: Yes, they should receive counseling to help them settle down. If not counselled, it makes you get a lot of memories about the past. So, the caregivers need to be counselled and supported even when not supported financially. You can even support someone with money, but they will remain with their thoughts, which is a very big stressor.

I: What recommendations do you give to other caretakers of adolescents with BAD?

R: First of all, they tolerate these children. Being too strict is good, but it can hinder treatment. Secondly the caretakers should keep patients' information as confidential as possible, even when the patients seem to be very mentally unstable. Whenever you talk about their private information with others, it makes them feel very bad. There is one thing that I am still struggling to learn from my husband: whenever he encounters a person who is struggling, he never demoralizes that person, instead, he instills hope even when he knows that you is on the wrong side. Some caretakers blame their adolescents immediately, even when they are too stressed. So instead of blaming them, it is good to continue encouraging them, showing them that it is possible to overcome the situation they are in.

I: At one point, you talked about prayers. Did you embrace this?

R: Very much, even when I am giving her medications, I pray to God to help me. Even when I am beginning my day, I normally pray to God to help me be able to navigate my day. It is very important because it helps to gain strength as a caretaker.

I: Sometimes, when some people have children with such mental health problems, they tend to first seek help from traditional healers. Did you encounter a similar approach?

R: No, I could not begin from there because my uncles used to use traditional healers a lot, most of them have died because they used to believe in traditional healers a lot. Sometimes, even when they would be told that they have diseases such as cancer, they would still believe that they have been bewitched. So, I saw people using traditional healers with no improvement, and I got tired and fed up with such behavior. I don't believe in those things, and I will never use them

I: Ok, do you have any questions related to what we have been talking about?

R: Despite being a very strong believer, I still wonder and want to ask the doctor if my child will get better.

I: Ok, thank you for that question. I am going to answer when we conclude/ end this interview

R: Ok

I: Thank you so much R's mother, for elaborating on these issues as you have done

A QUALITATIVE INTERVIEW ON THE LIVED EXPERIENCE OF CAREGIVER 6 (C6) OF AN ADOLESCENT WITH BIPOLAR DISORDER

KEY

Interviewer: - I

Respondent: - R

I: I am xx a mental health nurse, but currently a student at Mbarara University of Science and Technology. I am going to have an interview with you where I want to know more about your daughter, but most especially, I want to get to know about your experience as a caregiver for a child with an illness like the one your daughter has. Before we go any further, I would like to know your name, age, and sex.

R: I am C6. I am 74 years old.

I: Sex.

R: By that, do you mean my tribe?

I: No, are you male or female?

R: I am female.

I: What is your religion?

R: I am Catholic.

I: What is your marital status?

R: My husband died.

I: Sorry. How far did you go with education?

R: I did not go far because back then our parents only educated the boy child so I only studied confirmation, although I can write a little.

I: What class did you stop? Was it called Junior back then?

R: Back then, we only studied confirmation.

I: How do you call SS?

R: She is my grandchild, a daughter of my son.

I: What is her full name?

R: She is SS

I: How old is she?

R: She is 18 years old.

I: Oh, that is good. I would like to know how long you have lived with her?

R: She was handed over to me when she was 1 year old.

I: She has lived with you since then, apart from when she goes to boarding school.

R: Yes, that is right.

I: Ok. You, as a caregiver for a child with mental illness that presents in two ways, depression or too much excitement or excessive anger, hearing from you could help us understand what it truly means when someone takes care of someone with the type of mental illness that your daughter has. We would want to know about your experience while taking care of that child.

R: From the start?

I: Yes, from the time she fell ill and before she fell sick.

R: She used to be calm and didn’t have so many words, but ever since she fell sick, she started talking a lot and having a lot of anger. When you tell her something, she doesn’t listen. She currently doesn’t have excessive anger; she has calmed down. The only problem she has now is oversleeping. when you tell her to read her books, she doesn’t listen but tells you she will read at school. When she sleeps, it is hard for her to wake up on her own. You have to go to her room and wake her up, and you have to insist until she wakes up to prepare for school.

I: Isn’t she in boarding school?

R: No, the teachers refused to accept her in boarding because she disturbed them a lot when she was there, so she is now in a day school.

I: You have talked about her disturbing the teachers, was that before or after the illness?

R: Yes, there was a time she relapsed and kept climbing over the fence to come home, but on getting here, she would run back to school. When she would come home, she would say she wanted to go back to school, but when she would go back, she would say she wanted to come home. The teachers didn’t like that because she disturbed them a lot. The headmaster didn’t even want her to go back there for senior five, but the director allowed her since she had been in their school right from senior one. She hasn’t had any issues ever since she started senior five, she no longer has anger issues.

I: Thank you for that information, but apart from that, what else have you experienced while taking care of her?

R: The challenge was that she was stronger than I and could even beat me, but I have another grandchild around who used to help me with her. They would restrain her, and she even had scars on her hands. I feared her because she was too strong and used to hit people with stones, but when she started medication, she started to calm down slowly. There was a time I was fed up and told her father to take her to Butabika because I knew they would admit her for some time, but when he took her, they only gave him medicine and told him to bring her back. He brought her back to me. There was a time she held an axe, wanting to break the door to get out, and we removed it from her. She had a lot of energy, but the medicine has reduced it over time. She didn’t treat me so well, she disturbed me a lot. If I were to give up on her, I would have done so, yet none of the parents wanted to take her, so I had to persevere and take care of her.

I: You have talked about the fact that sometimes she wants to fight with you. Apart from that, is there anything else, for example, abusing you?

R: No, the only thing she used to tell me was "the SS you know is no longer the same. I changed a long time ago, so don't assume that she will ever come back". She would say things like that, but she would never abuse me, apart from getting aggressive.

I: You mentioned that before she fell sick, she was calm, how did you feel as a parent when she started showing signs of mental illness?

R: I felt so bad because I thought of so many things. I started fearing the school thinking they had done something to her, we thought she had been bewitched. I thought of so many things. She used to talk a lot to the point that her lips would dry so I ensured that I made juice to keep her hydrated. She never wanted to sleep but this people at Masaka hospital have done a lot because they have treated her and she can now come on her own for refills unlike the times I used to bring her. She used to disturb me when it came to taking the medicine but these days, she is self-driven because she takes it at night.

I: Tell me about the onset of the disease, did all the signs come at once?

R: It took me a while to notice but I knew she was a calm child, when she started talking a lot saying they had night dancers at school I started to worry. That is how she started. She started by talking too much and would even talk about things she had heard so long ago. There was child in our neighbourhood that had been abandoned by the mum and had got burnt and she used to tell women to take care of their children while using this child as an example. She used to talk a lot and people would gather yet when you would want to take her to the house she would not accept.

I: How did you feel when she would start talking and people gather?

R: I would feel bad, I think she is the reason I got hypertension. I get my treatment from here.

I: Were you too worried?

R: Yes.

I: At what point did you decide to look for help for her?

R: I didn't know there was medicine for her, so I first took her Masaka. When I reached there, I was told the mental health section had been taken to Kyabakuza, so we went there

and started treatment. We used to go back for reviews until I was told that there were services in Bukulula, so we started coming here.

I: Before thinking of coming to the hospital, how did you think of coming to the hospital, because sometimes people explore other options before coming here.

R: We did everything because I even took her to traditional healers who gave her medicine, but there was no change.

I: Tell me about what the traditional healers told you.

R: Those people told us there was a person who had sacrificed her to the evil spirits because they wanted riches, but when they tried to treat her, and we saw no improvement, we never went back.

I: How about the money you spent?

R: We spent a lot of money until we reached a point of not going back.

I: Sometimes people go to the religious side.

R: On that issue, her grandmother once took her to Pastor Tom in Mutundwe, but when she got there, she told her grandmother she wanted to come back home. She told her that if she didn't take her home, she would get lost, but the grandmother tried to convince her to stay. She found a way of asking a certain lady for her phone so she could call me since she knew my number off the head. When she was given the phone, she called me, saying that if we didn't pick her up, she would get lost and we would never see her again. She said she was tired of the place because of the poor sleeping conditions. I then called her father and asked him to pick her. He gave her transport, and she came back on her own. When she got home, I asked her whether she had left her grandmother there, and she said yes. She told me they used to wake up at 5:00 am and go out in the cold, so she could not manage that, but she left her grandmother there because she was used to the situation. We tried everything.

I: When you took her to the hospital and she was given medicine at Kyabakuza, didn't you find patients who were worse than her?

R: We found them, but when we went back for review, we found that the ones we had left badly off were now much better than SS.

I: When you saw worse patients on the first day, didn't you get worried?

R: I wasn't so worried because I had seen they were worse than her; there was one I saw taking care of two patients, so I decided to be strong.

I: Tell me about your daily experience in taking care of this child.

R: The challenge I have is getting her money to use while at school and also waking her up in the morning. The problem is that I have to get out of my bed to go to her room to wake her up, because if I call her while in my room, she doesn't wake up.

I: It makes you similar to a student.

R: Exactly. It is challenging, and after she has prepared herself for school, she asks for money to spend at school. You have no choice but to give her.

I: How much do you give her every day?

R: I give her 1000/= to spend for two days, but sometimes she tells me she spent it all in a day.

I: Does her father send you some money?

R: No, he doesn't.

I: From what you have told me about waking up early to wake her up, it seems you also have financial constraints, and it seems to be a big issue.

R: It is because it looks like the father left everything to me.

I: How about school fees?

R: I am the one who pays, although she also has sponsors.

I: Do you find that you spend a lot of time taking care of her, yet you could be doing your own things?

R: I no longer spend a lot of time taking care of her these days like it was back then. I had stopped working and would stay home, but now that had changed.

I: Did you find challenges with transport, for example, when you used to go to Kyabakuza?

R: The challenge was there, but I had to struggle to get it.

I: Was there a day when you failed to get transport?

R: No. I always made sure there was transport to go for reviews, even if it meant borrowing. We never missed any appointments.

I: How about the times you would be told there was no medicine, and it meant you had to buy it since she didn't have a job?

R: I would buy the medicine because there has even been a shortage of medicine here before, and I would buy the medicine.

I: Are there times you felt inconvenienced?

R: Yes, but I still had to buy it because I didn’t want her to relapse.

I: Apart from what we talked about, are there any other challenges you have faced?

R: Apart from the fact that her parents do not support me, I don’t have any other challenges.

I: Is her mother also alive?

R: Her mother is alive and tries to help a bit, but has other children to take care of.

I: Are the parents living together?

R: No. they separated.

I: You said the father is your son, right?

R: Yes.

I: I would like to know about your relationship with the child before and after the illness, and also during this time when she is on medication.

R: She loves me, and I also love her. She listens to me now, unlike the time before she started medication, when you would tell her something and she wouldn’t listen. We have a good relationship.

I: Ok. You have talked about your relationship with her parents, and it looks like it is not so good.

R: It isn’t so good because they left her to me.

I: Do you think they left her to you because she has an illness?

R: No, I think it has to do with money. My son shows me that he doesn’t get money, and since I don’t go to where he works, I don’t know how much he earns, but he shows me he doesn’t have money.

I: Doesn’t he have other children?

R: He does.

I: Where do they stay?

R: With their mothers.

I: Doesn’t he have a woman he stays with?

R: He used to have one, but I don’t know whether they are still together.

I: So, the relationship hasn’t been good.

R: No, it hasn't.

I: Do you think it is because of the child?

R: No, he just tells me he doesn't have money.

I: How is your relationship with the people in the neighbourhood?

R: The relationship isn't bad. I have no problem.

I: Have you been discriminated against because you have a child with a mental illness? Are there people who have said harsh words to you?

R: They said harsh words at the beginning, but not anymore.

I: Please tell me about the beginning.

R: They said that she could have been sacrificed for riches.

I: Were they talking about you?

R: Maybe her family.

I: Were these people from the neighbourhood?

R: Yes, but I left it to God.

I: How did you feel about that?

R: I didn't like it, but I never confronted anyone. I just kept quiet.

I: How did you react to all those comments people made about her when you were out doing your things?

R: I heard everything, but would just keep quiet.

I: Did anyone direct you to Masaka hospital?

R: I first thought of taking her to Dr. Ssekitoleko after a recommendation from a headmaster, and I took her. He treated her, but she didn't get better. I was then told to take her to the mental health facility in Masaka, but when I got there, I was told they had been taken to Kyabakuza.

I: How were you treated when you got to Kyabakuza?

R: I wasn't treated badly because when I got there, I was told to buy some injectable medicine to help her calm down since she was talking too much. After she calmed down, we were given tablets.

I: Were you admitted?

R No. By that time, they didn't have beds for admission.

I: From your point of view, how did the doctors treat you?

R: They didn’t treat me badly.

I: After they saw her, what did they tell you?

R: They gave me medicine and told me to give it to her as prescribed, and she would get better. I got testimonies from some people at the clinic that the medicine worked, so I was encouraged.

I: What I am trying to ask is whether they explained to you about her condition and told you what she was suffering from.

R: I didn’t get any information, and that is how I suggested she be taken to Butabika because I thought they had machines to check what was wrong with her head, but they also just gave her medicine.

I: That means you went to Butabika because you were not satisfied with what had been done at Kyabakuza.

R: I was satisfied, but I thought when they got to Butabika, she would be taken off medication and put in a machine to find out what was bothering her, but when they got there, they found worse people, and some were even naked. Some had been admitted, but she wasn’t, and the medicine they gave her looked like the one she was given at Kyabakuza, so we didn’t go back there. We thought we would get something different from Butabika, which wasn’t the case.

I: What do you think she is suffering from?

R: I think the issue has to do with her brain because she isn’t in any physical pain. I think her brain got messed up in my point of view.

I: No one has ever explained her condition to you?

R: No.

I: Did they explain the medicine’s mode of action and side effects to you?

R: When I was given the medicine, I was told she might get side effects like excessive saliva, tremors, so I was given a drug to give her twice a week to help with that; she takes it on Monday and Friday.

I: Didn’t that make you worried?

R: I had to bear with it because there was a time, she had started hating school because her hand would shake and children would laugh at her. She told me she was tired of school because every time she would hold a pen, her hand would shake, and people would laugh

at her, so when we came back, we were given that tablet that would help with the issue as time went on. She no longer has tremors.

I: Thank you for that information. Have you ever been discriminated against because you have a child with a mental illness?

R: No.

I: What kind of help do you think caregivers for mentally ill children should be given?

R: I think we need help in educating them and transportation to the health facility.

I: How about when it comes to medicine?

R: In terms of buying it?

I: Yes.

R: If it were possible, they could give us money to buy it in case it is out of stock.

I: How about counselling?

R: Even counselling, although they already care about the patients.

I: I mean counselling for the caregivers.

R: I get counselling from the nurse here, not to worry a lot about Sheebah.

I: Ok. What advice to you give to your fellow caregivers for children with mental illness?

R: The advice I would give them is not to abandon those children and to take them to a government hospital for treatment, and not anywhere else.

I: What do you mean by not abandoning them?

R: I mean, not leaving them unattended because they may wander off, and you have to look for them. I had a challenge with SS because she would tell you that she was going to the toilet, only for her to wander off, so they need to pay keen attention to them and also love them.

I: How does loving them help?

R: It helps encourage the child because some even beat them, but you have to be caring, reassuring, and also ensure they eat and drink a lot since they talk a lot and their lips tend to dry.

I: Let us try to look at the future, as her grandmother, I know you have expectations of her. I would like you to tell me about what you envision her future to be.

R: Since she was a bright child, I used to think she would grow up and start taking care of herself but now that she is on medication, I think there will come a time when she is

mature when the medicine will negatively affect her and she gets to a point where she can't take care of herself. I usually wonder whether she can stop taking the medicine at some point or she has to take it forever.

I: Are there times you worry about her getting a job or getting married?

R: I keep praying that she finishes school, gets a good course, and finally gets a good job. I don't think a lot about marriage.

I: Do you get worried that she might study and fail to get a job because of her illness?

R: I worry about that sometimes and even lose sleep. I have tried asking about what she thinks of that because she wanted to study medicine but failed at Maths, and when that happened, I tried telling her to go into hair dressing, tailoring, and she refused both options, but chose to go for A-level. I worry about what she will do if she fails at senior six.

I: Since she is getting older, do you ever have talks about boys with her as her grandmother?

R: I try to talk about early pregnancy and the dangers, but she has not yet started engaging in such things, because I would have noticed.

I: Can you tell me about the most challenging situation you have experienced while taking care of this child?

R: I have gone through a lot.

I: You can choose the most challenging situation.

R: She used to beat me, and when we came here, she was counselled against doing it and the results that would come out of it, for example, I could die, and she wouldn't forgive herself for killing me. I didn't like the time when she was aggressive towards me.

I: Do the two of you stay alone?

R: We stay alone during school days because the other child is in boarding.

I: Are there times you were in fear that she would hurt you?

R: I was worried and ensured that I hid all the possible weapons.

I: What were your thoughts?

R: I thought she would cut me.

I: As we conclude, what do you think caused SS's illness?

R: Sometimes I think she was too bright, and this caused her brain to malfunction.

I: Apart from that, is there anything else you can think about?

R: No.

I: Do you have any questions?

R: Will there be a time when she will be taken off the medicine?

I: First, she has to take the medicine as prescribed, then with time, the doctors might decide to step down the dose or change medication according to the side effects. You have to communicate with the doctors and tell them about how the medicine treats her. We can even give her a drug holiday after explaining to you.

R: I think about it a lot.

I: When you look at other children in the neighbourhood who are in her age bracket, how do you feel when you see them doing much better than her?

R: I don't feel so bad because she is in school. It would have been a different case if she weren't in school because she is currently the only one in her age bracket who goes to school in our neighbourhood. The rest are into hair dressing and tailoring.

I: How did you feel when the school refused to admit her?

R: I wasn't happy about it. During a career meeting, the director also knew she wanted to study medicine, but he told us it wasn't possible because of her grades. He didn't want her to do hairdressing and proposed catering, which she declined. She then chose a combination, but when I went to the headmaster to ask about the requirements, he told me she wasn't going to study senior five and that she should do a course and chase us. We left, but as we were going home, I told her we should go back and talk to the director. We went back after I had called to ask if he was still there. When we got to his office, I told him the headmaster had refused to give her a place in senior five, and he told me there was no way he could stop her from studying because she fell sick at their school, and they knew her condition. He told me to go and look for the register here, then look for the requirements, and he would talk to the headmaster. He had an unpleasant discussion with him, and that is how she was accepted into the school.

I: Thank you so much for your time.

A QUALITATIVE INTERVIEW ON THE LIVED EXPERIENCE OF CAREGIVER 7 (C7) OF AN ADOLESCENT WITH BIPOLAR DISORDER

KEY

Interviewer: I

Respondent: - R

I: I am xx, a nurse currently a student at Mbarara University, undertaking a course in mental health, in particular doing research on caretakers of adolescents with BAD, which usually presents with a child being depressed or too excited or being aggressive. In that regard, I will be asking about your experience as a caretaker because when we have children, we have so many expectations for them. Before we go so far, I would like to know your name.

R: I am C7

I: How old are you?

R: I was born in 1959.

I: What sex are you? Male or Female.

R: I am Female.

I: What religion are you?

R: I am Catholic.

I: Ok, how about your marital status?

R: My husband died 20 years ago.

I: Oh, sorry.

R: It was 1999. It’s been about 25 years now.

I: Oh, sorry. What’s your education level?

R: Senior four but didn’t sit for the exams. The person who was supporting me financially ran out of money.

I: How old is the adolescent that you are looking after?

R: He is seventeen years old

I: How long have you been with this child?

R: That child was okay when I was with him, later he went to stay with the father since the father was closer to the child’s school, and used to visit me every once in a while. After a while, he stopped going to school after primary 7, but continued staying with his father. I wanted to take the child to a technical school, but the father wanted him to start doing

manual labour. He first took the child to Kampala and later Ssesse, and he would come visit once in a while, but after some time, I went about a year without seeing him. When he came back at the end of last year, he stayed at his father's home and sometimes at my home. After some time, he said he was going back to work and left. So, we do not know whether that is where the illness started from or not, we are not sure because they say when he came, he was captured by some men who took him to the police, who found out that he was not guilty but it was the employer who was not paying him. I could see that the mind was not the same. He was brought to us after being beaten by Luwungwe, but was picked from Lukerere as you head to Kalungu. He was brought, and my younger children brought him home, where we took care of the wounds he had sustained. The person who took them to work sent money for treatment, and we took him to St. Aloysius Hospital next to the church, and he improved. When he improved, I told the mother that the child could be having an issue with his brain, and the mother said she would consult the child's father. She went and came back the next day, and we were told that he had been seen in town acting strange. He was dancing, breaking sticks, and never wanted to see his mother. He came and told me, "Grandmother, these people should leave me alone, I don't like them. They took me to the shrine…" and I told him it was ok. We later saw he had completely lost it. They had directed the mother somewhere, and I told her we should go to Bugudo, and we would get medicine for mental health. She first refused and said, "Don't take my child to the whites," so I told her to keep using her local medicine while we also use medicine from the hospital. He kept manifesting some signs and symptoms, he would go with clothes to his father's place and come back without them, having thrown them in the toilet. He didn't want us to do his laundry and would throw the dirty clothes in the toilet, so we would get ashamed seeing him naked, and we would give him other clothes. He would throw all the clothes and shoes found in the basin into the toilet and break the basin until I told his sister to tie him up. We would release him during meal time. I would bring him to the hospital despite the mother's refusal, and he would show improvement after taking medicine. The mother would make him take the traditional medicine, which he did not like. When he got better, he would go help his father harvest coffee and sell it. He would get about 25000/= and keep it in his pocket. Little did we know there was something he wanted to do. He would go to the chemical shop and ask for pesticides, buy and take them

to his father's home, and then come back home. He pretended again that he had been sent for medicine to fumigate the banana plantations at both the father's and grandmother's homes, yet he had his plans of killing the people who kept tying him up. He paid 20000/= for it. He came after breakfast, and I had given him his medicine. I would wake up at 6:00 am to prepare breakfast because I never wanted to give him medicine on an empty stomach, since I knew the medicine was strong. So, I called him "KK, tea is ready" he told me he was coming yet he had his agrochemical he had bought. The mother had put a kettle of tea on the fire and he poured the medicine in it and also poured medicine in the jerry can of drinking water then came to me. What helped us was that the younger children at home saw things in the tea while mixing their tea on smelling it, Faradine [agrochemical] was smelling and one of them called their mother. When she came, she asked Karim whether he was the one that had put it and he denied but after she left, he came to me and said "they have been annoying me so I wanted to kill them but grandma don't ask them for water because I also poisoned it". The lady that sold the medicine to him had already told me so when I asked him where he got the money, he told me he had got it from the father's coffee. I asked him why he wouldn't forgive them and he said they are annoying and bossy and they didn't want me to take him to hospital instead wanted to take him to illuminati yet it's not what he wanted. Later the mother asked him again whether he was the one that had put medicine in the tea and he said yes but that it was something that kept telling him to put the medicine so that they all die and he remains with his grandmother. So, I remained worried how he would cope after I die because I always prepare his meals and he can eat whenever he is ready but that poisoning incident scared me.

I: Were you scared he would also try to poison you?

R: I was scared, but he reassured me that he would not kill me since I was the one taking care of him, but he said he would kill them. The sister used to beat him. She did not know that you should show extra care to a mentally ill person and show them love, and he would not eat at her place, although she lived close by. He kept taking his medicine both from the hospital and the one his mother brought. We kept going to the hospital for reviews, and he would talk to the medics well. There was a time he had malaria, but the medicine was not at the facility, so his uncle bought it. When we reached home, he refused to take the medicine but told the mother to pay the uncles 15000/= used to buy the medicine. He was

not sick, although I had told the doctor that he had malaria. His mother said she wanted to be the one to give him the medicine, and despite advice from the uncle, she still took the medicine from me, and he told her he was not going to take the medicine if it wasn't the grandma who had given it to him. She was then reminded that he did not like her and had even tried to poison her, so she returned the medicine. He kept taking the medicine well, but when it was time for the review, he refused to come, so we missed that month because he said he was ok.

I: When he told you he was fine, how were you feeling as a caregiver?

R: I was not satisfied because I told him that even if he had recovered, he was going to be like an HIV positive patient and would have to take the medicine forever, but he refused. Whenever he would feel the pressure rising, he would run to his father's home for like a week. They had wanted to refer him to Masaka hospital, but he refused. When he reached the father's home, he relapsed since he was not on medication, and I asked the mother whether she had now proved that he needed medication. I went to pick him, but while on the boda boda, he stood up and I was scared we would have an accident, so I asked his sister who he feared to come and sit in my place, and I got another boda boda. When we got here, he was sedated, and I apologized to the doctor because he had not been taking his medicine, and I explained to her. When he woke up, we were told to buy more medicine. He kept manifesting some symptoms, but after 3 days he stabilized since he was taking medicine, but from time to time he would relapse. He even started washing his clothes and also went for Eid prayers and came back home. I kept giving him his medicine and meals, but whenever the mother called him for medicine and food, he would refuse. I started getting worried because she would start thinking that I had taken her child from her, but even random people would tell her to thank me for taking care of her son, and yet I am not her real mother because I found my husband already with a child. Sometimes he reaped the electric wires and broke the television, which I am yet to replace, so I called some boys to help restrain him on the window, but not so tightly. I thought to myself that this is what God has desired for me, but people encouraged me that that was not the case. I was told today it was me, and tomorrow it would be someone else, so I became strong. He later told me he wanted to go to his father's home, and I let him. When he reached there, he asked for a polyethene bag, and the father stayed in Kyambala down there, and he put on some

clothes. That was Wednesday, but after a few days of no word from him, I told the mother to go to her husband's place and confirm whether he was there, since it had been 2 days. When she reached there, he told her the boy had asked for a kavera, and people around said they had seen him carrying a bag heading somewhere. I called a few people that I knew were there, and they told me that he had said there was no money or work there, so he was headed back to either Ssesse or Kalungu, but they said he must have gone towards Ssesse. Last week, I called some of my people in Ssesse, and they said they hadn't seen him. Recently, I told the mother that I should probably go to Kalangala for about 2 days to search for him, since a mentally ill person cannot get lost, but when I called the people there and they said they had not seen him, I lost hope.

I: Ok. So, apart from what you have shared, is there anything else you have gone through while taking care of that child? Please share.

R: Yes. Being destructive. Spoiling my things, but when it comes to feeding, I am not bothered because he eats whatever I have, but spoiling my property, and yet, from the start, the mother never liked me since I was her stepmother. Since she was my husband's blood, I persevered and she tried to adjust. The boy spoiled some of my doors, and the electrician said I need 20000/= which I have not got yet. He broke the window, broke the wooden door, broke the tv, threw my shoes and clothes in the toilet, even those of other children, broke the basins and buckets. When I would tell him that I would stop loving him, he would apologise and say he did not know why he was doing that.

I: I would like to know how he was before he got the illness, and how your relationship was.

R: It was good. Whenever he would come to visit his mother, he would also come and greet me, although he grew up with his father from the age of 5. Even when he would come for prayers, he would come and greet me.

I: Ok, so how about after the illness? Did the relationship remain good?

R: Yes, it did, and those that did not like me, he tried to poison them and get rid of them because they mistreated him, yet I never did, even after he threw my shoes.

I: How about after starting medication?

R: It remained. His mother was not allowing him to take the medicine, so I was the one giving it to him. Even when I lost someone and travelled for the burial, I left the medicine with her, but whenever it was time to take it, he would call another child to give it to him.

I: Apart from spoiling your property, what is your daily experience in taking care of him?

R: I beg your pardon. What kind of situation?

I: Daily experience, for example, challenges, financial.

R: He would ask for fried fish, which I did not have, so I would give 1000/= to someone to go buy it since I did not want him to go to the shops. He would request fish and would thank me for taking care of him, unlike those who would beat him. Every time I would ask him not to do something and he refused to listen, I would threaten to call his sister, and he would stop because she had been beating him. I told them to take him to their home if they wanted to beat him, because I wasn't going to allow them to beat him at my home. I did not want him to have both the illness and wounds, and even asked whether they were happy during the time he had wounds, and the money we spent on treatment. We spent about 150000/= treating the wounds. I told them that no one wants to become mentally ill, but God just brings it. They said he does things knowingly. I asked whether someone can play in dirt, undress, or go breaking trees knowingly.

I: Were you trying to explain to the mother?

R: The sister, because she has been the one beating him.

I: Are there times you found that you were putting in a lot of time taking care of him, and even ended up giving up on some personal things?

R: Yes. I could be doing my work, but I was so scared of leaving him, I thought that if I left him behind, I would find that he had beaten a child. There was a time he beat a child who was going to school, and when I asked him why, he told me he was not beating them, but that they were going to school walking like they didn't have legs and would be punished for arriving late.

I: How about in terms of transport, what do you say about that? Bringing him to the hospital and also picking up refills.

R: Sometimes, money is scarce to bring him to the hospital. Sometimes his mother would help. Sometimes when the boda rider would ask for 10000/= I would tell him that I would first give him 5000/= and then the rest when we got back home after selling some eats. As for the money to buy medicine, I would call someone from my home village to lend me because at first, I was told it was 20000/= then it was increased to 25000/= then 35000/=.

I: How about his mother?

R: His mother always said she didn’t have money, even bringing him to the hospital, it has always been me. She would always say the money she had was for going to the herbalist. That is what she kept doing.

I: When he was given medicine, was there ever a time he reacted badly to it?

R: When he was first given medicine, he would sleep a lot, and I would get scared and fail to sleep because we stayed in the same house. I spent a week without sleeping while taking care of him, since I didn’t want them to tie him up. It got to a point where I got insomnia.

I: What would make you fail to sleep?

R: Making noise, banging the doors, so it was hard to sleep.

I: Ok. How about your relationship with family? Both yours and his father's.

R: Ah, the father said that since the boy was from his clan, he had to go to the ancestral home to perform some rituals, so I also told him that since the boy was on medication, he could also take him there. I told another son-in-law to call him and tell him to organise transport means to take him, while we also looked for money. Other family members were not so supportive, apart from one nephew who kept helping me bring him to the hospital and lending me money for transport, which I would pay back slowly.

I: So, the relationship with the rest was not good.

R: No, they never cared because even when it came to taking the boy to his father’s home, they did not help and said I would foot the bill, while he also kept taking his medicine.

I: Was your relationship with the people in the neighbourhood hurt by the fact that you had a child with mental illness?

R: As for the neighbours, it was one person who made me pay back the shoes that the boy had picked from their veranda and thrown in the toilet. The boy said he had just bought his shoes at 30000/= but his friends said the shoes were old, so I ended up paying 15000/= but the rest of the neighbours were understanding. Although there was a time he lost his shoes and ended up in another village while looking for them, he picked up shoes and was beaten, but he pleaded not to be killed and said he was staying with Mrs. Ssekamanya. These people called someone from our locality, who told them that he knew him and that he was mentally ill and could have wandered from home. I was informed, so I got a boda boda, and this man helped me pick him. I told them to keep him restrained, if need be, so that he doesn’t wander off again while someone comes to pick him.

I: Are there some challenges faced with family members as a result of this boy being ill?

R: Yes, there is. Some of my step-daughters, including his mother, came and told me that if I had chased him, he would not have destroyed my property. They said I should have chased him because he wasn't my blood, since even his mother wouldn't manage to take care of him, but I told them he was still a child in the home, and I would take care of him. I wanted his mother to see I wasn't a bad person because if I was, I would have chased him since the house I am currently staying in was built by a foundation after the house their late father constructed collapsed just after his death, yet I had very few children.

I: When they told you those things, how did you handle that situation?

R: I ignored them and kept living. I told them I couldn't chase him because it could also happen to one of their own next time, and it wouldn't look good if I chased this one and welcomed theirs, although this boy's mother never liked me. Then they left me. Recently, one of them called, laughing about how I was no longer in a position to watch soaps. The comment came from the one who had bought me the TV and was complaining how their children were no longer watching TV because I wanted to take care of someone's child.

I: Didn't that hurt you in any way? I mean, how do you feel when people talk like that?

R: What will I do when people talk? I just told them, you never know, God will give me.

I: I would like to know how your meeting with the medical personnel at the hospital was. How did you find the medics?

R: I don't have a problem with them since I am a coordinator for the VHTs, and that is why the doctor told me that if it wasn't for me, he was going to refer us to Masaka hospital.

I: So, we take it that the doctors treated you well.

R: Yes.

I: While we are still on the point of the doctors, how did they explain his condition to you after you told them your side?

R: The main issue is that the first time, someone else is the one who prescribed the medicine, but when we came back, the one in-charge is the one who saw us. He carried out an interview just like you are doing, he asked him questions while writing. I was consulted only when the boy had failed to answer. He also asked him about the demographic data, and the boy told him I was the one taking care of him. When the doctor asked about his mother, he said she never cared about him.

I: What I am asking about is after he asked all those questions and you explained to him what he told you the boy was suffering from?

R: He didn't tell me. He just wrote in the book and gave us medicine.

I: Did he tell you about the medicine?

R: The names of the medicines?

I: Yes.

R: No, he just wrote in the book and brought the medicine, then explained how I would give it to him.

I: Did he tell you about the side effects?

R: He told me it would make him weak and also sedate him, and truly, he would sleep, which I wanted because he kept manifesting. There is that pill he told me to give him twice or thrice a week. He told me it was strong and would help him calm down.

I: All that has happened. What do you think your grandson is suffering from? When asked about what he suffering from, how do you answer? How do you understand the illness?

R: In my understanding, the mother says it's a family thing from his father's side and that their demons attack him, so I told them that if it's a family thing, they should take him so that he gets better. They would take him and bring him back, and he would relapse, so I told them I was going to keep giving him the medicine until they decided to take him for their ritual. If the medicine helps, well and good.

I: So, you also accept that it is due to cultural things?

R: It is what they tell me. If I agreed with them, I wouldn't be giving him this medicine, but I said people have illnesses.

I: What do you currently think your grandson is suffering from?

R: Hmm.

I: When asked what Lubega is suffering from, what do you tell them?

R: His mind sorted or a nerve malfunctioned.

I: So, you don't know what he is suffering from.

R: I don't.

I: All you know is that it is mental.

R: Yes.

I: How about if I ask about his medicine?

R: Unless I show them the book, because I don’t know the names.

I: Can you identify it using the colours?

R: I can tell by the colours even when administering it.

I: Have you ever been discriminated against because you have a child with a mental illness or an illness like KK has?

R: I have never experienced it because he is a clean child. They would have discriminated against me if he were dirty, but he is clean. He tells me when he wants to bathe and put his water in the bathroom, he washes his clothes, or I tell him not to repeat the dirty clothes, and I wash them. The only person who discriminated against him was his sister, who didn’t even want him to go to their home for food, but the rest of the people at home were helpful to him.

I: Did you ever get worried leaving him alone at home with other people?

R: I was always very worried because the children would throw sharp garden tools anywhere in the compound. I told them to make sure they kept them in the store and locked it because he could relapse at any moment and kill a neighbour’s child or even someone from home. There was a time he hit his mother with a brick, and when the uncle came to ask him, he tried to hit him too, but he dodged it, so they restrained him. He told them to bring him to my home, which they did.

I: When he comes to your place, he calms down.

R: I handle him well and don’t beat him. His sister even said I am the one who has spoiled him. I told them it was the maltreatment that led him to try and poison them.

I: What do you think other caregivers who have children with the same illness that Karim has, what kind of help would they need to be given?

R: In what regard?

I: In anything could be financial, support.

R: Most especially when it comes to finances, they need to be supported because a child like that needs money, let’s say to buy new clothes, because he threw away so many clothes, and even his appetite increases.

I: How about the medicine?

R: If it were possible for the government to make the injectable medicine free. I spend 35000/= every month yet have no clear job.

I: It is a lot of money.

R: It is not only 35000/= add on the 15000/= I use in transport.

I: How about in terms of care?

R: For the children?

I: No, the caretakers.

R: Yes, they should be supported and encouraged so that they can do what they do with one heart, because when you lack support, sometimes you get overwhelmed and think of abandoning the child.

I: Don't you ever get tired of taking care of a child who is always destroying your property?

R: What can you do if it is your blood? God did not create him like that; the illness just came. Recently, another mentally ill child climbed onto someone's car and jumped on it. This person took this case to the police, and I told them it was okay, yet he is mentally ill. If someone wanted, they could push back the board and repair the car. I told this person to hand over the owner of the car to God because everyone told him there was nowhere, he was going to report a mentally ill child, but they should just take care of him.

I: There is a point you made that KK was taken somewhere. Did you also go there?

R: When his parents took him, they told me his grandparents had their shrines, but the father had never taken him there, yet they were demanding that he. They had to take him to their shrine to be seen and for some rituals to be done, but I wasn't there.

I: When they took him, did you notice a change?

R: They have never taken him.

I: When they took him to the shrine.

R: They have never taken him to the ancestral home.

I: Oh, so that message was from where the mother went for the herbs?

R: Yes. The father also last went home when he was still a young boy before his mother died. Ever since she died, he has never gone back.

I: Ok. Some people, when faced with such problems, run to places of worship like a church.

R: Yes, he has born-again uncles who wanted to come pray for him, but he refused. At some point, the parents had tried to do some rituals, but he went and picked the things and threw them in front of his mother and accused her of buying cultural things, claiming that the father was the one supposed to buy them, and yet the mother had taken over that role. He

threw the money on the road and brought the calabash back home. By the time they did those rituals, the boy had already improved a little, but I think when the demons saw the boy wasn't healing completely, they went back, but I have never done that.

I: So, they didn't take him for prayers.

R: They didn't because he refused.

I: What advice would you give to other caregivers of children with the same illness that Karim has?

R: The advice I would give them is that when they notice that their child is like that, the first thing is to take them to the hospital to get treatment for children like that. Secondly, show them care and do not be harsh because when you do, you just worsen the situation. Even if he does something wrong, pretend you have not noticed and try to advise them like I did mine. His hating his sister to the extent of trying to poison them was because she was harsh.

I: Apart from that, is there any other advice you would give them?

R: The other advice would be to keep taking medicine and giving it to them on time.

I: When you look at the future for a child like Karim, what comes to your mind?

R: Sometimes I think God will heal him, and he gets back to work like he was, because he is growing. I keep praying to Virgin Mary so that he can heal and go back to work because his other grandparents died, and all his father does is drink alcohol.

I: In summary, sometimes, you worry about how he will survive and work.

R: Yes.

I: How about in terms of marriage? Do you ever think of that?

R: When he heals. Sometimes he jokes with his uncles and tells them to bring a car so they can pick his bride. When they ask where she is, he tells them I am organizing everything concerning his marriage.

I: When he says that, don't you get worried?

R: No. I just encouraged him that we should go, and his uncles would buy everything.

I: Apart from encouraging him because that is what you have to do, but I am talking about the time when you are alone, do you think about whether what he has said is possible?

R: Yes, you think about it and wonder whether God will ever help you and heal him, so he gets married, and you leave it all to God.

I: What do you think brought the illness that Karim has?

R: I don’t know. If it is not their cultural things, maybe it is about the 780000/= he says he was demanding from his boss, who could have bewitched him, I do not know.

I: When you look at other children of Karim’s age in the neighbourhood and you see he is having these challenges, are there things you think about?

R: Yes. Sometimes you think and say to yourself that one day your own will also be ok, and sometimes you cry because there is another child in town like Karim who has taken about 2yrs with the illness and people say it's cultural.

I: I would still want to know when boys get to that age, some start getting girlfriends. Has that happened?

R: No, he hadn’t yet.

I: Can you tell me about the most challenging situation you have experienced while taking care of him?

R: It is being destructive because if he just manifests talking uncoordinated things till late in the night, and you end up sleeping at 3:00 am. I would tolerate that, but what hurt was destroying my things, which I have no hope of replacing soon, although I brought an electrician to reconnect the power, but as for the clothes he threw in the toilet, the basins he broke, I have never replaced those. I have never replaced my TV and was told what he spoiled was 120000/=.

I: Is there anything else you have gone through that you would like to share while taking care of him?

R: That is it. Apart from people appreciating me for taking care of a child who isn’t mine, yet his mother does not like me. People wonder how I take care of him better than his own mother.

I: How about the fact that he is now lost?

R: I also worry about that because I have tried looking for him through various people, and they all keep telling me that they will inform me when they see him.

I: Thank you so much. I beg we stop here.

R: Ok, sir.

A QUALITATIVE INTERVIEW ON THE LIVED EXPERIENCE OF CAREGIVER 8 (C8) OF AN ADOLESCENT WITH BIPOLAR DISORDER AT MRRH

Interviewer - I

Respondent - R

I: My name is xx, as I have explained to you, I am a mental health worker and right now I am a student at Mbarara University of Science and Technology, and I am doing a Master's in Nursing, but focusing more on Mental Health. I am looking at parents of children who have a mental illness called Bipolar Affective Disorder or we can explain it as a mental illness which manifest in a child in form of so much depression, or having elevated mood or being irritable, that is what we are going to be talking about but before we go further, I would like to firstly know your name, your age, your religion

R: My name is C8, I am 53 years old, and I am a Catholic

I: What is your marital status?

R: About marriage, I am not married

I: Did you separate? Or. . . .

R: Yes, we separated

I: Ok, what is your highest level of education?

R: I studied up to primary five

I: OK, for how long have you stayed with this child, and what is your relationship with the child?

R: That child is mine, I am the mother

I: You are the mother

R: Yes

I: The child is the last born?

R: Yes

I: How old is the child?

R: He is 19 years old

I: How many children do you have?

R: I have 5 children

I: Among those children, is there any other child who has a mental condition similar to this one?

R: Yes, they are there, they are two

I: They also have this kind of illness

R: They got the illness, but for them, they later recovered

I: Ok, for how long have you stayed with this child?

R: It's about 4 or 5 years, he has been stabilizing and relapsing, as he starts to stabilize, then he relapses

I: But even before the illness was there, you had been staying with him

R: Yes, I have been staying with him

I: Ok, you as a care taker of an adolescent with this kind of mental illness, As i have told you sometimes it may manifest in a child in form of so much depression, or having elevated mood or being irritable, hearing from you may help us to understand very well what it means to take care of an adolescent with this kind of mental illness like the one MM has, on that note, I would like you tell me your experience as you take care of MM.

R: The situation I am going through personally?

I: Yes

R: The situation I am going through is a difficult one because I don't get support from his father. I suspect that he is the one who caused the illness because, as my son was going to start secondary school, he went there to ask for school fees. Instead, his father took him and showed him a piece of land to cultivate and advised him to start cultivating instead of going back to school, from that time, MM started becoming sick. It made me feel so bad, yes, the land is there, but he doesn't want to support it. Sometimes I don't have money to come back for reviews – a reason why sometimes he stops taking medicine, and the illness becomes severe, and then he relapses. There are a lot of things that are hard for me. When he gets excited, he destroys things, sometimes he destroys clothes, tears them, or burns them, and even destroys house property. The hardship I am going through is not getting treatment, because if he were on treatment, he would stabilize.

I: What hinders you from getting treatment?

R: Not getting treatment, what makes him not get treatment is that his father thinks the hospital we are bringing him to is not the right place he is supposed to be brought to

I: What does he think? Where is he supposed to be taken?

R: He thinks he is supposed to be taken to traditional healers, yes

I: He thinks the illness is traditional

R: Yes, and he has ever taken him there when he got the illness, he did not get the illness while he was with me, he got the illness while he had gone to his father to ask him for school fees. When he got the illness, he took him to traditional healers, and he was there for 3 months, and we did not know where they were staying for that period. Someone alerted me that the child wasn't at home, we made some search until we got to know that he was staying at the traditional healer's place, I went there and I picked him from there - it was a tough battle getting him from there

I: Where had he taken him, to traditional healers?

R: Yes, it's where they were staying

I: The traditional healer had admitted him?

R: Yes, it was like an admission, my son told me that he used to be put medicine in the nose, and put in the sun, and even smelling, he no longer has the sense of smell. Then I tried so much and got him, and I brought him to the hospital, and they treated him. What challenges me is not getting treatment; it's the main issue

I: Before the illness started, how was MM behaving?

R: He was behaving well, he was a very good child, he could work, he was very intelligent, but ever since he became sick, when he tries going to school, he relapses. I see that when he goes to school, he is not the same way as after he comes back from school, yes

I: In that period before the illness, how was your relationship with him?

R: Before the illness, the relationship was there; he was an understanding, obedient child, calm, understanding, whatever I could tell him, and he could do it well

I: How did the illness begin? There is where you said that he went to his father's home. What did you see and think that the child has started getting a problem or illness?

R: When he was with his father, they called me on the phone and they told me that he had gotten sick

I: His father's home?

R: Yes

I: It's his father who called you?

R: Yes, and he told me that the child is sick, and that he had been cursed with evil spirits "ekitambo". I asked him if they cursed him with an evil spirit? How did they curse him

with an evil spirit, yet he is coming from home to go to school; he is not in a boarding school? He said yes, he was cursed with an evil spirit. I left it at that. I sometimes think about it, and I fail to understand it as a person because I hear they say when a person has been cursed with an evil spirit, they ask for meat, and I asked him, has he asked you for meat, and he replied ‘Yes’. And I hear they say there are those people who eat human flesh (cannibals) can curse you with an evil spirit, and it makes you sick, like that I just heard that

I: And it also makes you sick, when they also want to eat you too?

R: I do not know, I just heard that

I: Ok, they just called you on the phone

R: Yes

I: Then, after you went and picked him?

R: Yes, I went and picked him, maybe what I have not told you is that that child is the only child to his father, these others are for my legal husband. For him, it was maybe the first time to see that illness, but I had already seen it before. I brought him from that side to the hospital

I: Ok, when you met him, what did you find him doing, which confirmed to you that maybe he was a mental patient?

R: What I found, I found him not in a good state, he was dirty, irritable, but when he saw me, he calmed down he fixed himself well. I told him, Let's go. I'll take you to the hospital to receive treatment. He accepted, we came, and he received treatment

I: So, for you, even when he was sick, your relationship remained good; it was not affected

R: Yes

I: He kept listening to you, whatever you told him

R: Yes

I: What about after bringing him here, when you brought him here

R: He was treated, and he calmed down

I: Ok

R: He is not usually in a bad condition unless there is no money to buy him medicine, when he does not take medicine, that is when he relapses

I: In bringing him here, didn't you bring him here when you were saying, but where I have taken him from, maybe it was better, now where I am taking him, will he get treatment?

R: I did not get it, the reason being he was not the first one to be treated here, I already had other older children, I treated them from here, and they recovered

I: They recovered, and they are no longer taking medicine?

R: Yes

I: Can you tell me about your daily experience in your work as you are taking care of him?

R: My daily work?

I: Your daily experience every day in your work of taking care of your son

R: The situation I am going through, I do not know how to say it, it is not good because I cannot leave him, leaving him home completely, leaving him alone

I: Why is it that you cannot leave him alone?

R: I cannot leave him alone because, as I have told you, illness just comes when he has taken a long time to take medicine, and he relapses, like how it is in this situation now, I had to wait for a Saturday to leave him with his siblings.

I: You get worried

R: I get worried

I: You get worried that he may harm himself or harm other people

R: He may walk and leave home without us knowing where he has gone, like sometime back it was there, but now, and also there is a child he hates so much, even if he does some small mistakes, he hates him

I: It's a child hating him

R: No, it's MM hating that child

I: Is the child yours?

R: He calls MM uncle

I: So, sometimes you fear that he may harm him

R: Yes, he is rude to him

I: And in the same situation of taking care of adolescents with such illness, some face a challenge in finances, or money, what do you say about it?

R: I do not have support, you know we have disagreements with the father, for him, he wants to take him to traditional healers, yet for me, I bring him to this side of the hospital. It's me who takes care of him, when money gets lost, he does not get medicine

I: Are there times, you do not have money and you fail to come?

R: Yes, and sometimes I fail to buy medicine in its right full dose as it is needed

I: For some people, some times when they have such a child, they even fail to get time to do other things because most of the time is spent on taking care of the child. To you, what do you say about that situation in taking care of MM?

R: It's like that, but it comes only in seasons when he has relapsed, but if he has not relapsed, I am not like that, maybe my right hand born dislocated, and I do not have so much work I can do

I: What were you doing when it dislocated?

R: Nothing, I was working in the garden, I got severe pain, they took me to the hospital, did an X-ray, and they told me it was dislocated. It dislocated last year, and up to now, I have never recovered

I: Sometimes, some people find a challenge in these drugs, the ones they give their children, may be bringing them side effects, or sleeping too much, or what? how do you find the medicine while giving it to MM?

R: It makes him sleep, but I take it that if he sleeps, he stabilizes, he gets time to sleep, instead of being there sleepless. I see that the more he sleeps, the better he gets. I see it is good to sleep because he stabilizes when he sleeps too much. I have treated them for some time now. When I see them sleeping too much sometimes, I tell health workers, and they change the kind of drugs they take, like that

I: There is where you talked about the relationship between you and his father, and you showed that that relationship is not good, isn't it? I would like to know MM getting illness is the one that brought the change in the relationship, or the relationship was not good even before MM became sick?

R: We had already separated even before the illness; it's not the illness that caused the separation

I: And when the child's illness came in, it worsened the situation?

R: Yes

I: Ok, so he thought there is something you are doing to the child or. . .

R: I also do not know

I: Apart from your relationship with his father not being good, are there other family members whose relationship with you has gotten a problem because of MM's illness

R: No

I: What about your relationship with people in the community where you stay

R: I have a good relationship, it has not changed anything because, although MM sometimes leaves the house and just walks away, he has never destroyed people's property or abused a neighbor's child, or beaten them. But because people know that when a person gets that illness, it makes them do different things, some others get scared.

I: They fear because he is sick?

R: They fear that because he is sick, maybe he may sometime cause harm to them, but he has never done anything harmful

I: What about threatening their children

R: No, he does not threaten them

I: I mean, community members, telling their children not to play with him, that he is mentally sick

R: No, that did not happen

I: It did not happen. What about the issue of the father, sometimes disagreeing with you, especially on where to take the child for treatment? firstly, I want to ask, when he took the child to traditional healers, did you see any difference?

R: No difference, it just became severe, but because he believed in it, he just remained there

I: If you weigh it on a scale

R: There is nothing that changed; it only became severe, even though the patient never wanted to be taken there, he was in disagreement, most of the time, he wanted to run away from there

I: Ok, in that situation, you said you were not in agreement on where to take the child, how did you manage to handle that?

R: We are there; we are there. I have nothing to do, we are there, can you die because of that? When I get money, I treat him; when I fail to get money, he remains there, but I try

to manage, sometimes his elder siblings help me, send me some money to take care of him

I: MM's siblings

R: Yes

I: Are they older?

R: Yes

I: Are they working?

R: Yes, that is how I try to manage

I: They treat him well? Though they do not have the same biological father

R: They love him so much. MM is a calm person, only that he is sick; they love him so much

I: Ok, what about when you came to the hospital, how was the situation when you brought MM to the hospital?

R: The situation that was there

I: Yes

R: The situation, mine? Patient's or the health workers?

I: That of the patient and yours

R: Patient's situation and mine

I: Yes

R: There was no situation that was there when he was treated; he calmed down, and he was well

I: When I tell you to talk about health workers, what can you say?

R: Health workers

I: Yes, how they treated you

R: For health workers, according to the period I have treated here, I have nothing to say about them; they are good. They treated us, and when they wrote for me the drugs to buy, I bought them, and my patient improved.

I: Some complain and say health workers were a bit rude, they did this and that, for you when you brought him, what was there?

R: No, maybe, the time he had spent without taking medicine, they were a bit rude, but it's the truth, he was not supposed to stop taking medicine, because the health workers did

not tell him to stop taking it. Even the others stopped taking it without my awareness, because they were adults, and I had nothing to do

I: But in this situation of medicine, he receives here, in your view, what do you say about it, does it help? how does it treat him?

R: They do not give it to him, they write it for him, and we buy it

I: Aren't there times when you find drugs available?

R: There are times when it's there, but I have taken long without finding it available

I: Most of the time, when you come, it is not in stock?

R: Yes

I: Then you go and buy it. What about in Kalisizo?

R: In Kalisizo, it's not always there

I: After this, I will connect you to the health worker in that hospital to see whether you can get medicine from there, if they have it, then you will get it, it is even nearer for you

R: I went there once, and it was not there. From that time, I have never been back there

I: Sometimes they have it, have you ever experienced a situation of being segregated or being despised because you have a son with mental illness?

R: Segregation happens, I can say even if you are not sick, and some people segregate, but I think it's not because of illness

I: You think it's because of what?

R: I think it's because of poverty, when you are poor and you are not on the same financial level as them

I: And they segregate you? Even those who are poorer than you can segregate you?

R: The one you are richer than?

I: Yes

R: It's hard, no, it's hard

I: So, the situation of segregation has been there

R: It has been there and it's there, but I do not relate it to the illness, that it was due to the illness

I: What did you see and suspect that there may be a situation of segregation?

R: What I see and suspect is that there may be segregation. Let’s say there may be a ceremony and they do not tell me, or there is something, and I just hear that there was something, without you knowing, isn’t that segregation? Yet I am also in the same village

I: Even if it’s a ceremony, you would have given them a bunch of bananas

R: The person may come and ask you for a bunch of bananas, but they do not want you to go to their ceremony. Many come they ask you for a bunch of bananas, but they do not call you on their function. For me, when you come and tell me that you have a function and you want some food, have you invited me? No, I just give you my banana, and I give it to you, and I do not come, you have not told me about when your ceremony is to take place. It is like someone tells you only wanting food from you, I give it to them, and that ends there

I: So, when you realized that the situation was there, how did you manage it?

R: Nothing to do about it because it requires money when I do not have it, nothing to do, I just remain in that situation because when someone invites me, they are supposed to give me a card but if they don’t, you just remain home. It shows that they have come because of food but they do not want to see you on their ceremony

I: So, for you, you say caretakers should be supported like giving them some money so that they can provide such things to their patients, is that what you mean?

R: Yes

I: Ok, what else?

R: That’s it

I: What about counseling?

R: Counseling

I: Yes, what do you say about counseling the caretakers

R: It is also an important issue

I: Why is it important?

R: Because it is important, a patient can calm down, especially when the caretaker is not part of a family member, they can get a big difference

I: Ok, there is counseling a patient and there is also counseling a caretaker, like apart from bringing MM, they see him, they talk to you about him, but you, as a caretaker. They also

counsel you or encourage you not to get worried, they say this and that, that is what I am asking about, what do you say about that? Counseling, on the side of the caretaker?

R: Counseling the caretaker?

I: Yes, you think it's an important issue?

R: It's also an important issue, but it is rare it's is rare

I: So, in your thinking, health workers should be doing it or not?

R: Yes, they should be doing it

I: Why do you think they should be doing it?

R: Because that illness is not easy

I: What do you mean by saying it's not easy?

R: That's what they are supposed to do because all the trust is towards the health worker, so any word they say in a way of counseling encourages, it builds, and brings back life and hope

I: Ok, what about when it comes to drugs, what kind of help should be given to caretakers, especially when it comes to medicine, what do you say about it?

R: For that, when it comes to drugs, the health workers also don't have the drugs, if they do not get it, what should they do? They also have nothing to do when medicine is not given to them to be given to patients, they have nothing to do about it

I: But in the right way, how should it be?

R: Medicine should be there, but because they are not the ones supposed to buy it and put it in the hospital, they are also workers, they do what they are supposed to be doing

I: You talked about taking the child to traditional healers. When some people get problems like this, sometimes they take them to churches for prayers. I would like to know what you say about prayer, trusting God?

R: On my side or the patient?

I: On your side as a caretaker, because it's you looking for his treatment

R: Me and the patient dedicated ourselves to God's hands, and we have been praying. MM was even serving in the church, but when he relapses, he stops going to church, and for prayers, we pray so much

I: You think there is a way it helps

R: Yes, it helps. Every time you are hoping, you have hope from God

I: And whenever you trust God, what happens?

R: When we trust God, what happens?

I: Yes

R: The situation changes, and you cannot remain the same

I: Ok, I would like you to tell me, sometimes when you think about the future, when looking at MM, what do you think?

R: The future?

I: Yes

R: As me?

I: Thinking for MM, because you are the care taker, he is still young, as any parent, if you have a child sometimes you say, I want my child to be this, to do this, what do you think for him, considering that he has this kind of mental illness?

R: For me, all the time, I hope he recovers and goes to the garage and learns because he wants to learn motor vehicle repair. And I have ever taken him there, after paying their money, I was left without any more money, then he relapsed

I: Ok, what I would like to know is, are there times when you get worried that even he may fail to do any work because of illness?

R: Yes, it’s possible because I see it, he does not have energy well, as I told you that when he works, you see that he is weak, yet I think he may recover

I: He is a boy. Do you sometimes think about marriage when you picture that in the future?

R: About marriage, that is not the most important thing; the most important thing is life

I: You do not get worried about it and say, maybe my son will be able to grow, what about grandchildren?

R: Yes, and he becomes independent, I think, in a way that at any time, he will recover, so that he goes to learn vehicle repairing because school stopped there

I: School stopped there because of the illness or anything else

R: Because of illness

I: He got the illness when he was in which class?

R: He was in senior two

I: Those symptoms manifested when he was also still at school, or?

R: Yes

I: The school administration came and told to you or they told his father?

R: No, for him, those symptoms manifests when he could go to school feeling weak, and he sleeps and cannot go to school because he comes from home to school, and he cannot go to school, just sleeping for days, not going to school

I: When you came here, did you find other sick people worse than even how he was at that time?

R: Yes, they were admitted

I: When you saw them, what came into your mind?

R: What came to my mind was that they delayed bringing them here for treatment

I: Didn't you get worried that maybe even my son may be like that?

R: No

I: Ok, now, if I ask you to explain the kind of illness MM has, how can you explain it? Right now, what kind of illness does he have, in your words?

R: In my words, he has a mental illness

I: All these people here have mental illness, but what kind of illness?

R: Illness of the brain, the illness is in the brain, in understanding

I: What I want to ask is that when you brought him here, after health workers saw him, what did they explain to you he was suffering from?

R: They did not explain to me

I: What about when we talk about the drugs he takes, which medicine does he take?

R: I do not know it

I: They also did not explain it to you?

R: They did not tell me the name; they just wrote it for me to buy. I do not know it, they just told me how it is supposed to be taken.

I: Apart from telling you how he is supposed to take it, sometimes they tell you, you can give a person this medicine and it does this and that, did they explain it to you?

R: Yes

I: Or maybe when he takes this drug, he will get this and that

R: Yes

I: What did they tell you the drugs do? They told you it may do what?

R: Saliva drooling, I see that situation

I: When you look at MM, apart from saying that his father refused him to study and he gave him land, it's the reason you are giving, what you think may have caused the illness. Is there any reason?

R: No

I: That's what you think?

R: Yes

I: When he got the illness, didn't you think that even his siblings, the other two children you had already had the illness, so it may not be so surprising?

R: Yes, they also got the illness, they also had their reasons, as individuals, like MM had his, that is what I thought, that they also have their reasons, which they experienced, and it affected their brain

I: But in the family, are there people who have suffered from mental illness?

R: Yes, even me, their mother, I have ever had the illness

I: What did you use to treat it?

R: I was also treated, I do not know what they treated me with, they never brought me here to the hospital. Maybe my older siblings went somewhere, she did not bring any medicine, but I just saw myself recovering, I cannot tell you that they smeared medicine on me, or it was given to me to drink, but I recovered

I: Sometimes, when some children reach a certain age, like MM's age, you may find them with girlfriends. Have you seen such in MM?

R: No

I: When you think about it, don't you get worried, considering the condition in which he is?

R: Getting worried about what?

I: To say that if he starts getting girlfriends and all that

R: No, I am not worried about it

I: What about looking at other children in the same age groups as MM, let's say in the village, when they are going to school, let's say, when life is moving on well, how do you feel?

R: I do not feel anything because it is God who created us like that, and we cannot be on the same level. You have nothing to do about it, and also, you go with what you are

I: I would like you to give me an example of one experience that has been the most challenging while taking care of MM?

R: The experience I have had while taking care of him is not getting treatment, all the time I am just going backwards, I do not go forward

I: What do you mean by saying going backwards?

R: What I mean, a lot of money goes into the illness, yet he is not the only one who gets sick; even others become sick, but his sickness is usually very strong, it scares me

I: What message can you give caretakers of adolescents with this kind of illness, like the one MM has?

R: The message I give them is to trust God and to pray to Him because He is the owner of all, whichever kind of situation, what you have to do is to just pray, you will see later that life moves on

I: This is the first thing, the second one, what message do you give to them, except that of trusting in God?

R: To take care of their children, to treat them, and nothing else

I: What about those who would want to first go to traditional healers?

R: That one, there is nothing good, there is nothing good from there. From the beginning, they are there to make money; they just make money, but you, as a caretaker of the patient, cannot even have mercy on them; they just have to ask for money from you. MM spent in a shrine 3 month with traditional healers, it was going to the 4th month, I did not see anything good that he came out with, but he just continued getting worse. I saw that his life changed; he lost his sense of smell because of the way/method they were using to treat him.

I: Up to now, he still does not smell, it is still there, or has it started coming back?

R: I have not checked him now, but I think he has not yet recovered his sense of smell

I: As we conclude, anything you would like to ask about what we have talked about? I am going to answer the question you first asked

R: What I am asking, like when you asked what MM is suffering from and I told you mental illness, brain is sick, but for me I say that your health workers separate them like the when you say this one has malaria, malaria brings headaches, it brings fever, even

stomach ache can bring headache. What I am asking myself is that is MM is suffering from fever or just brain disease. Now I do not know the true illness he is suffering from

I: Thank you so much for asking that question. I request to add it to the other, I request to first stop the recorder here, we stop here, thank you so much

A QUALITATIVE INTERVIEW ON THE LIVED EXPERIENCE OF CAREGIVER 9 (C9) OF AN ADOLESCENT WITH BIPOLAR DISORDER AT MRRH

Interviewer - I

Respondent – R

I: As I have explained to you, my name is xx, I am a Mental Health Worker, but right now I am a student at Mbarara University of Science and Technology, and I am doing a Master's in Nursing, but focusing more on Mental Health conditions. I am doing research among caretakers of adolescents who have a kind of Mental illness like the one your child has. I will be asking you questions concerning your experience as you take care of him. I will be asking you some questions as I have explained to you, but if there is anything you have not understood, you are free to tell me that there elaborate I have not understood so that we can be together. Our interview is going to take between 30-45 minutes, and we will be done. Before we go further, I would like to first ask your name

R: My name is C9

I: How old are you?

R: I am 43 years old

I: Your gender?

R: I am female

I: Your religion?

R: I am a Muslim

I: Muslim

R: Yes

I: What is your marital status?

R: I am not married, sir

I: You divorced? What happened?

R: When the father of that child died, I got another man, I had a child with him, my last born, but I never got married to him, I am not with him

I: Ok, the father of the child died?

R: The father of OO died when the OO was 2 months old

I: Sorry, how old is your child, your last born?

R: 5 years

I: Ok, but in total, how many children do you have?

R: In total, there are 7 children. I have a pair of twins among them, sir

I: Ok, out of the 7 children, is the last born the only one who has a different father from the older ones?

R: No, sir, from him, I had 3 children, 2 girls, and the sick one is the last one from that first husband who died

I: You studied up to what level?

R: I stopped in Primary three

I: In primary three

R: Yes

I: I would like to know, you have told me he is your son, for how long have you stayed with him?

R: I have stayed with him ever since his father died, and he reached 8 years, he started going to boarding school, it was the daughter to his aunt's daughter paying school fees for him

I: Which class did he start attending boarding school?

R: He was in primary five

I: How old is he?

R: He is 17 years old

I: Ok, now, you as a care taker of an adolescent with this kind of mental illness which sometimes manifest in form of so much depression, or having elevated mood or being irritable which is called Bipolar Affective Disorder in English, hearing from you may help us to understand very well what it means to take care of an adolescent with this kind of mental illness like the one your son has. On that note, we would like you to tell us your experience as you take care of your son

R: Ok sir, that child needs to be given a lot of time, listening to him, when you disagree with him, he becomes angry and he wants to make a fist and box you, but when you give him time and you listen to him, and you show him that what he is doing whether it is bad, you do not abuse him, you do not beat him, he is happy, and you can treat him but if you are rude, you cannot treat him

I: Ok, you, as a caretaker, what has been your experience?

R: My experience? I have experienced several situations, there is spending a night when he is singing, spending a night reading prayers of his Catholic religion, waking up at night, like at 2 pm, and he starts eating and eating a lot and drinking. There are times when he refuses to eat food, and he does not eat anything at all, and I have to give him something to drink, and he drinks.

I: Ok, what else have you experienced?

R: What I have experienced, one time he went to school and he came from school, and I waited for him up to 10 pm

I: Which class was he in at that time?

R: Senior three, that is when he became so irritable

I: You waited for him, but he was not coming back, up to what time?

R: Yes, by 10 pm, he was not yet at home I was looking for him, but I had not seen him. As it was getting to 11 pm, he came back and banged on the door. He busted out loud that you open the door, I woke up and opened. I looked at him and his eyes had tears, the uniform shirt was in the bag and he was in sleeveless and he asked me whether there was food, I told him yes, he told me should I eat some, I said yes, after eating he asked whether there was drinking water I told him, there wasn't he banged the door and went out and disappeared. I looked for him, but I could not see him. It reached 11 am, his older sibling, the one he followed, called me on the phone and told me that he was at the Police Station, but the sibling was at school. I asked which Police Station? He wasn't sure, but he thought maybe Lukaya, but he gave me the Police Officer's phone number. When I called, the Police Officer told me that your son is in Mukoko. I went the next day, as the previous day I lacked money. I had to talk to his Auntie, who is in Tororo, she is the one who sent me shillings 20,000 for transport to Mukoko. When I boarded to Mukoko, I found him there, The Police Officer said that this child destroyed our property. They had

put him in a room, but he destroyed property in there, including a bucket. I told them to tie him with handcuffs, they tied him with handcuffs, I bought water and chapatti, and I fed him as he was tied with handcuffs, but they did not tie him so tightly. He untied himself and started beating the window and the room. The Police Officers went to catch him, there was a sergeant Police Officer whom I didn't know by name, and a Corporal; he bit the Corporal. They called other Police Officers and tied him with handcuffs, and I bought 2 ropes and they tied him with those ropes too, but he started shouting from there. Corporal said requested me to provide 5000 shillings to test my son for HIV. I provided the money; a health worker came and took off a sample and tested him – he was found negative. His Auntie had suggested that we take him to Butabika hospital, but we did not have the money to take him there. The sergeant said no, in Mulago Masaka, there is a ward for Mental patients, so he said look for 50,000 shs. I had to wait from morning up to 6 pm in the evening. The people at their home in Tororo are the ones who got this money, and we brought him here using the police 999 patrol car. I was here with him, then I could leave at 10 pm because I had to go home where I stayed, I had a young child, and even here he disappeared, that period was rainy season, maybe what he had, he had phone contacts in his head

I: So, what caused you to leave at night was because you had no one at home who could be there for you here or be there for you at home?

R: Yes, I had no one to be there for me here, I had no one to be there for me at home, so I used to wake up very early in the morning to come, then he disappeared from here

I: He escaped when you were not around

R: He escaped when I was not around, when it was raining, he had those phone contacts in his memory, but he did not know mine. So, when he reached outside, he gave out the phone number of the daughter to his Auntie who pays his school fees, but he could go asking for help on mobile money, reading out that number so that they call on it that he wanted some money, that at school there is some where they are going to tour. So, he kept walking. Finally, the help he got, he got it from a lady on mobile money in Nabingo, as you go to Kampala, that is the lady who helped him

I: He travelled from Masaka to Nabingo?

R: Yes, he once left my home where I stay in Masansa, it is also in Masaka, but walking a distance to reach Mukoko, it is also a long distance. While I was there, the person who pays his school fees had the phone number of his Head Master, so she called the headmaster, she asked if the child was at school, and he said he had not seen him for long time without seeing him, even in exams he did only 3 exams. The headmaster tried to look for me, and he reached me where I was, and he asked me for the child. I told him you found me dressing up, going, the child is in Mulago, and he asked, 'Why didn't you tell me about it or send someone to tell me?'. I informed him that I did not have anyone to send, and I told him that I had the news that he escaped from the hospital. The headmaster took me in the car, he drove me and kept calling the mobile money lady and he told the mobile money that keep him for us and that he is not a Mental patient, he is a student, and we reached there, we found him there. Headmaster asked me What have you decided to do? I told him what I see is that I am not taking him back to Mulago hospital, there is no way I am taking him back to Mulago hospital. The headmaster said, I am also in a hurry, I am not taking him to Mulago. So, I called the son to his Auntie, he came from Kampala and sat with him in tax and he took him to Butabika hospital, but he had ever taken him there, so this was about the 3rd time but in Butabika, when they took him there, they had said we should go back in January on 2nd. I did not get transport to take him back, but he kept going to school. He started slowly by slowly, he first lost weight became slim on face in one week, the second week, bones could be seen on his skin, I kept on seeing things he was doing, I could try so much to calm him down, if I was easily angered, he would have caused harm to me, I used to calm him down

I: Ok, thank you for explaining. Among his family members, is there any Mental patient?

R: I have never seen anyone

I: What about on your side?

R: In my family, there is one, the older sibling to my father, is the one who has a son with Mental illness, yes, the older sibling to my father, his son

I: Ok, before he got the Mental illness, how was he? If they tell you to talk about him before he got the illness, what will you say?

R: He was a very good child, he was disciplined, he loved to study and he was intelligent and he also wanted so much to stay with people of his tribal language. He did not want to stay with me so much, he wanted to stay with people of his tribal language

I: The illness came in

R: The illness came in, he picked up his Auntie's phone, threw it in the toilet, and after throwing it in the toilet, they picked it from there. She realized the things he was doing were not normal, so she requested that I be with him for the remaining part of the holiday.

I: Where was his Auntie?

R: He was staying in Tororo, she sent me transport to go and pick him, he started those behaviors, I called the son to his Auntie, he took him to Butabika hospital the first time

I: When you saw him, you said, This is Mental illness?

R: I said this is Mental illness, so where I stay there is a Health facility Kiyumba, I thought that medicine was there because it was up graded to a health centre four, I went there, they told me it's not there, I have to go to Masaka. I instead took him to Butabika hospital, he was there for 3 months, and he was later discharged, but they had told us to take him back on 2nd January, we did not go back because we lacked money later while studying, he relapsed

I: He was not taking his medicine

R: He was not taking it because they said he had recovered, but on January 2nd, he must go back

I: But they said he had stabilized, and they gave you medicine and to come back for a refill

R: Yes, he took the medicine, so when I reached there, by the time they called me to pick him up so that he could go back to school, he no longer had medicine to swallow. So, his medical form showed a return date of 2nd January, but I brought him this side (home), and he went back to school. He performs well in school. When he stabilizes, he is the kind of person who does everything for me. We fetch water from afar, but OO goes and brings it for me

I: Now that the illness is there, what is your relationship with him?

R: He still related with me well but whenever I could try to disagree with him, he could become angry, one time I told him Junior you will kill me, he said mother it's you who is

going to kill me, why is it that when I tell you this, you disagree with it? He does not want to disagree with him, so whenever I could tell him something and he refuses, I keep quiet, I leave him, but later, after calming down, he does it, but when he escaped and crossed to the other side, we took him to Butabika. I feared bringing him back to Masaka because he might harm me

I: You feared?

R: I feared him because the time I spent with him here, he used to be irritable, and I told the doctor, and he was not sleeping, but they gave him some medicine

I: Ok, I would like you to explain to me about your daily work as you take care of him, before he was taken to Butabika hospital

R: That child, where I stay in the community I dig for people in their gardens as my source of income, if someone has a work, I go and dig for them. At first I used to leave him home during holidays, but later I said why should not I go with this child, I started going and working with him, we used to work and he tells me I am tired and I tell him to go back home and prepare tea, I find him sleeping and he tells me he does not have energy, today I do not have energy, I leave him and I do my work, and I call him sometimes to take tea. Sometimes he tells me he will not take it, sometimes he tells me he will only drink water

I: Which challenge have you experienced in your daily work as you are taking care of him?

R: Taking care of him, what has challenged me is mostly what to eat. For school, he was sorted, the person who has been taking him to school has been giving him everything, and sending his school fees to school in time. He refused to join vocational work and insisted on studying, and luckily, they were willing to support him. Yes, only what to eat because he can be there and he tells me, you know what, I want greens, but the greens he is asking for, he wants carrots in it, green paper in it, such things I cannot afford to get for him. There was a time it was Easter, we bought some meat, he said he will not eat it, he said he did not want to eat it, he said he wanted only greens, and he said he can prepare it for himself, but where I stay I cannot get it, and if I try to get it, I can only get cabbage, but I cannot get the rest, there he refuses to eat food, he says I will not eat

I: When he refuses to eat food, how do you feel as a mother?

R: When he refused to eat, I used to ask him what he wants and he tells me, he says you will buy for me passion fruit, I send his young sibling, he brings it for him, but if he says he will not eat that sauce, he will not eat it, he may say if it is matooke, I do not want it, I want millet, I want cassava

I: Home food

R: Yes

I: I call his Auntie and he says where are you going to get millet, I say I do not know, but I try I buy millet and cassava floor and I mix it, and I prepare it for him

R: Most people get a challenge in preparing it

R: No, for me, I stayed there for 15 years and I had 3 children, I do not have any problem in preparing it

I: Ok, apart from a challenge in money, he has asked for eats, there is no money to buy it as you have said, which other challenges have you experienced?

R: No other challenge

I: Some people find a challenge, they find themselves all the time, like you have said you do dig for others in their gardens as your source of income, but when the child is sick you find that all the time is spent on the child, the time you would have used to do other things, what do you say about that?

R: This child, the good thing is that even though he has been sick and he has spent time without taking medicine, I have been able to go with him wherever I go to work, and he listens to me and when he has refused to dig, he just sits there, and we come back together or to tell him to go back home. Sometimes when he refuses to dig, he has to keep waiting for me there. I normally do this to prevent him from disturbing the neighbors.

I: Apart from that, what about a challenge in transportation? They have told you to come back to the hospital, you said even going back to Butabika, you failed to get transport, what about here in Masaka, have you ever failed?

R: Here in Masaka, I have ever walked on foot many times

I: How many miles did you walk on foot?

R: Because where I stay, you have to pay 5000 shs to get to Nyendo

I: That’s far

R: Yes, because when we board our usual cars to come to Nyendo park, it's 4000 shs, but I used to get up very early in the morning, and it was the rainy season, sometimes I could find merciful people who knew me, and they gave me a lift. You tell someone your problem, you later see them taking you to the hospital, because I reached a time when I was getting medicine from this side

I: What about a challenge in finances, like in buying medicine, you used to get all the medicine?

R: I will not lie to you that health workers have ever told me to buy him medicine, all the drugs were given to us, and the doctor used to call us to bring the patients for medicine, and he would ask, How is he? Does he sleep? Does he eat well? So, what I explain to him is about the medicine he gives me, I did not buy any medicine

I: Some get it, but they get disturbed by side effects, and they say this drug makes my child sleep so much, makes the child get saliva drooling, it does this and that, has that ever happened?

R: Mine did not have saliva drooling, but it reached a point and he was not sleeping, and when he gave him the medicine, he could go long without waking up

I: According to what you have told me, it shows that your relationship with you and your relatives is not bad, considering that you have talked about Auntie, I do not know whether you have had any challenges relating to other relatives

R: No, it's not bad, because I will not lie to you, among those 3 children, there is no child I have paid school fees for, they have been paying for them school fees. One is studying Nursing, while the other one has been studying at Nakawa Technical Institute. She finished she will be graduating with her degree next year

I: That is good news

R: Yes, on the side of their late father, their aunt has tried so much, she now feels so bad to see this one in such a condition

I:` What about on your side?

R: No, the truth is on my side; there has not been any support to help these children

I: A person may not help you, but they wish you well. One may want to help you, but they do not have, but there is one not treating you well, when the relationship is bad

R: Maybe when I was at the hospital, my father used to send me some small money, and he could send me 10,000 shs, and send me 5000 shs like that

I: Ok, what about your relationship with the people in the community where you stay, what do you say about it?

R: Community members where I stay, they love me so much, and they love Junior so much. They did not know his real name as OO except at his school. They say he is a disciplined and quiet child, they love us so much, and those greens, some of them have been the ones giving them to me for free

I: So, you do not have any bad relationships?

R: I do not

I: Is there any situation that has challenged you mentally as you are taking care of your child with Mental illness?

R: No, the situation I have been experiencing is when there is nothing to eat. There was a time I reached when getting what to eat was hard, and I said What am I going to do today. But when I called his Auntie, she sent me some money, and I bought flour

I: Ok, have you ever been discriminated against because you have a child with a Mental illness?

R: No

I: Like some people starting to talk about you in the village

R: About talking, that one cannot miss but Junior is a person who has not been showing so much that he is a Mental patient, the medicine he takes, he has not been drooling saliva, no, only because we got a challenge not going back to the hospital on the dates, they told us and bringing him here he was very excited

I: Right now, if they ask you, where is he? What do you tell them

R: Who?

I: Village members or your friends

R: I tell them he is in Butabika hospital

I: Ok

R: And they ask me whether I hear from him, I tell them I hear from him, but not so much

I: So, they are there for you

R: Yes, they ask me, but they cannot help me so much; they just want to know

I: Ok, in your thinking, what kind of help should be given to caregivers of adolescents with this kind of illness, like the illness Junior has?

R: Caretakers? When are they the ones to get help?

I: Yes, what kind of help should they be given to caretakers like you?

R: Care takers should be advised not to be too rude to patients and the caretakers also need food since the medicines make those patients eat and drink a lot. Good enough when on the ward here, food is provided by the hospital. I used to get it for him sometimes when I had not cooked, when he had stabilized and understood, he used to also get it for himself, as I also prepare other foods.

I: It is still given

R: It is still given

I: Yes, you heard that trolley was moving around, they were serving porridge

R: And porridge, we also used to get it, so for you, as the mother, you just add on what has been served, yes

I: What about counseling? What do you say about it? Do these caretakers also need counseling?

R: I think it's the patient who needs to be counseled because there are times when they are understanding and they need some counseling, and there a times when they are understanding and they need to be taught

I: What about the caretaker?

R: For caretakers, what I saw the time I spent here was that every caretaker was caring about their patient and I also saw that there were merciful caretakers, they could take care of theirs and also taking care of yours, as you have gone to wash, they come and tell you, your patient is like this and that

I: What about things like clothes and all that, is it an important help to caretakers?

R: About clothes, it is not bad, but mostly the helpers need to go to the patient because those patients throw away their clothes. for me, within one day, OO threw away his clothes, shoes, and school bag - I have never seen any of them again

I: You do not know where he threw them

R: I do not know, he repeated it, they gave, he took the remaining clothes, and he gave them to someone, the trousers and the shirt

I: Yet he did not have much

R: He did not have much, he had few clothe but he gave them away and he remained with the inner trouser, he gave out the clothes, they give, even here, there was a patient who took his traditional clothe (Kanzu)and gave it to Junior but for me after some time I removed it from Junior, he had already put it on. I removed it from him and took it back to his mother, but he abused his mother so much because he thought that whatever he gave, the mother would go and take it back. most of them give so much

I: We have talked about modern treatment in hospitals, but sometimes, some caretakers, when their children start behaving in a way they do not understand, they first go to other places. I would like to know how you manage that?

R: Going to other places?

I: Yes

R: No, I refused. Why? I first looked and said what made my son reach to this point was chemical

I: Which chemical?

R: It was a chemical, he used Marijuana, he used it when he was young, and it spoilt the brain. I thought about it and said the truth is when a person uses such things, there is no witch bewitching him. When I was staying with him after discharge, he told me that in Butabika, they have been teaching and counseling them. The doctor said that it was they who caused the illness to themselves when they were seeing. I did not have so much doubt in that because the truth is, a person who uses these substances gets Mental illness, and for a young child, it is worse

I: So, you did not go to other places?

R: No, and I asked him, Junior, when did you start using those substances? He told me in primary five

I: Using marijuana?

R: Yes, and he told me it was coming from outside, and he used to put it in tea; it’s his friends who taught him. And he told me that the doctor in Butabika said that if they stopped using it and took their medicine well, they would recover. Is it true?

I: It’s true, some go for prayers? what do you say about that?

R: Prayers? prayers would not have been bad, but it is good as a parent to know the condition of your child, you understand that this child is mentally sick, but what was the cause? First, think about it in your heart

I: I would like you to tell me, when you brought him here, did you find other patients who were in a worse condition than he was in?

R: When I brought him? All those who were there and those who were in a worse condition saw that mine was the one in the worst condition

I: What I am trying to say is, when you found other patients, somewhere onwards, the way they looked, didn't you get worried?

R: No, it's mine who was in a worse condition, because even the way we brought him, the doctors quarreled with the Police Officers, but the Police Officers had nothing to do, he was tied with hand cuffs, he was tied with 2 ropes, the doctor we found here quarreled so much why he was tied like that, I told the doctor that this patient was fighting, Police Officers did not say any other word, but it's my son who was badly off. Among those I found in the ward, mine was the one in the worst condition. I also found one who used to sleep too much; he could sleep, and it came to morning, but he could not wake up the following morning

I: Apart from chemical, as you have said, or marijuana, what else do you think caused illness to Junior?

R: Groups, groups are the ones that brought illness to Junior

I: How?

R: When Junior got his friends, and for you as a parent, even if you talked to Junior, he could not listen, there is a time when he came and told me that he is going to plait hair, and I told him, I your mother I am in a rental, if you plait hair, rent a house, he had gotten a group which had plaited hair, but I did not see anything else, its groups

I: Ok, sometimes when you look at adolescents, especially those who are in the same age group as Junior, in the same age group as Francis, the ones he has been with, what comes to your mind as a parent?

R: The one he has been studying with?

I: Those in his age bracket, and you see them, they are doing something

R: I also think and say that I wish mine also recovers and his brain comes back like these, but when I see a child using marijuana like OO I feel bad in my heart

I: You see that they are heading in the same direction

R: Now I know Junior will never recover and be like how he used to be

I: Why?

R: I do not think

I: Why?

R: Will he stop using those substances? Because in Butabika, they said he was supposed to be there for 9 months, and if he did not escape from here, we would have still been there

I: So, you do not think that he can stop using them?

R: I do not think, maybe if he has gone somewhere for several months, but I do not know, Junior, to stop using those things? Because he has been attending school, and I saw as if he had stopped using them but yet he was getting time and leaves school very fast and joins the group, and he shows me that he has gotten those substances but has not used them, and he comes back home. While I am sleeping, he gets out of the house and starts using them. Sometimes I wake him up in the morning and I ask him whether he will go to school and he says Today I am not feeling well, I am not feeling well, and I leave him

I: Sometimes when children reach those ages, they start getting girlfriends, if it's a girl, they get boyfriends

R: No, Junior does not have that, and one time he told me that in 5 years, you are going to see grandchildren from your daughters. I asked him; Are you going you be a Catholic priest? He said No, do not expect my child

I: When you look at the future as a caretaker, what comes to you especially as regards Francis?

R: On OO? I see that he does not have a future

I: Why?

R: He has been playing with his future, the one who pays his school fees, this is the third time he has been playing with her money. This last term, he did only three exams and then he became excited, and there is a school where she was paying school fees for him, he studied one weeks in that school, he has been playing in his future, right now, he does not have any other future, chances are no longer there

I: When you think about work, and say which job my son will do, do you think about it?

R: I do not think there is any work he can do in future

I: Why are you losing all hope?

R: I see him, may be, may be as the health workers do their role, may be if he recovers, but there is no work he cannot do, no, no work he cannot do, only that he puts it to himself that he wants only one thing, to continue with his education

I: What about education, what do you say about it? Sometimes, don't you get worried about his academics, especially since he is sick?

R: The other Headmaster had learnt this child, you know he got the illness while at school, and he beat up children who were playing football, and they beat him. The headmaster called him and he told him, You wash your uniform. The headmaster had already studied him, so when he washed it and put it on while it was wet, he came back home. The following morning he went back to school there was an exam, he removes the shirt, remains in sleeveless, he enters to do an exam, the teacher tells him you will not do the exam get out, they went and told the Headmaster, when they Headmaster came, he told the teacher to leave him to do an exam, and he did it so well, so the Headmaster told me that he knew Owori's behaviors, even at school, Owori loved him the most, only the Headmaster

I: On the same issue, about the future, do you think about his future concerning marrying, or do you not think about it?

R: I think about it, but for him, for the time I have spent with him, he does not think about it

I: When you think about it, sometimes you get worried?

R: Yes, I say he may not have a child on earth, but for him, he has never thought about it

I: Can you tell me the most challenging situation you have experienced while you taking care of this child?

R: I have failed to understand that one

I: What I am saying, in taking care of him, is there a situation you went through that challenged you so much?

R: In taking care of him?

I: Yes

R: The situation I saw was there was Junior was admitted to Mulago, and I had to come to Mulago. I had to pay rent where I was staying and I had to go and do my work so that I get some money, I found a big challenge there, and I was there asking myself what am going to do today, remember where I went to dig, that person needs the garden very fast and this side I had a patient in Mulago. I used sed to come to 10 am, there was time when I had not reached here by 10:00 am, the health workers called me on the phone, and they told me, he had to bathe, hurry up but I was still at home finishing some work to get money so that I board and come.

I: As we conclude, what message can you give to caretakers of adolescents who have this kind of illness, like your son?

R: The message I can give the caretakers is to be patient in everything and not to be rude to patients. It may reach in the morning and the patient has escaped from here, and you look for him, sometimes you get them, and not saying that let me beat him, let me harm him, and also those people who see these patients on the road side not to treat them bad, yes, like beating them, like this is a thief, children go through all those things

I: Any questions you would like to ask concerning what we have been talking about?

R: Nothing

I: Thank you so much, I request we stop here

A QUALITATIVE INTERVIEW ON THE LIVED EXPERIENCE OF CAREGIVER 10 (C10) OF AN ADOLESCENT WITH BIPOLAR DISORDER

Interviewer - I

Respondent - R

I: My name is xx, I am a student at Mbarara University of Science and Technology and I am studying Masters in Mental Health Nursing, I am a health workers who works on mental health conditions, but before we go further, I would like you to introduce yourself, to tell me your names, your age, your religion, your marital status, the period you have spent with the child, the age of the child and your relationship with the child, is the child

yours?, belongs to another person? That's what I want you to first tell me, let's start form there.

R: My name is C10, I am from Seta-Kawule, Kalungu district. I am 40 years old, I am a female, I have a husband, I am married, I am a catholic, I studied up to S.3.

I: For how long have you stayed with this child?

R: The child has been sick; this is the 4th year.

I: What I am saying is that it's you who has stayed with him since his childhood?

R: Yes, since he was young, he is my son.

I: What is his name?

R: He is called Kizito Steven.

I: How old is he now?

R: He is now 17 years old.

I: It means from a young age; it's you who has been taking care of him.

R: It's me who takes care of him; I am his mother.

I: Is he attending school?

R: He completed primary level –P7, he was about to do his final exams, he got the illness, he started getting better and repeated the exams for primary the following year, that's when I started him on medication, I have never taken him back to school, he keeps on relapsing, like that, but concerning school at least he would have gone to vocational institute, but it's still failing, I have not yet taken him there.

I: Is it failing because of illness, or has the situation not enabled you?

R: The illness, support would not have failed, but the illness, he keeps on recovering and relapsing.

I: You as a caretaker of this adolescent with this kind mental illness it manifests in 2 different ways, sometimes it may appear in form of depression, or having elevated mood or being irritable, in English it is called 'Bipolar', hearing from you may help us to understand very well what it means to take care of an adolescent who has mental illness of that kind I have talked about, so on that note, I would like you to tell us what has been your experience ever since you started taking care of that adolescent?

R: The experience is not easy because I take care of him by myself as a woman; his father is there, but cannot provide even a single coin. I am always in that situation of looking for

money to take care of him in extreme pain because there is no money. If I have no work to do, then I have to go to my friends, I dig for them so that I get money to take care of him, and I care so much because I cannot leave him to go and wander everywhere. The ones we see on the streets just wander and move everywhere, but I do not like that. he also has his energy, which he can use to work so that we get money to buy medicine. He is well and in good health, but the situation where we look for money is not good**.**

I: Apart from saying that his father does not provide for him or does not support as it would have been, I would like to know what other things you have experienced while taking care of the son ever since he got the illness, even before he got the illness?

R: The situation has not been good because I have to take care of him, he has to go to school, I need to look for the money, when this came in, it just worsened the situation, when the situation of illness mixes with other needs, I cannot manage**.**

I: On the same issue, can you elaborate more about your daily experiences as you take care of him, when either you are looking for money or picking medicine for him or guiding him on where not to go, the daily experiences you go through every day as a parent taking care of this child knowing that he has this kind of mental illness**.**

R: The situation I go through looking for money is by going to the villages to dig for other people in their gardens, we go, and the person gives us a portion of the field, we dig it, and get paid. We do that for several people to get money, which will bring us back to the hospital, yet other children are also in school; that is the situation we are in**.**

I: On that issue, how many children do you have?

R: I have 7 children, but the first 4 have a different father from the last 3, I stay with the father of the last 3 children**.**

I: Ok, In taking care of a sick person as they told you here, there are certain things they told you like the sick person has to take drugs, you should not put the sick person in situations which can make them feel so much anger, you need to talk to them to avoid peer pressure groups which may take them, so those are some of the situations I want you to explain to me as some of the things you go through as you take care of this child, how do you manage it?

R: He does not have any problem; his normal way of life is not a problem because he does not have peer pressure groups. He is not the person who goes to nightclubs, even for

work, I give him what is enough for him, as you know them. If he decides to rest, I leave him and I continue with my work, and I give him what to eat on time.

I: What about medicine, do you have any problem, like trying to talk to him to take medicine?

R: He takes medicine, I also add on to encourage him to take medicine, but he does not miss taking his medicine; he takes his medicine well.

I: What about at first, when he started taking the medicine?

R: Even at first, he did not refuse to take medicine like others; he takes medicine well.

I: Apart from that, what about getting what to eat?

R: We get what to eat very well, it's always there in plenty because we plant our food, and the food that we need to buy, we buy it.

I: What about other things like clothes?

R: He dresses, because he also works, he can work, he is a strong man, he works and he buys himself a good cloth.

I: Does he also get his work, or it's you who get and you work together?

R: They call him for work, and I let him go to work.

I: He works well?

R: Yes, he works well and he gets his money, he brings it and he buys what he wants, he is also a farmer, he has hens and pigs

I: Before we go any further, I would like you to tell me, how was his condition let's say 2 months before he got the illness, because there is where you said that he has been sick for about 4 years now, before this illness came, how was he, how was his condition?

R: He was doing well, he was studying well, humble, he never had any bad behaviours, and we could get money because we were not spending it like we are spending it now, because of the illness. Everything was moving on well.

I: You said he was doing well?

R: Yes.

I: How did the signs and symptoms start coming?

R: When did he start becoming sick?

I: Yes.

R: He started by being talkative, climbing the roofs of the house, talkative the brain started being disorganised. We used to tie him to the ropes and chase him.

I: Seeing that as a mother, how did you feel?

R: I felt sad, because I had never seen such.

I: What else during that period, climbing the roofs, what did you do?

R: People would get him from the rooftops and put him down, then we started looking for medicine, as you know, how Bagandas are, we later took him to the hospital.

I: So, in search of medication, where did you first go?

R: Traditional medicine.

I: The herbal one, where they squeeze and drink or shrines?

R: No, the one they squeeze to drink.

I: I would like to know about that medicine, for how long did he take it? And when he took it, how did it treat him?

R: As you know, they tell you I know this herbal medicine, they brought it to me, he took it for some time, I think it was for a year, he recovered. I took him back to school and he repeated primary seven class and completed it with final exams, after that, the illness came back, then I brought him here.

I: After going to the other side several times, how did you know that even in modern hospitals, treatment may be available?

R: It's fellow people who tell us. They just told me to bring him to the hospital, there is medicine for those who are mentally ill, that’s when I brought him.

I: When you brought him here, as a caretaker, how was the situation?

R: I brought him here immediately, the health workers worked on him immediately, we were admitted and slept here, and we were discharged the following day, but we went back when the condition he had calmed down.

I: How was the situation when you were here?

R: There was no problem, health workers used to come check on us, and they also bought some medicine, which we wrote for us to buy, which we did. They provided us with the medication that they had in stock, and he took it. They do not ask for money, they just wrote for us drugs to buy for those that were out of stock. I will not lie to you; I just took

my money and went to the pharmacy and bought the medication. If the chance comes when medicine is there, they give it to us.

I: When they gave him that medicine, how was the situation?

R: The situation changed, and he also improved. After some time, towards the end of the year, since he started taking medicine, he again relapsed. When he relapsed, I brought him here again, they worked on him again just as it was during our first time, they did not blame me, we spent here like four days, and they discharged us, we continued coming back for medicine.

I: When you were discharged, you went back home. How was the situation? Didn't he disturb you, not wanting to take medicine?

R: No, but as you know, he was there, but not running, just entering the house, sleeping, like that, the symptoms continued reducing like that until when he fully stabilised, but he has never refused to take medicine, I used to give it to him and he would take it.

I: What I want to know is, when health workers worked on him, did they explain to you the kind of illness your son has?

R: No, because they say they do not measure the illness. When I explain to them, like the way we are seated here, I think they just understood what my son was suffering from because they just asked about how the situation had been.

I: What I am asking is that, after talking to you, did they tell you that, according to what you have told us, we have seen that the child is suffering from this and that?

R: No, they did not tell me

I: What did they tell you they are treating? When they said that the son should start taking medicine.

R: They are treating mental illness.

I: Is that what they told you?

R: Yes.

I: Ok, but they did not continue explaining to you that this kind of treatment is like this and that?

R: Since I was not the one taking care of him when admitted, I brought him, I explained to them, then left him with another person and went, I would just come back, I do not know

whether they explained it to the one who was taking care of him, because me I do not know.

I: Ok, he did not tell you?

R: Yes, he didn't.

I: I would like to know your relationship with him. How is it?

R: The relationship is not bad, because if it were bad, then I couldn't tell him to do something, and he accepts, but he accepts whatever I tell him to do. Whatever I tell him to go and bring, he brings it well, he does not have any problem concerning that.

I: What about before?

R: Before, he used to be argumentative. I would tell him Do not go there, he says I am going, when he was still sick, but it's no longer there.

I: You see, these drugs helped him in this?

R: Yes, sir.

I: So, the relationship at first was not good?

R: Yes, because he was sick, he was mentally disturbed, but ever since he started taking medicine, he stabilized.

I: What about your relationship with your immediate family members? Like his siblings, your husband, though you said that sometimes he does not care, but how was their relationship with him?

R: The relationship was not bad because they all used to help me, and they used to come wherever they were to come and visit me, some we used to come with them to the hospital, they are the ones who used to catch him.

I: It means they helped you so much.

R: They helped me so much.

I: What about the neighbours, the ones you stay within the community?

R: They all helped me, they used to call me whenever he relapsed, they normally catch him, I would go and take him back home, they did not treat him in any bad way.

I: Some people say this is so-and-so's mad person.

R: No, they were there giving me information regarding him; others used to get on boda bodas going after him, catching him, and bringing him back home.

I: Did you have any bad relationships with your family members or people around you due to your son's mental condition?

R: No, I did not get it**.**

I: Ok, so now, I would like you to tell me about your relationship with mental health workers who treat your son. How was it the first time you met them?

R: They do not have any problem; they took good care of us**.**

I: What shows that they care about you?

R: When I go to someone's office and I take the book to check up, they counsel me without doing any bad thing to us or throwing our books at us, we have not experienced such here**.**

I: Were you stigmatized or discriminated against because you have an adolescent with this kind of mental illness-bipolar?

R: No

I: No, it has never happened?

R: Yes, it has never happened**.**

I: In your judgement, which kind of counselling do you think a caregiver of an adolescent with mental illness should receive so that they can stay well with their adolescent?

R: Giving them good care, because sometimes you have to give them what they want if you have it. For example, when we prepare our food like posho or cassava and he does not want it, then we prepare matooke for him. If he wants to drink soda, I have to buy it for him, and if he wants to eat rice, I have to buy it for him. Also, not giving him a lot of tasks to do, you have to weigh and give him reasonable work that he can do**.**

I: Why do you fear giving him a lot of tasks to do?

R: Sometimes his brain may become excited, if he keeps on moving from one place to another, they can relapse sometimes, and you see them changing from their normal way of living**.**

I: You as a mother with a mentally ill adolescent, are there times when you get worried about him, let's say, when you leave him at home with other siblings?

R: I used to get worried, but it's no longer there because even if I leave him now, he no longer has any problem**.**

I: Why were you worried?

R: Because I may leave him, and I find that he has harmed them since it just comes at any time, but now I no longer get worried.

I: In that situation of being worried, what did you use to do?

R: Sometimes I used to stay home, not going anywhere, I stopped going, just staying home, or if I go somewhere, I come back quickly, I make sure I do not spend there a lot of time, and I also tell them when you observe him doing this and that, hide yourselves or leave him.

I: So that was a challenge in that sometimes you were hindered from leaving home because you feared that he might relapse.

R: Yes.

I: What about the issue of having to provide him with necessities? What challenge did you or do you find in that?

R: I have to provide for him, I have nothing to do, I have to try in every situation to see that I provide for him.

I: We talked about his academics; I would like you to explain very well the reason why he did not continue with school.

R: It was because of mental illness, he would have performed well in his final primary exams, but he got the mental illness in the middle as he was going to do his mock exams, that is when he got the mental illness, when he went back to seat for his primary leaving exams, he passed 2 of the exams and failed the other 2, we then prepared to take him in a vocational institute but he then relapsed. I first paused at that; he never went back to school.

I: What is the plan now?

R: The plan is that I want to take him to a vocational institute so that he learns.

I: Which do you want him to learn?

R: He wanted to learn vehicle repair.

I: Ok, you said that before you came here, that's where you went to get treatment. I would like you to elaborate more, where did you go, what did you find there, and your experience of what you saw?

R: We did not go to places, it was like when you are there and someone tells you that I have such and such medicine, for example someone gave us moulded clay mixed with some

herbs ‘emumbwa’ we mixed it with water and then gave him to drink and he calmed down, but there is no where we took him, no.

I: Ok, when you gave him that medicine, how was the situation?

R: He used to calm down a bit**.**

I: There are those people whose children get such conditions, they think that they have been bewitched, or there is something not right, family spirits are not fixed. I would like to know when he got the mental illness, what did you think at that time, as a parent?

R: No, I did not think such things, it did not cross my mind, no

I: Some people, when they get such problems, go to church

R: Like going to Pastors?

I: Pastors, churches, or repenting to the church Fathers**.**

R: No, I did not go anywhere, and I did not pray

I: You may even pray from home**.**

R: Yes, there are prayers we read, we read those home prayers, but again, there was no difference**.**

I: What advice can you give parents or other caretakers of adolescents with mental illness, especially the condition your son has?

R: The advice I give them is to take their children to modern medicine, because there is no traditional medicine that heals mental illness. Most of the people I have seen, even those we talk to report going to the hospital or going to Butabika hospital, or any other government hospital which gives medicines for mental illness, that is where everyone must go**.**

I: What about how they are treated at home? How would you advise them?

R: Counselling them, because you may quarrel with a person and the brain gets disorganised, they need to be handled well, and not being mad at them**.**

I: Sometimes when people take these drugs for mental illness, they get side effects as a result of swallowing the drugs**.**

R: What are the side effects?

I: Drugs are not treating them well. Sometimes they salivate more in the mouth, the tongue comes out of the mouth, and sometimes they become too weak. When he was given medicine, what did you see as a parent that somehow made you get worried?

R: He becomes weak, there are days he becomes weak, on that day when he is weak, even when we go to work, he becomes weak and sleeps in the garden, and there are days when he has energy, that is the only thing I see in him, sometimes I think it's the drugs which are stronger.

I: On the drugs, did the health workers explain to you about some of those things and what you can do in case he gets such side effects related to the drugs?

R: Today I told the health worker, he has been taking 2 tablets in the morning and evening 2, he has told me I give him 1 tablet in the morning and 1 tablet in the evening, because he has told me that he has been swallowing 60 tablets per drug type in a month, now he is going to be using 30 per drug type.

I: He has been using 120 tablets in total.

R: Yes, now he has told me to use 30-30 each drug type.

I: That is 60 in total.

R: Yes, that is what he has told me.

I: You told me health workers treated you well, that is so good, but I would like to know from you as a parent with an adolescent with this kind of mental illness, do you feel that you know, you have the ability and understand very well as regards to the kind of mental illness your son has? are you sure of them?

R: Yes, I am sure.

I: On a scale of 10, how can you rate yourself? 10 means you are very sure.

R: I give myself 8, I will not give myself 10.

I: And if you see another person with a child in the same condition.

R: I advise them like what I do, I must tell them to bring the child here to the hospital, they will tell him what to do next.

I: Sometimes this kind of mental illness comes when we do know that it's a mental illness, at first, what did you think when this illness came?

R: I thought he had meningitis.

I: You thought he had meningitis?

R: Yes.

I: Later, what did you see?

R: It continued, his energy continued increasing to a point that he could not be touched, because we brought him here when he was on ropes, but when he got that injection, he started calming down.

I: When you saw him on ropes as a parent, how did you feel?

R: I felt bad, I even used to cry, I could not sleep, I could not eat food, yet I was the caretaker, it was like both of us were sick, I could not do any work, no.

I: So, in that situation, what can health workers do to see that the caretaker should not also be worried too much?

R: They do counselling, even in the community, people counselled me, telling me to be strong. And when I reached here, I found many other people in the same condition. I became strong seeing others, because for me, at first, bringing him, I thought I was the only person. When I reached here, they brought another one, and another one, I said it's not on only me, I became strong seeing other people.

I: After he got into such condition,, not walking well, there are some things which have not moved on well and what I know is that you also have to do some adjustments, sometime in how you having been working, I would like you to tell me about that like may be before my son became mentally sick, I used to do like this and but after he became sick, now I changed I do like this.

R: Things like what?

I: Usual things, maybe before I wasn't caring so much to see whether he ate food or not but now I have to make sure he eats.

R: Of course, I have to provide him with the necessary care, at the time of admission and after his discharge from the hospital, I would first cook for him breakfast, tea, and porridge before leaving for work, but as he recovered, I stopped doing some things. When we come back from the garden, we first give him what to eat, the situation that was not there before he became sick.

I: Other people in the community, when they find him playing with their children, or interacting with them, what happens?

R: Nothing happens because after he recovers, he goes back to his friends, then people give him work to do as they pay him some money, they do not avoid him, no, they do not segregate him.

I: He has reached the age of bringing in-laws.

R: You never know, I do not walk with him everywhere.

I: Haven't you gotten any challenges with it? Maybe they report to you that your son is doing this and that?

R: No.

I: Is there a time when he committed a crime due to the kind of mental illness he has?

R: Maybe, he used to fight, he used to beat people because of mental illness, he used to beat them, only that.

I: Tell me more about a scenario you remember when they reported to you.

R: They would not report it to me. When he would fight them, they would get defeated and run away from him, and I would find out about it later, after the fight. No one came telling me that he had beaten so and so, no, but when he started recovering, they started telling me he beat so and so, they also beat him, but he was unbeatable.

I: You said when you saw the illness, you did not think that it was due to the Family?

R: I did not.

I: But in the family, are there people with such a kind of mental illness?

R: I have ever heard about it that in their family they used to have such a kind of illness, the person who told me used to take them to Butabika Hospital, and they recovered, and the person told me that one of them is outside the country.

I: How has the situation been ever since your son stabilized on medicine? It has now 4 years since he started taking medicine, how do you see the situation, how has it been?

R: The situation is good.

I: Ok, is there any other challenge?

R: No.

I: He is an 18-year-old adolescent. Why don't you let him sometimes come alone to get his medicine?

R: I would have left him to come alone, but sometimes I am the one supposed to go to the pharmacy to buy him medicine, but he will start coming alone to get the medicine. Sometime back, he used to come alone.

I: Because I was going to ask you, are there times when you get worried that if you are not there, how will he live?

R: No, because I tell him and emphasize it that even if I am not there, you have to take the medicine, he has ever come by himself, he knows where the hospital is, even if he has not come, the same medicine is given in Kalungu, he can request for a letter and he starts receiving it from Kalungu**.**

I: Let me ask again, as a caregiver, what challenge have you experienced while taking care of him?

R: The challenge is money; money is scarce. I reached here, they tell me medicine is to be bought, yet we have spent a lot on transport to reach here, and when we requested to give us drugs for at least 2 months, they said they do not give 2 months. We have to report here every month, it burdens us because we may come for drugs and it's not there yet, the transport, we have used would have bought the medicine because that is where we can buy it from, that is what challenges us**.**

I: Apart from that, anything else?

R: It's only that, because I use 20,000 shs coming and going back, yet that 20,000 shs I can use it buy the drugs, because sometimes I come and there is no medicine. Sometimes I use 50,000 shs, yet if I do not come and I skip that appointment, that 20,000 shs can help me to buy the medicine, but they don't like it that way. Every month, I have to report to the hospital, we come but do not get the medicine that is the most challenging thing, it's not only me, even other fellow caretakers**.**

I: When he takes the medicine, doesn't it treat him in a bad way?

R: Only making him weak.

I: When staying with him, do you give him tasks to do like other siblings?

R: Yes, I give him tasks to do because his siblings go to school and we stay at home together with him, he does some work, it's not a lot of work, it's just going to the garden to dig, we come back, bringing water from the well, like that, but it's not so much work**.**

I: Hasn't he ever asked you when he will stop taking the medicine?

R: He has ever talked about it, we came here and they told him, he will not stop taking it, if he stops taking it, he will relapse**.**

I: What about his relationship with other children? How is it?

R: The relationship is good.

I: Sometimes when you look at your son, and look at other children he was born with, some are at school, 18 years some are about to complete their S.6, how do you feel about that?

R: I do not feel good because I was going to pay his school fees so that he could also reach a certain level, but the situation could not make it possible; he also wanted to study.

I: So, there are moments when you feel worried**.**

R: I also get worried.

I: Are there moments when you judge yourself, maybe you blame yourself as the root cause of the problem?

R: No, I do not judge myself because I am not the problem, because it's not only my son, no**.**

I: Anything you would like to ask? or what would you like to tell me concerning your son?

R: No, I do not have**.**

I: Ok, thank you so much for explaining the situation in which Steven is in now, and thank you for taking care of him, what has made me glad is that you do not have any situation of trauma, you know some are segregated where they stay, but for you have said it's not there**.**

R: It's not there**.**

I: That is important, good news, and we appreciate and pray that you continue taking care of him well. Thank you so much. I request that our interview stop here**.**

A QUALITATIVE INTERVIEW ON THE LIVED EXPERIENCE OF CAREGIVER 11 (C11) OF AN ADOLESCENT WITH BIPOLAR DISORDER AT MRRH

Interviewer - I

Respondent – R

I: As I have explained to you, my name is xx. I am a Mental Health Worker, but right now I am also a student at Mbarara University of Science and Technology, and I am doing a Master's in Nursing, but focusing on Mental Health conditions. I am doing research and focusing on understanding the experiences of caregivers of children who have bipolar disorder. Before we go any further, I request you to tell me your name, your age, your religion, and your marital status

R: My name is C11, I am 52 years old, I am legally married to my husband is KJ

I: What is your religion?

R: I am a Catholic

I: Ok, about school?

R: I did not go to school; I stopped in Primary Seven

I: You stopped in Primary Seven, did you study and complete it?

R: Yes, I went a bit senior one, but I did not complete it, I stopped in Primary Seven

I: Ok, what is your relationship with Musoke?

R: MM calls me Aunt

I: He calls you Aunt

R: Yes

I: For how long have you stayed with him?

R: He was given to me when he was 3 weeks old, but when he grew a bit older, my child took him to stay with him

I: But he was given to you when he was 3 weeks old

R: Yes, his mother was also a Mental patient, she gave birth to him and spent 4 days then she became Mentally sick, then they took him away from her, my mother stayed with him but she was old, she could not take care of him, then when I told my husband about it, he accepted and we took him from my mother and brought him when he was 3 weeks old, so we have stayed with him, all the time

I: What is the relationship between you and his mother?

R: Wife to my brother, I am his Auntie

I: Is his mother still there?

R: She passed on, she was mentally sick when she was knocked by a car, and she died

I: Sorry, so from 3 weeks old, you have been staying with him?

R: Yes

I: You have seen all, you have seen how the illness came in

R: Yes, he has grown up doing well, but he got mentally sick recently when he got a fever

I: Ok, so now, how many children do you have?

R: Four

I: Four

I: Ok, is there anyone with a Mental Illness among your children?

R: No

I: How old is he?

R: He is nineteen years old

I: You as a caregiver of that adolescent with Mental illness which manifest in 2 forms; sometimes it manifests in so much depression, or having elevated mood or being irritable, it is called bipolar disorder, hearing from you may help us to understand very well on what it means to take care of an adolescent with this kind of Mental illness. On that note, we would like you to tell us your experience as a caregiver

R: At first, I did not find any difficulties with him because he was doing well from his childhood, he was eating well, drinking well, and he grew up well. He wanted to eat so much, and we first thought that because he did not breastfeed, he grew up wanting to eat so much, even if it is to drink and you give him a bottle, from childhood, he could drink quickly and finish it while crying, but not get satisfied. We then gave him some medicine for tongue tie because we thought it was a tongue tie affecting the Frenulum; he used to love eating so much.

He is a quiet person, he does not speak a lot of words, he went to school, but he was not performing well in academics, his brain was not capturing well. After Primary Seven, we left because it did not even go well with the Primary Seven final exams. We took him to the garage and he started motor vehicle repair. He was not having any problem, the main thing was being quiet, but he would eat so much, not reducing, all the time; he wanted to eat.

Even her mother used to love eating so much, sometimes picking raw mangos from the trees, sometimes eating what had not been given to her, sometimes even eating what she had prepared for you, you find that she has already eaten it, so even that child had that same thing. We thought maybe because of her mother, but we left it there, you give him what to eat.

As he grew up, he left the garage and he started washing cars, he does it as a source of income, he washes cars and he can buy himself what to eat. He is a quiet person, he does what you tell him to do, but if he does not want to, he does not do it, if you tell him to him and he will not like it, he stands up and leaves you there.

His main illness is nose bleeding; he gets nose bleeding so much, whenever he gets flu or what, he gets nose bleeding. I have never seen him like he has something which has annoyed him so much, and he quarrels, and he is a friend to young children. He does not interact so much with adults, but likes to be with young children.

Recently, in March this year, he got the flu and nosebleeds, but he is now 19 years old. I have not had any problem with him; we are used to his behaviour, quiet, speaks few words, interacts more with children. He loves children, we could get worried, ask ourselves about him loving children so much, you may find that you leave a child with him in the house, and he causes harm to them, he loves those young children. Since we knew her mother, it could make us get worried about that, but he does not have any problem. If he does not want to do what you have told him, he gets up and leaves you, but does not respond to you.

He has been getting sick and has recovered, but recently, he became sick; he had the flu, he was coughing, and he had nosebleeds. He became sick and was not recovering, with nosebleeds all the time. He went and he got some treatment in the Clinic, but he did not recover, and they told him it was a fever, but he was not recovering, he was there saying he did not feel fever, but he is weak, but I could just ask him but he couldn’t tell me. Whatever you are going to give him, you must ask him whether he is in pain, or not in pain, but he cannot eat the way he used to eat; he cannot eat now, yet his life is eating. He is a nice-looking, handsome youth. You did not see him here when we came, but he was no longer eating, he was weak, and we could just force him to leave the bed. When they forced him out of the bed and he sat on the veranda, where he could call any child passing by to come and they be there.

When he became mentally sick, he just woke up in the morning asking his mother to tell him what had happened to him; he asked his mother to tell him if he had died and was not being told, he further said that he was no longer in the state that he used to be in. I asked him, ‘You are saying you died – don’t you see that you are alive, if you died, would I be talking to you?’ He said you may not be telling me that I died, I am no longer in my usual state, like the way I used to be, even when I am walking on the road, and I find people and greet them, they do not respond to me. I asked him, ‘You are saying you are sick and you have been here, when do you walk?’ He said that when I leave here and walk a bit,

even when I greet people, they do not respond to me, and also, even when I call my friends on the phone, they do not pick up my calls, and they no longer call me. It means I died, I told him, no, you have never died, you are alive, but because you are sick, that is why they do not want to bother you. He started crying and crying.

One time I told him, MM, does not cry, you are alive, I am your auntie, don't you know that I am your auntie and your mother? He said yes, do not cry, when I talk don't you listen, he said I listen, don't you see that I am talking to you, he said I listen, I told him, I love you, whether your friends hated you, leave them, and he asked for what to eat, and I gave him and he ate, and he was there.

Then I told someone who is a health worker, I said that health worker about the situation that the child has told me, and the health worker said 'Let me come home and see him,' he was working from here but he retired now, he said that he will come and talk to him. He came and he asked him some questions but at first, he was not responding, he asked him and he was responding in a few words, and he told him maybe because you are a health worker but people hate me, it's only my mother who loves me, maybe it's because she is my family but all the rest do not love me, but because for you, you are a health worker

I: He calls you, his mother?

R: Yes, he has grown up calling me mother; he does not call me auntie, but I am his aunt.

He said it's only my mother who loves me, but because you are a health worker and he asked him they give you food, he asked him some questions, and he said it's my mother who will give it to me because others will not give it to me. The health worker said that maybe this child has brain fever, so you will take him to the hospital. I did not take him immediately; we first stayed home, and he took some medicine.

One day, his father who is my husband, he also calls him father told him you know what MM, you are going to go to the hospital and he said you are taking me to the hospital yet I am taking drugs, I am not going to go there, I am not going to go there. I told him accept your father is going to drive you in a car and take you to the hospital, he said I am not going, he refused, then his siblings (my children) came and they told him that today MM you are going to bathe and we go to the hospital, they normally call him 'Uncle'. Uncle, you are going to bathe, and we will take you to the hospital. He fears them they

are older, he said yes, I am going, and they told him that mother is going to take you, have you accepted, and he said yes, and they went. I told him, ‘Have you accepted that we go?’ He said yes, then I told him now bathe and we would go to the hospital, and he said, I will go to bathe, you are also going to bathe, right? I said yes, I had brought him from the bedroom and I had put him in the sitting room, according to his behaviour, I was not going to his bedroom. They sat at the dining table when he was there. He said ‘Let me go to my bedroom and bathe. 'He went to the shower.’ I also went to shower, later when I left to go to the shower, I did not see MM, he had gone

I: He ran

R: Yes, I was not seeing him, I looked for MM, I called him, I looked for him in the garden, I asked the neighbours, they said he has been here seated. Usually, he does not go to people’s homes, and as I have told you that he is quiet, he does not greet people. He just greets them when he is going to work. This time he went into a person’s house, and he entered and sat there. that lady’s husband was not there; she was there alone. The lady called a youth, and he told him to chase MM away. Then they chased him away and he continued and I told them you would have called me, don’t you know that he is my child, they said we saw him coming back, I told them don’t you know that he has been sick, but they did not know what was going on, they said he has gone that side.

So, I started looking for him, I was going to go to the community radio, we have a community loudspeaker. As I was there, I saw the youth who works on that community radio coming, bringing him to me. He was catching him like a thief, and he told me this youth had been entering people’s homes but when I looked at him, I saw that he is your son, if he was not your son, I was going to beat him, they steal during the daytime pretending to be mentally sick. I told him you would have first asked me because you have never seen him or heard him somewhere stealing, then he brought him to me, and I called my husband on the phone, and we brought him here.

On bringing him here, we first went to the other general side, but when we reached here, we found student nurses, and he called them and told them, Come and be the ones working on me, he wanted to touch them, kissing them, and he became worse

I: That’s what he wanted to do?

R: That's what he only wanted to do, and it's the health workers who came to him quickly, wrote for us, and brought us here to catch him. We came here on Wednesday when they are on a ward round, they told us that they do not work on that day, it's my husband who looked for a health worker, and they worked on us, they worked on us from this room for 50,000 shillings. We had to catch him because he was going to rape women and health workers, but health workers knew his behaviour. They injected him with medicine, we had to be there because if he got the energy again, he could run around looking for women, telling them to come, pulling their hands, we were in that situation. He was also stealing people's food. When he woke up, he was picking people's bread, he could pick any food he had seen. Even when we gave him food, he ate a little and then went on to steal other people's food.

We spent 3 weeks here, but during the period we spent here, they used to inject him with medicine, and he calmed down, he later stabilized, and he was taking tablets well. My children used to take him to bathe, I was with 2 boys who were with him, and his young brother was there, and his father was there - we used to be many to protect him. He used to go to bathe and wash his clothes when they were there protecting him, but when you could say that you leave him alone, he could not come back, run to see women, he could escape from us, we were in that situation.

He used to get something that used to catch him on the neck, and he could shout so much that thing used to make him shout. The health workers changed the type of drugs he was taking, and they said he would stabilize on treatment

I: As you were still on admission?

R: Yes, we were on admission, they changed the drugs, and we were there for the next 2 days, and it did not return. Later, we saw it coming back, yet they had changed the medicine. He started stabilising slowly by slowly and he stabilised, we used to come here and they used to ask him and he was answering to the questions, they used to ask him questions, he was not so much out of his senses, he was understanding most of the time because I did not see health workers asking him and he fails to respond. He used to answer them, but sometimes he could leave the health worker before completing what he was asking; he saw as if the health worker was wasting his time, and he left.

Even on the day they discharged us, that thing came back and pulled him so much, and he shouted so much and called me to catch his neck, we could just catch the neck as if it were falling. On that day, I thought they were not going to discharge us, because it pulled him after they had discharged us, the health worker gave us the drugs and told us that he would stabilize, yes

I: Ok, before the illness came in, how was your relationship with him?

R: He used to be my friend, from way back, he is my child, he loves me so much

I: What about here when he got the illness?

R: Again, he has been loving me, he has not been having a problem in that, and when I threaten him, he fears me more than others

I: Ok, so you have gone back home. Has he been taking the medicine?

R: He has been taking it well because he has been in his right state of mind, but he has been weak, sleeping most of the time. When I delay giving him medicine, he calls me 'mother I have not yet swallowed the medicine'.

One time he asked me; How long will it take when I am taking this medicine? Whenever I take medicine, I do not get energy, so when he went back to work where he washes cars, he spent there small period and he came back and he told me it seems they have something bad they did to me

I: The one who gave him medicine?

R: No, where he works from, and he told me that he no longer wants to work from there. He said 'Let me stay home, I will get another job, I did not argue with him because health workers said we should not shout at him. Then my daughters, who are born again, asked to take him so that they can stay with him, help him to get a job, and also pray for him, but I told them that they should also continue giving him medicine

I: Let them pray as they also give him medicine

R: Because health workers emphasized that he should not stop taking medicine and they told us that whenever we go to the hospital, we should go with the book we had here, that is what he gives there so that they can get for him medicine, so they took him. By that time, he could do house chores like washing utensils, mopping the house, and cooking.

I: Ok, now, I would like you to tell me your daily experience in your work, as you are taking care of him

R: As I am taking care of him?

I: Yes

R: I have been staying home alone

I: I mean, when you were taking care of him before he went

R: When he was sick?

I: Yes, even after discharging you

R: When they discharged us, I did not have any difficulties because he used to sleep, I used to wake him up to eat, and sometimes he woke himself up, I used to wake him up to take medicine, I did not find any challenge

I: What about before?

R: Before he disturbed me so much because he had even refused to come to the hospital, he disturbed me so much, but it did not take me so long before I came to the hospital. He never agreed to come to the hospital, and he was even tired of the medicine he was taking because he was not recovering, and he was saying I even died, though you do not believe that I died, but you are giving me medicine. Why am I dead but taking medicine? He used to refuse, he used to refuse, but it did not take a long period, I brought him to the hospital. I had never seen the illness that he had, and he had never had mental illness before, and I had never seen it. Since he was young, I have been with him.

He went to his father while he was still young, at his grandmother's home, that is where his father is, but again, he did not have any problem. His father had called him and he was helping him with work, he was there when he was around Primary Six, Primary Five, and then I brought him back because there was a wife to his father. He had told them that he would beat her if she did not give him enough food. He told them I will beat her, that in case that mother does not give me enough food, the other side, mother gives me food which I eat and get satisfied. Here she gives me little food, yet she gets it from the garden, the other side. Mother gives me enough food, yet she buys it. He said I will beat her if she gives me little food. They asked him if he could beat his mother. He said she is not my mother; he told those things to the other people, but not me. When I saw him becoming like that to them, I said, 'This child has grown up, what if he beats this woman? So, I said, 'Let me take him back, but I had given him to them in pain'

I: Ok, some get a challenge sometimes in finances as they take care of adolescents with the kind of illness like which MM has, what do you say about that?

R: True, I do not have money, that’s why I had some difficulties, because if I had money, I would have taken him to other hospitals like Byansi. We usually fear this place because of long waiting lines, but it is where you are supposed to come because you do not have money. As I have told you, he wants to eat so much, sometimes I may fail to get him what to eat because I do not work, I stay home, and I have to wait for my children until they send me some money.

I: Some get a challenge with time, they find themselves spending all the time with the children, the time they would have used for other things

R: You can spend it on a child because, like him, you fear that he may run away from you and go, yet he is sick, he is at home, you have to sit there and take care of him. Getting ulcers, sometimes you find yourself not preparing anything for yourself to eat, you have to look for it, you don't have time to look for it because you will not leave him alone at home, you understand?

I: What about a challenge may be in buying medicine, you have come, it is not in stock, they have told you to buy it

NF: Yes, there is a time when we were here, we got a problem because medicine used to be bought, it was for buying, we used to buy it but with a lot difficulties, sometimes the health workers would ask for the medicine when you haven’t bought it because of lack of money

I: Ok, so where is he now? Where do they get medicine from?

NF: I have not asked them where they get it from; the most important thing is that he takes it

I: They are in Entebbe

R: Yes, they are in Entebbe, but not the side of the airport. I do not know the area they are staying in very well; I do not go there, and I do not know that side of Entebbe. I have never gone to Entebbe, but I went directly to the airport. I was picking some people with my husband, so I do not know the city very well

I: Ok, they can go to Entebbe grade B. There are mental health workers, they can get him the medicine from there.

Now, I would like to know the relationship between you and your close relatives or those near you, how has it been ever since you got this child, and later when he got the Mental illness?

R: Relatives, my siblings, or neighbours?

I: I am saying his, the side of his mother?

R: We do not have the side of his mother reason being when her mother got married in our family, she gave birth to the child and she got Mental illness, they treated her and they failed and they took her back to her home that side of Mutukula, as you are going to Tanzania. We went back there recently for the burial when his mother had died. We do not hear so much from them, but on my side, my siblings, we work together. Our parents died, we are 3 siblings who are still alive, we do not have money, but we relate well.

I: Don’t they discriminate against you because you have a child with Mental illness?

R: Will they discriminate against me? He is our child, all of us. I help them to be with him, and also, I can tell them I have failed. You can also take him, they do not discriminate against me, we all work together

I: What about your relationship with people in the community? How is it?

R: I do not have any problem with them, I am a people’s person. Even if you want to be angry at me when I am not angry at you, you are just disturbing yourself

I: I am saying sometimes not easy for you to relate to them because of the child you have, like what you talked about

R: No, that one was a tenant, you understand, and where she was renting, those people were not our friends, as you know, neighbours, chicken destroying some things, everything, you quarrel like that. They were not our friends and their parents died, it’s the children who are there, they stay in the cities, so when they come, they are not our friends generally, but for me I greet them because I am a mature person and they are young, so if you do not like to respond to me, it’s up to you. I have many friends, and they were able to sustain me as I was in the hospital. Someone comes giving you 2,000 shs, 3,000 shs like that, but for them, where the child had gone, they are not our friends

I: Ok, have you ever experienced a situation, especially one that affected you mentally from family members, and it was associated with having a child with mental illness?

R: No, it's other thoughts, not about the sick child

I: Let us go back to when you came to the hospital. How did the health workers treat you?

R: They cared, the health workers were caring, they care when you bring the medicine they have prescribed for you to buy, I cannot say that they treated us badly. The day we came we got some disturbances, not the other general side where we first went, but when we came here. The other general side we had no problem, they worked on us quickly because they did not want to be with that patient, but when we reached here, we got disturbances because we came on that day they do not work, they were reviewing those already on ward, so we got a lot disturbance and if it was not for my husband to be around, we do not know. They were seeing him as mentally sick, touching those who were passing by, or doing what, but they were not caring to work on us, yes, but we are used to those being the behaviours of health workers, we know them

I: So, you have brought him here, you have explained to them everything in this room, you have explained to them what the health worker told you, what has happened to the child?

R: That is what I am asking myself up to this time; they have never told me what he is suffering from up to now. They asked me as you have asked me now, and I explained how the child started becoming sick, and they wrote down, and they told me we are going to admit you, even though the child was being asked, because I was with him. They admitted us, they started giving us medicine, they did not tell us, and I am still asking myself that up to now, they have never told us that maybe your son has brain fever, or what. They asked me if I see him with peer group using marijuana or what, they asked me everything, my son is calm as I have told you, they even told me at washing bay where he used to work that if he does not have what to do he just sits there on his phone, I did not know about it whether he uses marijuana and I explained it to the health worker that I have never seen it.

He has ever driven a customer's car and he made an accident, and it was destroyed but because the customer was his friend, he forgave me, when he came to my home, he found me also talking well, and he said, you speak like your son, this car has been destroyed, I was supposed to make you buy a new car, but give me little money. I even sold part of my father's land to pay that money, because I did not have the money. When he destroyed the car, he came back home, kept quiet, entered the bedroom, and slept. He did not tell me what had happened until they came. I just called him to ask him what brought

him so early, and he just went to sleep, what? he told me I have problems, but he did not tell me the problem, until they came and told me

I: So, if I may simply ask you, what illness is your son suffering from?

R: I do not know it, he became sick as I have told you that my son became sick, he had a cough and too much flu, and he had nose bleeding, as I have explained to you. So now I do not know what my son is suffering from. If you have not told me, how can I know what my child is suffering from? Up to now, health workers here have never told me, I do not know whether my child is having brain fever, so when I take him for prayers, I do not see any problem in that because they never told me what my child is suffering from

I: Let me ask another question, which medicine does he swallow?

R: The one they write, I take the book and they write in it, tablets, there is a small tablet, they are different, but I do not know how the drugs are called, and I never went to school like some people, I do not know

I: Didn’t they tell you?

R: No, they say the name of the drug that go and buy this type of tablets but at that time, I say that health worker, will I reach when still recalling that name, and the health worker says bring I write it for you, you see that, so when I take it to the Clinic, they check on what it written and they give it to me

I: Ok, we are still on health workers, they told him they are going to start him on medication, did they explain to you what the drugs do?

R: At first, they told us that we had admitted you, go to that room, and we will show you where you are admitted. They told us to go and get an empty bed. We got a bed, then the health worker said another health worker is going to come and inject him with medicine because we told the health worker that the patient was unbearable to be with, wanting to walk and go away all the time, and the health worker said they are going to come and inject him with medicine. While we were there, the health worker came and injected him with medicine, then he calmed down, that day we did not get some much treatment, apart from that injection he got, then they told us the file had not gotten the number so we shall go back the following day because they were tired, so that we begin to get medicine. They injected him with that medicine and said that he would sleep.

When we came back, they looked for his file and got it, they gave him medicine that the other fellow patients were getting. There was nothing like maybe this patient gets this kind of medicine and this one gets this type, they came at once and they gave them similar tablets. I do not know the kind of illness they are treating, that is what I am asking myself, my child was young but I was seeing those severely mentally sick, some they said had come for like 3 times, they treat them, they go back home, others had stopped taking medicine but they took it again. They were all getting the same medicine, I did not understand that one also, you know, when you behave like you are too wise towards the health worker, they may not treat your child, yet you need treatment. My son was young, he had never got medicine for mental patients

I: I was also going to ask you that question, when you reached here and saw those who were severely sick, how did you feel in your heart?

R: I felt so bad, what made me feel bad they were giving us similar drugs, you understand that, yet there are mental patients who would have been in their special room, and we are in our, maybe when we are in one place but when it comes to maybe they have checked your file and they have written for you that go and get the medicine. I used to get medicine for him medicine but they used to get medicine from the same tin, what they had written for me, even the other one, even the other one, even the other one, we used to go and buy it in Mulago, it was similar tablets, yes

I: Patients are there, but sometimes there are medicines they take which are similar; you find that they are treating the same kind of illness and get the same tablets from the same tin

R: May be the patient they separated, the patient came they said he had injected marijuana in his body, he used to sleep in his room, I do not know the medicine they gave him, but I do not know whether others knew the kind of illness they were treating, but for me I was treating mental illness because he had become mentally sick

I: It’s what I have asked that when you saw those who were severely sick, didn’t you say that my child may continue becoming severely sick?

R: Yes, I thought about it because some came when they were very mentally sick, I also brought mine, mentally sick, because he was touching women. When health workers

come, he touches them, calling health workers, saying goodbye to them, laughing, which he was not doing

I: Ok, have you ever experienced a situation of discrimination because you have a child with a mental illness?

R: No, because he did not take long when he was sick, people still loved me

I: Which kind of help do you think should be given to caregivers of adolescents with a kind of illness like which MM has?

R: Help from usual people or health workers?

I: From people, whether health workers or family, all of them

R: I think the most important help is to come and treat the child as one because it’s not easy to treat that patient when you are alone

I: As a family?

R: Yes, as a family, it’s not good to leave that patient to you because you may go somewhere and you find when the patient has done something else, what if he rapes someone’s child, it’s good for the family members to come together and treat the patient, but my siblings did not leave me alone because even his father also came and we were there with him

I: His father is there

R: Yes, his father is my young brother; he does not have money, but he came and we were there with him and we treated the child together. I see that it is a major help, and also money because medicine needs to be bought, food cannot fail to be gotten because we have friends, a person gives you, we cannot fail to get food

I: What about on the side of the health workers?

R: Health workers treated us

I: About counselling, do you think counselling a caregiver is also important?

R: Yes, and not to burst out verbally at me, I do not want that because even to my child, I do not burst out verbally to him and also a mental patient is a mental patient, it’s not easy to handle him. Sometimes he escapes from you, goes that side and you go there to get him. There would have been counselling because you also did not want it to happen to your child.

Another thing, let them tell us the kind of illness our children are suffering from, that your child got a mental illness because of such and such reason. You see that I want it so much, even up to now, I do not know what causes mental illness in my child. When I came here, I got one health worker who became my friend, I asked the health worker what caused the illness and why he became sick. And the health worker told me that they did a check-up on him and he did not have a fever. I told the health worker that because the fever was treated, and the health worker said, 'Bring the tablets you have been giving him'. I went back home. I brought those tablets because he even had the flu, and the health worker picked one tablet and told me to take these and keep giving him these tablets, but do not give him these, take them back home, but continue giving him these until he finishes taking them. I did not know whether the ones the health worker told me to continue giving him were for fever or flue because I had gotten them from some Clinics, they had taken off some blood from him then they gave him the drugs, others we had just bought them but it was from another Clinic after telling them flue, cough and they gave us those tablets but the health worker told us to stop some and continue with some, i think the health worker stopped the drugs they gave to us after he was taken of blood sample

I: Ok, another thing I would like to ask is that before you came to the hospital, did you try to use any other treatment, maybe like going to traditional healers

R: I do not use that because I am a catholic, not a born-again, but I am a staunch Catholic, I am rarely taken by different things, because I did not take too long with that child when he became sick, I was quick to bring him here to the hospital. I continued praying to Mother Mary to have mercy on me, and my fellow Catholics prayed for me, they organised prayers for me, I did not go anywhere else apart from those who pray for him, and the hospital. There used to come people who prayed for patients. If it were born again, they could pray for him, or Catholic or Muslim, anyone who used to come to pray for my child could pray for him, because we all worship the same God.

I: So, how was your experience with prayer? Did it help?

R: Yes, it helped me so much because even the day they discharged us, there came something holding his neck we were still at the hospital, apart from me a caregiver, there was another lady she had also brought her child but she was also a Catholic coming from

Kampala, but her child was staying in Kabale BugonzI, but we used to pray with her. There was a gentle man, they brought him speaking vulgar words, but they told us he was a church elder for Born Again church but he came and prayed for my child when that thing came, he prayed for him until when it left up to now it has never come back, I am sure that even those prayers are so much important.

I: You talked about something, because I am also going to ask you, what do you think caused mental illness to your child?

R: I think maybe because her mother was also a Mental patient, or it may also cause it, he was her firstborn, I do not know whether it can also cause him to become mentally sick

I: Ok, when you look at other children who are in his age group, in the neighbourhood or in the community, and you see him in the same age group but the illness is limiting him to work, you, as a caretaker, how do you feel?

R: That thing, I continue to give it to God and also continue praying for such children so that they do not get an illness like his. They are his friends, they come and sit there with him, and they converse with him. I tell them to pray for him

I: Let’s go in the future, when you think about it, as you think about MM, don’t you get worried sometimes?

R: I say that God will heal him, and he will become well

I: What about work, marriage?

R: About work, because he told me he can no longer manage work which require a lot of energy, I think it is due to these drugs because they said these drugs makes people weak, I say maybe its drugs making him weak but we pray to God he will stabilise gain and get his energy back

I: What about when you think about things like marriage, family, and beginning a home?

R: I have not yet thought about it, it has not even crossed my mind, no

I: Ok, can you tell me the most challenging situation you have ever experienced while taking care of that child?

R: Since childhood or these other things?

I: About the illness

R: As I have told you, I did not stay with him too long when he became sick, I was quick to bring him to the hospital, and I did not face any big challenges

I: Except what you have explained

R: Yes

I: As we are about to conclude, what message can you give caretakers of adolescents with the kind of illness like that which MM has?

R: Caretaker of adolescents who have mental illness like that of MM, to be calm, and to trust God because sometimes you may think your child has been bewitched, yet the child was not bewitched.

Hurrying up to take the child to the hospital because me I was quick to bring him to the hospital, yes, they should be quick to go to the hospital.

To be calm working together with others because even if your siblings or relatives have not come, your friends are the ones who help you with the child when he is sick, even when you are not around, neighbours can help you to be of him well and they keep him.

Also, being patient because those children need patience

Also putting in effort to give them medicine, not to stop taking medicine, it’s a very important thing, I saw it when we were admitted here, those who bring back the children when they had stopped taking the medicine and they had relapsed, so to encourage them to be taking their medicine, mostly encouraging them to take their medicine because medicine is life, when you stop giving him medicine to the child, you may find that they have severely become mentally sick and eating from dust bins, even if you pray or do what, but as long as they do not stop taking medicine

I: Ok, anything you would like to explain about your experience as you are taking care of him?

R: Generally, I have not suffered so much because my siblings have been there with me, money is the main challenging thing in treatment but if you work together, one with 5,000 shs and sends it, one says let me sell my coffee, we get challenges but if you work together you win. I have not experienced a lot of challenges because even now, they took him to stay with him to see how he is because I used to stay with him because this side he was just staying home, but before he used to go to work but reached a point when he stopped going, he never wanted to go back to work

I: Let me ask this, when they took him, did you see it as if they took that burden from you, or what?

R: What I am worried about is that they may be lazy about him taking medicine since they are born again, you understand that, that one hurts me

I: Do you talk to them?

R: Yes, I talk to them, they tell me, he is now in our hands, leave us

I: That is what they told you?

R: Yes

I: They did not tell you what they do

R: They did not, they say he is in our hands, you treated him and he recovered, do not think that MM is mentally sick, should we give him to you on the phone and you talk to him? MM is mentally well, he recovered, but we also understand we do not want him to relapse again because it makes us feel bad, like that

I: Let us hope that they are on the cause

R: Because they are born again, at first, I had refused to give him to them but he told me I am going, he decided for himself, you understand that, and I told him MM, will you take the medicine, he said mother, I will take it, but I am going, I am not staying here. I had refused to go because when I was here, I saw children being brought back when they had stopped taking medicine, after a short period, they brought them back, and they had stopped taking medicine. I get worried about that, they did not take a burden away from me because my child was not a burden, no, I would have stayed with him

I: Ok, thank you so much, thank you for responding to the questions, I request we stop here recording
