# Supplementary material for: Lived experiences of informal caregivers of adolescents with bipolar disorder at Masaka regional referral hospital
Source: PLOS Ment Health. 2025 Jul 1;2(7):e0000351. doi: 10.1371/journal.pmen.0000351 (PMC12798243; doi:10.1371/journal.pmen.0000351)
Supplement: Attachment — Submitted filename: Response to Reviewers.doc [file pmen.0000351.s003.doc]

**RESPONSE LETTER TO THE EDITOR AND REVIEWER(S) COMMENTS**

| **Section** | **Comments** | **Response** | Line number |
| --- | --- | --- | --- |
| Journal Requirements: | 1. Your manuscript is missing the following sections: Result. Please ensure these are present, and in the correct order, and that any references to subheadings in your main text are correct.    2. In the online submission form, you indicated that Due to confidentiality requirements, the datasets created and analyzed for this work are not publicly available; however, upon reasonable request, the corresponding author can provide them in an aggregated and deidentified format.    All PLOS journals now require all data underlying the findings described in their manuscript to be freely available to other researchers, either  1. In a public repository,  2. Within the manuscript itself, or  3. Uploaded as supplementary information.  Please add a list of legend for your Supporting Information file after the References list. | This has been included in the manuscript  This has been revised as follows: All the data underlying the findings described in this manuscript are freely available to other researchers. The data have been uploaded as supplementary information together with the manuscript.  A list of legends for the Supporting Information file has been added | 181  Line 638-641 |
| **Reviewer #1: General comments and some key concerns:** | | | |
| 1. | It is an interesting study that is giving an insight on the “Lived experiences of Informal caregivers of Adolescents with Bipolar disorder at Masaka Regional Referral Hospital”. However, there are some issues that need to be addressed as indicated. | Thank you so much for this comment | ---- |
| 2. Abstract | - The authors should follow the journal guideline format in writing the abstract - The authors need to summarize the key finding on the lived experiences of Informal caregivers of Adolescents with Bipolar disorder. Some of the findings in the conclusion should have been in the results section. - In the conclusion, what are the key issues that can be concluded from the findings of the study | The journal guidelines format has been followed, and the key findings on the lived experiences of Informal caregivers of Adolescents with Bipolar disorder (BD) are included | Line 32 - 36 |
| 3. Introduction | - Line 43: The authors should indicate where the prevalence of Bipolar disorder is increasing - Line 45: The authors state that the prevalence of Bipolar disorder is scarce in Uganda, however, there many publications in this area in Uganda, and even in Butabika National referral hospital - Studies at the Butabika National Psychiatric Referral Hospital indicate that bipolar disorder is the most common diagnosis among patients seeking treatment, with estimates of up to 66.4% of cases | **-** This has been addressed as ‘Europe’ as the meta-analysis was for studies conducted in Europe  **-** This has been addressed, and the prevalence of bipolar disorder noted. However, these studies focused mainly on adults; thus, data on the prevalence of BD among adolescents is still scarce. | Line 43  Line 45 - 47 |
| 4. Methods | • In this sub-section, the authors should include the following sub-section; study population or unit • Line 119: The authors should replace “The interview guide” with “interview tool”; and the authors should explain why the pre-tested with two caregivers of adolescents with BD were done at Kiyumba HC IV. • How did the caregivers know that it was Bipolar disorder and not any other disorder? • The authors should explain how the data managed and quality control was achieved • Line 124: The authors should write data analysis | - A subsection of the Study population has been included  - ‘The interview guide’ has been replaced with ‘interview tool’ as advised.  - Kiyumba HC IV was chosen because it is within the same geographical location as the study area, and it also offers mental health services.  - The adolescents had been diagnosed with Bipolar disorder by Psychiatric Clinical Officers trained, certified, and licensed by the Uganda Allied Health Professionals Council.  - A section on data management and quality control has been included  - The word ‘Data analysis’ has been used instead of ‘analysis’ | Line 100 - 104  Line 118  Line 136 - 140  Line 166  Line 143 - 161  Line 162 |
| 5. Results | • Line 141: 3. “Findings” should be “Results” • The authors should elaborate more on the demographic characteristics of the study participants including level of education, occupation if possible, religion, type of family since these can influence the outcomes!! • What is the unit of age in Table 1 – Line 143? • Were there BD patients on the treatment, and given that it is almost chronic disease and therefore requires prolonged treatment, did the complexity of treatment and adherence not an issue!!! | - The word ‘Results’ has been used instead of ‘Findings’  - The demographic characteristics have been elaborated on further  - The unit of age was years, and it has been included in Table 1  - The adolescents were on treatment since BD is a chronic condition. However, in the interview, we didn’t focus on the complexity of treatment | Line 180  Line 182  Line 182 |
| 6. Discussion | • Line 303: what are the traditional treatments, these individuals seek for? • The limitations should be based on methodological utilized to answer the objectives of the study and not as it is stated | The participants narrated pursuing traditional treatments, such as traditional healers and witch doctors  - The limitation section has been revised as advised | Line 344 – 345  Line 474 - 481 |
| 7. Conclusion | • Recommendation should be combined in the conclusion | The recommendation has been incorporated into the conclusion | Line 488 - 490 |
| 8. References | • The text should be uniform all through and not a mixture of upper case and lower case. See the Journal guideline | This has been addressed following the journal guidelines | Line 503 |
| **Reviewer #2: comments** | | | |
| Title | - Use title case for academic consistency (capitalize major words). - Avoid line breaks mid-title (unless it's a formatting constraint in the journal style) | - Thank you for these comments  - The major words in the title have been capitalized | Title page |
| Abstract | Replaced casual/ambiguous phrases like "circle of support" with more precise terms like "support networks." Changed "struggles" to "emotional burden" for better academic tone. Improved flow by reducing passive constructions and redundancy (e.g., "coupled with" → "compounded by"). | - The theme ‘circle of support’ has been replaced with ‘Support networks’ as advised  - Thank you for this comment on changing the theme "Hidden Struggles of caregiving" to "emotional burden". We didn’t change this because we felt that the theme ‘emotional burden’ may not reflect well the subthemes of financial burden, resistance, and exhaustion.  - The phrase ‘coupled with’ has been changed to ‘compounded by’ | Line 31  Line 33 |
| Introduction | Replaced semi-colons inappropriately used in lists with commas. Grouped sequential references (e.g., 13–15) for readability. Clarified the flow between citations and statements. Replaced casual language with more formal equivalents (e.g., "play a pivotal role" → "play a critical role"). For enhance the quality of your article please use these references: <https://www.jpcmed.com/article_153234.html> <https://brieflands.com/articles/ijpbs-123998.html> Reordered some content (e.g., defining informal caregiving earlier) for better narrative structure. | - This has been addressed throughout the manuscript  - Sequential references have been grouped for easy readability as advised  - The flow between citations and statements has been clarified  - Causal language has been replaced with more formal language | Line 52 |
| Methodology | Reworded “to gain deeper insights” → “to explore and interpret” for more formal and objective tone. “Utilized” replaced with “combined” for clarity and conciseness. • Made the connection between van Manen’s philosophy and the study’s goals clearer. • Provided a more natural explanation of the phrase “world as world” by integrating it with its philosophical context. • Smoothed transitions between study design and setting. • Grouped related geographic information for easier readability. • Replaced casual phrasing like “had accompanied adolescents for drug refills” with more formal alternatives like “for medication refills.” • Changed “specifically directed toward” to “specifically tailored to” for a smoother tone. • Added punctuation and corrected minor grammar issues (e.g., spacing, sentence fragments). • Clarified the role of the assistant and counselor to avoid ambiguity. • Broke long sentences into clearer components. • Ordered information for a natural progression: tool development → participant recruitment → ethical consent → interview conduct → safety considerations → confidentiality. | - The phrase “to gain deeper insights” has been changed to “to explore and interpret” as advised.  - The word ‘utilized’ has been replaced with ‘combined’  - Van Manen’s philosophy has been integrated in the discussion of the results  - A natural explanation for the phrase “world as world” has been provided as ‘world as a horizon within which all our experiences take shape’  - Causal phrases such as “had accompanied adolescents for drug refills” have been replaced with more formal alternatives like “for medication refills.” - The phrase “specifically tailored to” has been adopted instead of “specifically directed toward.” - Punctuation and grammatical errors have been rectified as advised  - The roles of both the counsellor and research assistant have been clarified as advised | Line 91  Line 92  Line 93 – 94  Line 128  Line 120  Line 132 - 136 |
| **Reviewer #3:** | | | |
|  | Thank you for the opportunity to review this thoughtful and socially relevant manuscript. Your study contributes meaningfully to the limited body of literature on the lived experiences of caregivers of adolescents with bipolar disorder in low-resource settings. The cultural and emotional complexity of caregiving is well captured, and the participant quotations enrich the narrative, giving voice to often-unheard experiences. | Thank you so much for this comment |  |
| 1. Theoretical and Conceptual Framing | The manuscript would benefit from a stronger theoretical foundation. While van Manen’s phenomenological framework is mentioned, it is not sufficiently discussed in terms of how it informed data interpretation and writing. Consider elaborating on how this framework shaped your lens and analytic decisions. | We have revised the discussion section and have employed van Manen’s phenomenological framework to interpret and discuss the results | From Page 14 |
| 2-Methodological Rigor: | The claim of data saturation is not substantiated. Please clarify how saturation was determined (e.g., thematic redundancy, use of saturation grids). Describe how trustworthiness was ensured. did you use triangulation, member checks, audit trails, or inter-coder reliability?  The translation/back-translation process needs more detail. How was accuracy ensured? Were transcripts validated? | - Saturation was achieved through thematic redundancy, and this has been well explained.  - The process of Trustworthiness has been included as advised.  - To ensure consistency, the interview tool was translated into Luganda by two experienced mental health research assistants from the Nursing department and then back-translated into English by two other experienced research assistants. At first, they worked independently, but later compared the original and back-translated versions of the research tool. This helped to resolve any discrepancies through discussion and consensus to ensure consistency and proper flow of the tool. | Line 168 – 171  Line 147 – 161  Line 120 - 126 |
| 3-Researcher Reflexivity | Phenomenological research requires a discussion of the researchers' positionality and potential influence on data interpretation. This is missing and should be added. | As part of ensuring the researchers’ reflectivity, the following was noted: the author is a practicing mental health nurse based at a primary health care facility, with direct experience providing treatment to individuals with mental health disorders. This professional background provided an insider perspective on the challenges faced by informal caregivers of adolescents with BD. While this familiarity offered valuable contextual insight, it also posed potential interpretive biases. To address such bias, peer participation and member checking during data analysis were employed to ensure critical self-awareness and uphold the integrity of the participants' lived experiences. | Line 154 - 161 |
| 4-Sample Representation | All caregivers were female (mothers, grandmothers, one aunt). While understandable, this should be acknowledged as a limitation, and the absence of male voices discussed. | Thank you for this comment. It has been noted as one of the study’s limitations | Line 477 - 478 |
| 5-Depth of Analysis in Discussion: | The discussion section largely restates results. Consider expanding the analysis by connecting findings to broader regional/global literature, including caregiver burden models, cultural stigma, and task-sharing in low-resource mental health settings. Please discuss how community support might be better integrated into formal care systems or leveraged for policy development. | Thank you for this comment. The discussion on community support has been adjusted as advised. | From line 332 |
| Minor Suggestions: | 1-Please proofread the manuscript for grammatical errors and clarity. Some sentences (e.g., in the discussion) are long or awkwardly phrased. 2-Tables need better formatting and clearer headings. 3-In the abstract and introduction, please specify more clearly that this is a Ugandan setting and define BD earlier in the abstract. 4- Please expand slightly on ethical safeguards (e.g., data storage procedures) for completeness. | These have been worked on throughout the manuscript |  |
